# Total Synthesis of 8-Hydroxy-dihydroergotamine, the Major Human Metabolite of Dihydroergotamine

**DOI:** 10.3390/molecules31091547

**Published:** 2026-05-06

**Authors:** Manuel Monerris Mascaro, Alistair P. Henderson, Marta Drozdowska, Rachel Richardson, Dylan Nagel-Savage, Michael J. Hall, Alexandra Longcake, Lina Mardiana, Bernard T. Golding

**Affiliations:** 1 Sterling Newcastle, The Biosphere, Drayman’s Way, Newcastle Helix, Corporation Street, Newcastle upon Tyne NE4 5BX, UK; manuel.monerris@sterlingpsl.com (M.M.M.); marta.drozdowska@sterlingpsl.com (M.D.);; 2 School of Natural and Environmental Sciences—Chemistry, Bedson Building, Newcastle University, Newcastle upon Tyne NE1 7RU, UK; 3 Indicatrix Crystallography Ltd., Newcastle University, Newcastle upon Tyne NE1 7RU, UK; 4 Department of Chemistry, Universitas Indonesia, Depok 16424, Indonesia

**Keywords:** 8-hydroxy-dihydroergotamine, pentadeutero-8-hydroxy-dihydroergotamine, synthesis, stereochemistry, dihydroergotamine, metabolism, migraine

## Abstract

8-Hydroxy-dihydroergotamine is the major human metabolite of the anti-migraine drug dihydroergotamine and is required, along with a stable isotope-labelled derivative, to aid metabolic studies. An efficient, scalable synthesis of the unlabelled compound is described via the coupling of dihydrolysergic acid to the tricyclic amino compound (2*R*,5*S*,8*R*,10a*S*,10b*S*)-2-amino-5-benzyl-10b-hydroxy-8-methoxy-2-methyltetrahydro-8*H*-oxazolo[3,2-*a*]pyrrolo[2,1-*c*]pyrazine-3,6(2*H*,5*H*)-dione. The tricycle was obtained by a convergent synthesis combining precursors from suitably protected L-glutamic acid and L-phenylalanine, and 2-bromo-2-methylmalonic acid. For the labelled molecule, the tricyclic precursor contained a pentadeutero benzyl group derived from [2,3,4,5,6-^2^H_5_]L-phenylalanine. Considerable experimentation was required to achieve optimal activation of dihydrolysergic acid for efficient amide formation with the tricycle’s amino function affording 8-methoxy-dihydroergotamine. The stereochemical integrity of an intermediate in this synthesis, ethyl (2*R*,5*S*,8*R*,10a*S*)-5-benzyl-10b-hydroxy-8-methoxy-2-methyl-3,6-dioxooctahydro-8*H*-oxazolo[3,2-*a*]pyrrolo[2,1-*c*]pyrazine-2-carboxylate, was validated by crystal structure analysis. Acid-catalysed hydrolysis of 8-methoxy-dihydroergotamine gave 8-hydroxy-dihydroergotamine. Pentadeuterated 8-hydroxy-dihydroergotamine was obtained in an analogous manner from [2,3,4,5,6-^2^H_5_]L-phenylalanine. Both 8-hydroxy-dihydroergotamine and its ^2^H_5_-derivative were obtained as an equilibrating mixture of C-8 epimers (diastereomers), with the major isomer having (*R*)-configuration according to ^1^H NMR analysis. The syntheses described enable the routine synthesis of 50–100 mg quantities of each target molecule.

## 1. Introduction

The adverse impact on humans of the toxic properties of ergot alkaloids produced by fungal infection of cereal crops, notably rye by *Claviceps purpurea*, was already known to the ancient civilizations of Egypt and Greece [1]. Over time, multiple injuries and deaths occurred from consumption of the black sclerotia (‘spurs’) in grains of infected rye. Paradoxically, therapeutic applications of this natural material were also discovered as early as 1100 BCE [2,3], with the Chinese Pharmacopoeia recording the application of crude extracts in obstetrics for inducing childbirth [1]. Rational progress in the understanding of the toxicology and potential medical applications of the ergot alkaloids required the isolation of pure samples, which was first achieved by Stoll [4,5] for ergotamine (**1**) following pioneering work by Tanret [6], Barger [7,8], and others. This substance is a so-called ‘ergopeptine’ with a peptide-like moiety attached to the ‘ergoline’ lysergic acid. Ergotamine, and especially dihydroergotamine (**2**, DHE), emerged as effective drugs for treating migraine, which afflicts over 1 billion sufferers worldwide [9,10,11]. These compounds function by acting as 5-HT_1B_ receptor agonists, modulating the activity of serotonin in the central nervous system [12,13].

**Scheme 1:**
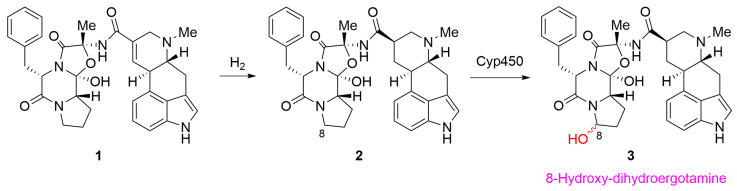
Structures and interconnections of ergotamine (**1**), dihydroergotamine (**2**) and 8-hydroxy-dihydroergotamine (**3**).

DHE was first synthesised in 1943 [14,15] by reduction of ergotamine, and was commercialised as dihydergot for the treatment of vascular headaches and orthostatic hypotension [3]. Compared to ergotamine, DHE is distinguished by its greater efficacy and relatively lower toxicity [10,11] and remains a frontline medicine for the treatment of severe migraine. Effective delivery has been a major issue that was recently surmounted with dihydroergotamine mesylate powder (STS-101 or Atzumi^TM^), an FDA-approved formulation enabling nasal delivery [16]. Optimisation of DHE delivery requires pharmacokinetic studies, which are aided by monitoring metabolites. The dominant metabolite in humans is 8-hydroxy-dihydroergotamine (**3**) [17] formed by the action of a cytochrome P450 monooxygenase on DHE (Scheme 1). Note that 8-hydroxy-dihydroergotamine is not directly related to the naturally occurring, so-called 8-hydroxyergotamine, where the numbering refers to the lysergic acid part [18].

The highly unusual ‘aminocyclole’ [19,20] structural feature of ergotamine and related alkaloids was a challenge not only for structure elucidation [21,22], requiring precise validation by X-ray analysis [23,24,25], but also for synthesis [26,27]. An additional challenge for the synthesis of 8-hydroxy-dihydroergotamine is the existence of the compound as a mixture of epimers (diastereomers) owing to ‘ring-chain tautomerism’ at C-8 [17,19,20].

## 2. Results and Discussion

We describe a total synthesis of 8-hydroxy-dihydroergotamine and a pentadeutero derivative, which, for the first time, makes these compounds available in >50 mg quantities. We chose total synthesis rather than the possibility of direct hydroxylation of ergotamine or DHE, because efficient, selective oxygen insertion at C-8 is not currently a realistic prospect in such a complex structure, and in any case, if achievable, would still require a multistep synthesis to access isotopically labelled material. In one attempt at chemical oxidation, subjecting dihydroergotamine to *m*-chloroperbenzoic acid in the presence of Jacobsen’s catalyst gave 5- and 11-hydroxydihydroergotamine but no 8-hydroxy derivative [28]. Although 8-hydroxy-dihydroergotamine can be obtained either by biotransformation of DHE using a bacterium from the genus *Rhodococcus* sp. [29] or from the action of rat liver microsomes on DHE [30], these methods are unsuitable not only for scale-up, but also for the synthesis of isotopically labelled material.

**Scheme 2:**
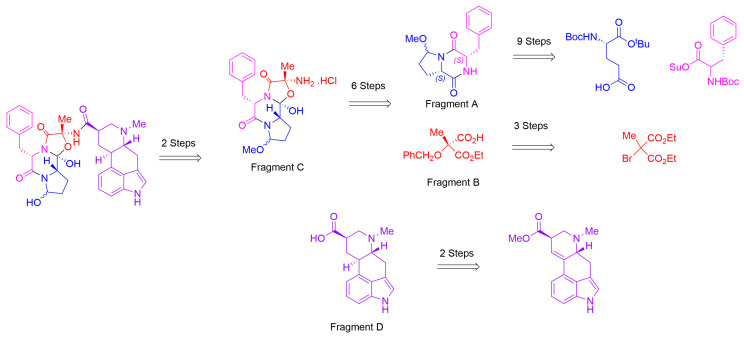
Retrosynthetic analysis.

Our strategy towards 8-hydroxy-dihydroergotamine, summarised retrosynthetically in Scheme 2, required combining a diketopiperazine (Fragment A) with a derivative of malonic acid (Fragment B), followed by several steps to Fragment C, which was joined with dihydrolysergic acid (Fragment D) to afford 8-methoxy-dihydroergotamine. This route parallelled Hofmann’s synthesis of dihydroergotamine [26], although the synthesis of the functionalised diketopiperazine (Fragment A) was not simply constructed from phenylalanine and proline, as in his synthesis. Further, the ultimate 8-hydroxy group could not be carried through the synthesis without protection as its methoxy ether, primarily because ring-chain tautomerism readily equilibrates the 8-hydroxy epimers, leading to mixtures of compounds more difficult to purify and characterise. The major additional challenge of the synthesis was the coupling of the peptide moiety fragment C to dihydrolysergic acid (Fragment D). Although this step is an ostensibly trivial amide formation between amino and carboxyl precursors, the amino component (Fragment C) is highly unstable, readily undergoing an irreversible fragmentation. This process renders the amino component difficult to trap efficiently, even with a highly activated carboxyl component. Hofmann surmounted this problem in his synthesis, but despite extensive trials, we never achieved the level of efficiency for amide formation he reported [26].

**Scheme 3:**
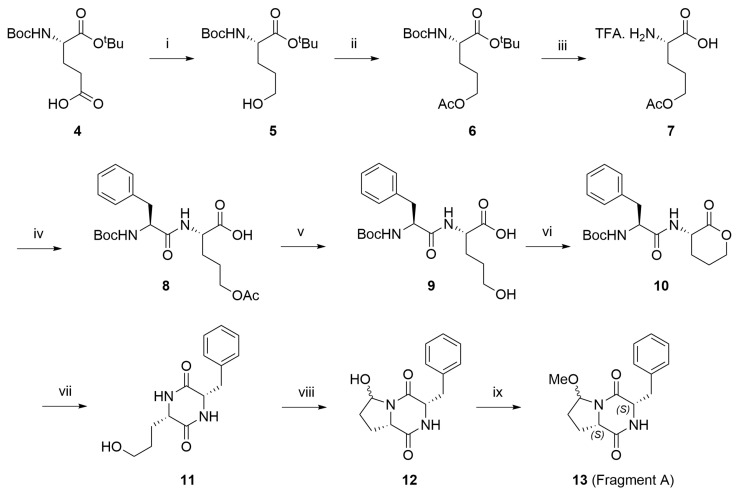
Synthesis of Fragment A (**13**). i. Ethyl chloroformate, Et_3_N, THF; Sodium borohydride, aq. THF; ii. MeCOCl, pyridine, CH_2_Cl_2_, rt; 79% over 2 steps; iii. TFA, Et_3_SiH, CH_2_Cl_2_; iv. Boc-Phe-OSu, Hünig’s base, DMF; v. NaOH/MeOH, 68% over 3 steps; vi. EDCI.HCl or HATU, Hünig’s base, CH_2_Cl*_2_*, 86%/75%; vii. TFA, Hünig’s base, DMF, 75%; viii. IBX, DMSO, 68%; ix. PTSA, MeOH, 85%.

Fragment A containing a masked 5-hydroxyproline moiety was obtained from protected L-glutamic acid and L-phenylalanine. Reduction of the mixed anhydride derived from the carboxyl group of Boc-glutamic acid mono *tert*-butyl ester **4** with sodium borohydride [31] gave primary alcohol **5** (Scheme 3), which was acetylated to **6**. Concomitant Boc and *tert*-butyl deprotection using trifluoroacetic acid in the presence of triethylsilane gave **7** in excellent yield as its trifluoroacetic acid salt. Coupling to the succinimidyl ester of Boc-phenylalanine (Boc-Phe-OSu) in dimethylformamide in the presence of Hünig’s base (*N*-ethyl-*N*-isopropylpropan-2-amine) afforded dipeptide **8**. Deacetylation of **8** with sodium hydroxide in methanol followed by treatment of the resulting carboxylic acid **9** with 1-ethyl-3-(3-dimethylaminopropyl)carbodiimide (EDCI) gave lactone **10** in excellent yield. Removal of the Boc group gave diketopiperazine **11** via intramolecular attack of the liberated amino group on the lactone. Several methods of oxidation (e.g., Dess–Martin; dimethyl sulfoxide (DMSO)/dicyclohexylcarbodiimide; Pfitzner–Moffatt oxidation, pyridinium chlorochromate in dimethylformamide, pivaloyl chloride/triethylamine in DMSO [32]) were trialled to obtain the corresponding aldehyde from **11**. Oxidation of diketopiperazine **11** with 2-iodoxybenzoic acid (IBX) in DMSO [33,34] was found to be the best method to afford the aldehyde, avoiding the formation of by-products observed with other methods. The aldehyde underwent ring-chain isomerism in situ to bicyclic compound **12**, obtained as a mixture of epimers in good, combined yield. Finally, protection of **12** using *p*-toluenesulfonic acid in methanol [35] gave Fragment A **13** as a 7:3 mixture of epimers.

**Scheme 4:**
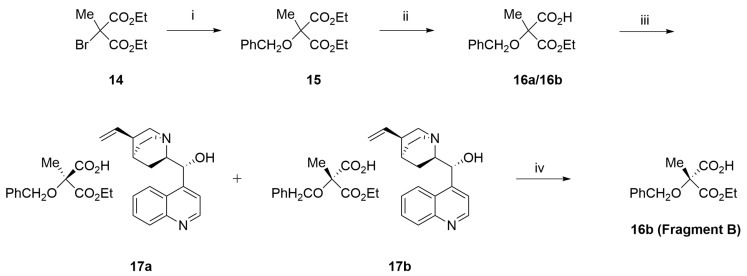
Synthesis of Fragment B (**16b**). i. Benzyl alcohol, NaH, toluene; ii. KOH/EtOH, 47% over 2 steps; iii. Cinchonidine, dimethyl carbonate (fractional crystallisation to afford **17b**) iv. 1 M HCl.

The synthesis of Fragment B had already been reported via alkylation of diethyl 2-hydroxy-2-methylmalonate with benzyl bromide [36]. Our approach to Fragment B started from commercially available diethyl 2-bromo-2-methylmalonate (**14**, Scheme 4). Nucleophilic substitution of bromide **14** with sodium benzyloxide gave benzyl ether **15**, which was subjected to partial hydrolysis with potassium hydroxide to racemic hemiester (**16a**/**16b**). This racemate was mixed with cinchonidine in dimethyl carbonate, affording diastereomeric salts **17a**/**17b**, which were resolved by fractional crystallisation. In this way, Fragment B (**16b**) was obtained in at least 60–65% enantiomeric excess according to chiral HPLC analysis and polarimetry [37].

**Scheme 5:**
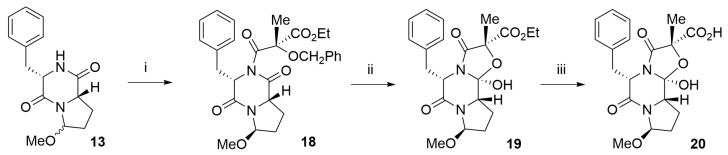
Synthesis of carboxylic acid **20**. i. **16b**, SOCl_2_, DMF, CH_2_Cl_2_ then **13**, pyridine/CH_2_Cl_2_, 67%; ii. H_2_, Pd-C, MeOH, 59%; iii. Aq. NaOH/THF, 99%.

**Figure 1:**
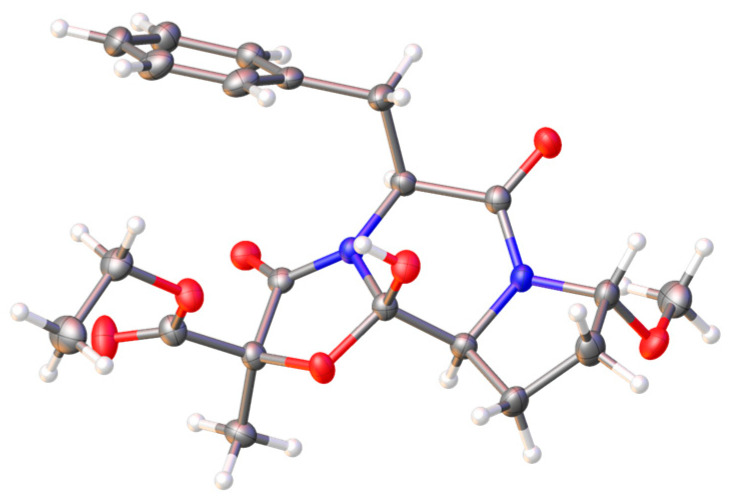
Crystal structure of compound **19** (anisotropic displacement parameters shown at 50%).

Coupling of Fragment A to Fragment B was achieved via the acyl chloride of Fragment B (**16b**), giving a mixture of diastereomers differing at the methyl- and methoxy-substituted positions, with the diastereomer **18** shown in Scheme 5 being dominant. Cleavage of the benzyl group of **18** by catalytic hydrogenolysis was followed by cyclisation to a mixture of epimers, from which tricycle **19** was separated as a single isomer by chromatographic purification. Following crystallisation of **19** from ethyl acetate, the structure and absolute stereochemistry were confirmed by single-crystal X-ray diffraction analysis (Figure 1). Tricycle **19** crystallised in the orthorhombic *P*2_1_2_1_2_1_ space group, with the notable features of the crystal structure being the validation of the aminocyclole structure and the (*R*)-configuration at both C-2 and C-8.

Base-induced hydrolysis of ester **19** gave carboxylic acid **20** in excellent yield. Numerous approaches were explored for converting acid **20** into the corresponding acyl azide **21,** and hence carbamate **22** via an intermediate isocyanate. Model studies were performed with the epimer (at C-2) of **20**, which achieved straightforward conversion into the epimer (at C-2) of **21** using diphenylphosphoryl azide (DPPA) and quenching the isocyanate with benzyl alcohol. However, much effort was needed to optimise preparation of acyl azide **21**, with numerous reagents being trialled: thionyl chloride/sodium azide; DPPA; 1-[bis(dimethylamino)methylene]-1*H*-1,2,3-triazolo[4,5-*b*]pyridinium 3-oxide hexafluorophosphate (HATU)/sodium azide [38]; trichloroacetonitrile/triphenylphosphine/sodium azide [39]; ethyl chloroformate or isopropyl chloroformate/sodium azide [40]; hydroxylamine hydrochloride/1,1-carbonyldiimidazole [41]; and oxalyl chloride with the sodium salt of **20** [26]. The failure to generate azide **21** smoothly is probably due to steric encumbrance in the vicinity of the carboxyl function of **20**, which is less in its diastereomer.

**Scheme 6:**
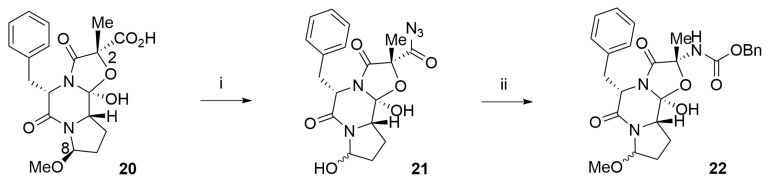
Synthesis of benzyl carbamate **20**. i. Oxalyl chloride, cat. DMF/CH_2_Cl_2_ then Bu_4_N N_3_, acetone, 52%; ii. 1. Neat benzyl alcohol, 130 °C. 2. PTSA, MeOH, 63% over 2 steps.

The best conditions (Scheme 6) for obtaining carbamate **22** were found to be via preparation of the acyl chloride of **20** using oxalyl chloride in dichloromethane with catalytic dimethylformamide [42], followed by addition of tetrabutylammonium azide in acetone at low temperature. This procedure gave the acyl azide **21** as a mixture of epimers at C-8 in 52% yield. However, the acyl azide was very unstable and difficult to characterise fully by ^13^C NMR. Nevertheless, Curtius rearrangement in neat benzyl alcohol at high temperature [26] with compound **21**, followed by subsequent exchange of the hydroxy group to methoxy using *p*-toluenesulfonic acid in methanol, afforded carbamate **22** as a 7:3 mixture of epimers that were separated by chromatography.

**Scheme 7:**
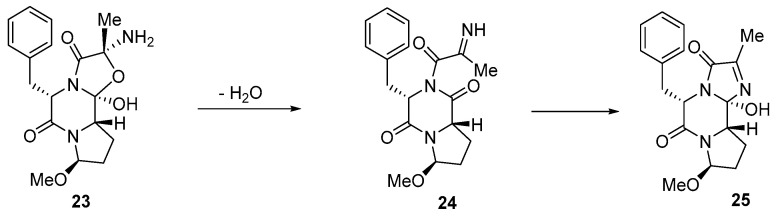
Degradation pathway for aminocyclole **23**.

**Scheme 8:**
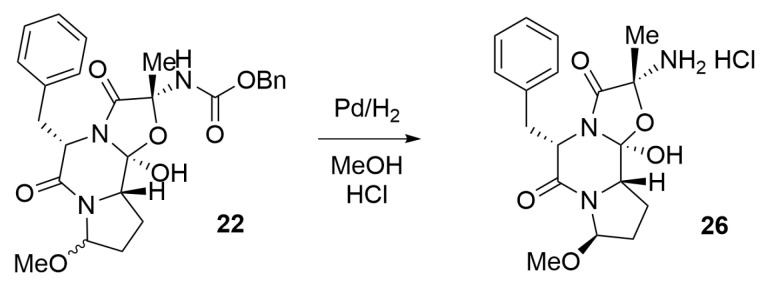
Catalytic hydrogenation to aminocyclole hydrochloride (Fragment C, **26**).

It was reported [43] that the des-methoxy analogue of aminocyclole **23** derived by deprotection of the corresponding carbamate is extremely unstable as the free base giving ‘pyroergotamine’, i.e., the des-methoxy version of 8-methoxy-pyroergotamine (**24**, Scheme 7). In our initial attempts to replicate this chemistry, we found that compound **23** was converted into tricycle **25**, possibly *via* imine **24.** To minimise this degradation pathway, cleavage of the carbobenzoxy group of **22** by catalytic hydrogenation (Scheme 8) was performed in the presence of HCl [26], thus trapping compound **23** as its hydrochloride salt **26**. Despite this precaution, high purity of compound **26** was never achieved for either epimer of **22**, which both gave primarily a single isomer **26** owing to HCl-catalysed equilibration.

**Scheme 9:**
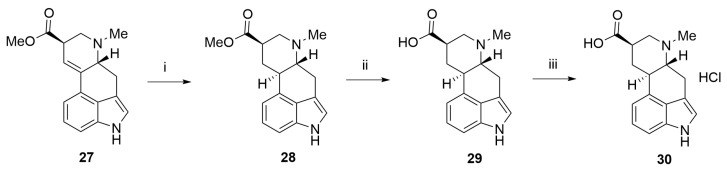
Synthesis of dihydrolysergic acid hydrochloride (Fragment D, **30**). i. H_2_, Pd-C, MeOH, 35 °C, 67%; ii. NaOH/MeOH, 40 °C, 74%; iii. 1 M HCl, aq. MeCN, 81%.

For the synthesis of Fragment D (Scheme 9), stereoselective catalytic hydrogenation of D-lysergic acid methyl ester **27** in methanol [44] gave 9,10-dihydrolysergic methyl ester acid **28**, which was saponified with aqueous sodium hydroxide in methanol at 40 °C [45] to afford 9,10-dihydrolysergic acid **29**, isolated as its hydrochloride **30**.

**Scheme 10:**
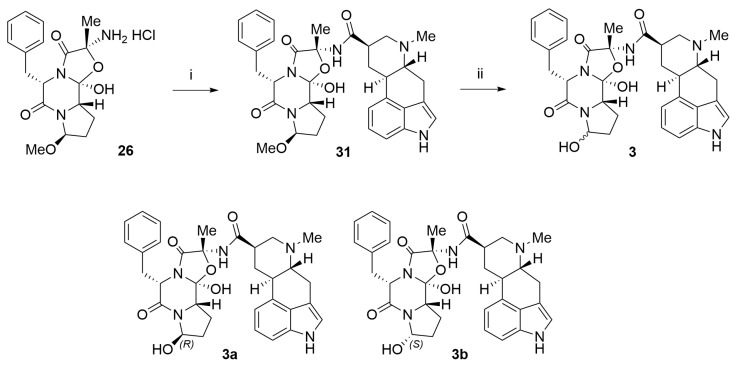
Synthesis of 8-Hydroxy-dihydroergotamine **3**. i. **30**, HATU, Hünig’s base, DMF, r.t. then **26**, 21% over 2 steps from **22**; ii. 1 M HCl, MeCN, 90%.

Treatment of 9,10-dihydrolysergic acid **29** with thionyl chloride, PCl_5_, POCl_3_, a mixture of PCl_5_ and POCl_3_, *N*′-bis(tetramethylene)formamidinium hexafluorophosphate (BTFFH) [46], or trifluoroacetic acid and trifluoroacetic anhydride in DMF [47], followed by the addition of compound **26** in the presence of Hünig’s base, all failed to give 8-methoxy-dihydroergotamine **31** [26,48,49,50]. However, when HATU was used as a coupling reagent for **26** and **29**, 8-methoxy-dihydroergotamine **31** was obtained after chromatographic purification, followed by semi-preparative HPLC, in moderate yield. Cleavage of the methoxy group was achieved by treatment with 1 M aqueous HCl in acetonitrile, giving 8-hydroxy-dihydroergotamine **3** (3:2 mixture of epimers **3a** and **3b**) as its mixed hydrochloride/formate salt (2:3 ratio) after reversed-phase chromatographic purification (Scheme 10). 8-Hydroxy-dihydroergotamine from rat liver microsomes was reported to be a 78:22 mixture of epimers denoted as 8α and 8β [30]. We found that the epimers could be separated by semi-preparative HPLC but then rapidly equilibrated in aqueous solution in a similar manner to glucose anomers.

The synthetic 8-hydroxy-dihydroergotamine epimers were clearly differentiated in the 400 MHz ^1^H NMR spectrum in *d*^6^-DMSO, which showed the H-8 protons in ca. 3:2 ratio at δ 5.62 (br dt, *J* = 4.7, 5.0 Hz) and 5.44 (br t, *J* = 5.2 Hz), respectively. Each resonance showed coupling to the attached hydroxyl group, as well as coupling to the neighbouring methylene protons (only the relatively large couplings are given above). The C-8 hydroxyl protons were observed as doublets (2:3 ratio) at δ 6.03 (*J* = 5.1 Hz) and 6.02 (*J* = 4.9 Hz), respectively. After the addition of one drop of D_2_O to the sample in *d*^6^-DMSO, the NMR spectrum showed collapse of the double triplet to a triplet (*J* = 4.5 Hz) and the triplet to a doublet (*J* = 4.6 Hz). In CD_3_CN, the H-8 resonances appeared at δ 5.63 (major epimer) and 5.55 (minor).

**Figure 2:**
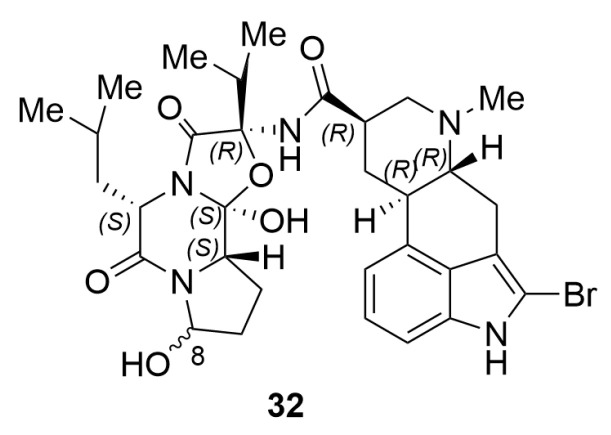
Structure of 8-hydroxybromocriptine epimers.

These NMR data agree with those published [51] for 8-hydroxybromocriptine epimers (**32**) (Figure 2), which are analogues of the 8-hydroxy-dihydroergotamine isomers. The 8-hydroxybromocriptine epimers were designated as α and β [δ 5.75 (t, H-8) and 5.70 (d, H-8) in CDCl_3_, respectively], but without assigning their absolute configurations at C-8 [51]. Compound **22** was obtained as two 8-methoxy epimers, which showed H-8 at δ 5.52 (t, *J* = 3.0 Hz) for the major and 5.18 (d, *J* = 5.2 Hz) for the minor epimer, respectively (spectra in CD_3_CN). The ^1^H NMR spectrum of compound **19** (in *d*^6^-DMSO), which has proven (*R*)-configuration at C-8, showed H-8 as a double doublet at δ 5.47 (*J* = 1.5 and 4.0 Hz). By comparison of this data with that for the single isolated epimer (**31**) of 8-methoxy-dihydroergotamine (H-8: dd at δ 5.52, *J* = 1.5 and 5.0 Hz), it can be concluded that this compound also has (*R*)-configuration at C-8. Taken together, the above data indicate that the major epimer of 8-hydroxy-dihydroergotamine also has (*R*)-configuration at C-8 (as shown in structure **3a**), whereas the minor epimer is compound **3b**.

## 3. Materials and Methods

### 3.1. General Methods

Chemicals and solvents were from commercial suppliers (mainly: Sigma-Aldrich Company Limited, The Old Brickyard, Gillingham, Dorset, SP84XT, UK; Fluorochem Limited, Rossington Park, Hadfield, Derbyshire, SK13 1QH, UK) and used without further purification. Anhydrous solvents were used when appropriate. Petrol was the fraction boiling in the range 40–60 °C. TLC used aluminium plates pre-coated with Kieselgel 60 F254, 0.2 mm (Merck, Darmstadt, Germany). Visualisation was by UV light. ^1^H/^13^C NMR spectra were recorded using solvent resonances as internal standardsat the given MHz below using a Bruker Avance III 300, Bruker Avance Neo 400, Bruker Avance III HD 500 or Bruker Avance III HD 700 spectrometer (Bruker UK Ltd., Westwood Business Park, Coventry CV4 8HX, UK) using the residual proton(s) in the deuterated solvents as internal standards. HPLC analyses were performed with a Shimadzu Prominence instrument (Shimadzu UK Limited, Wolverton Mill South, Milton Keynes, MK12 5RE, UK) with diode array detection. LC-MS analyses were performed with a Shimadzu 2010EV instrument operating in positive or negative electrospray (ESI) mode with detection at 254 nm, unless stated otherwise. Chromatographic purifications were performed with a Biotage Selekt automated chromatography system (Biotage GB Limited, Dyffryn Business Park, Ystrad Mynach, Hengoed CF82 7TS, UK) operating under either normal phase (silica column) or reversed-phase conditions (C18, silica column).

### 3.2. Synthesis of Compounds

#### 3.2.1. Tert-Butyl (S)-5-acetoxy-2-((tert-butoxycarbonyl)amino)pentanoate (**6**)

To an ice-cold solution of (*S*)-5-(*tert*-butoxy)-4-((*tert*-butoxycarbonyl)amino)-5-oxopentanoic acid (10.0 g, 33.0 mmol) in an oven-dried flask under nitrogen in anhydrous tetrahydrofuran (200 mL) and triethylamine (3.60 g, 3.28 mL, 33.7 mmol), ethyl chloroformate (3.40 g, 4.68 mL, 33.7 mmol) was added and the mixture was stirred at 0 °C for 30 min. The suspension was filtered through a Büchner funnel. The filtrate was added dropwise to a suspension of sodium borohydride (3.76 g, 99.0 mmol) in water:tetrahydrofuran (1:5, 88 mL) under nitrogen, and the mixture was stirred at room temperature for 3.5 h. The reaction was quenched with 1 M hydrochloric acid (150 mL) and extracted with diethyl ether (3 × 100 mL). The combined organic layers were washed with water (200 mL), satd. brine (600 mL), dried (MgSO_4_), and concentrated to give *tert*-butyl (*S*)-2-((*tert*-butoxycarbonyl)amino)-5-hydroxypentanoate (9.65 g) as a colourless oil. TLC: R*_f_* = 0.3 (ethyl acetate—petrol, 3:7 *v*/*v*).

To an ice-cold solution of *tert*-butyl (*S*)-2-((*tert*-butoxycarbonyl)amino)-5-hydroxypentanoate (9.65 g, 38.4 mmol) in an oven-dried flask under nitrogen in anhydrous pyridine:anhydrous dichloromethane (1:2, 139 mL), acetyl chloride was added (2.88 g, 2.51 mL, 36.7 mmol), and the mixture was stirred at room temperature for 2 h. The reaction was quenched with 1 M hydrochloric acid (150 mL) and the layers were separated. The aqueous layer was extracted with dichloromethane (2 × 80 mL). The combined organic layers were washed with 1 M hydrochloric acid (150 mL), water (150 mL), satd. aqueous sodium bicarbonate (150 mL), satd. brine (600 mL), dried (MgSO_4_) and concentrated to give a yellow oil. This material was purified using automated chromatography under normal phase conditions (gradient of 0 → 100% ethyl acetate in petrol) to give *tert*-butyl (*S*)-5-acetoxy-2-((*tert*-butoxycarbonyl)amino)pentanoate (8.61 g, 79% over 2 steps) as a colourless oil. TLC: R*_f_* = 0.5 (ethyl; acetate—petrol, 3:7 *v*/*v*). ^1^H NMR (300 MHz, DMSO-*d*_6_): δ 7.12 (d, 1 H, *J* = 7.8 Hz, NH), 4.01–3.95 (m, 2H, CH_2_O), 3.84–3.74 (m, 1 H, α-CH), 1.99 (s, 3 H, CH_3_), 1.72–1.52 (m, 4 H, 2 × CH_2_), 1.41–1.30 (m, 18 H, Boc and *tert*-butyl). ^13^C{^1^H} NMR (75 MHz, DMSO-*d*_6_): δ 171.6, 170.3, 155.5, 80.3, 78.0, 63.3, 53.9, 28.2, 27.6, 27.3, 24.8, 20.7. LC-MS (ESI positive, *m*/*z*) calcd. for C_16_H_29_NO_6_ [M + Na]^+^: 354.19, found: 354.15.

#### 3.2.2. (S)-5-Acetoxy-2-aminopentanoic Acid Trifluoroacetic salt (**7**)

A solution of *tert*-butyl (*S*)-5-acetoxy-2-((*tert*-butoxycarbonyl)amino)pentanoate (8.61 g, 26.0 mmol) and triethylsilane (15.1 g, 20.8 mL, 130 mmol) in trifluoroacetic acid: anhydrous dichloromethane (1:1, 94 mL) was stirred at room temperature overnight. The solution was azeotropically concentrated with diethyl ether and dichloromethane to give (*S*)-5-acetoxy-2-aminopentanoic acid trifluoroacetic acid salt (8.4 g) as an oily white foam that was used without further purification. ^1^H NMR (300 MHz, DMSO-*d*_6_): δ 8.35 (br s, 3 H, NH_3_^+^), 4.01 (t, 1 H, *J* = 6.0 Hz, CH_2_O), 3.96–3.88 (br m, 1 H, α-CH), 2.00 (s, 3 H, CH_3_), 1.89–1.58 (m, 4 H, 2 × CH_2_). ^13^C{^1^H} NMR (75 MHz, DMSO-*d*_6_): δ 170.9, 170.4, 158.7, 158.3, 63.1, 51.7, 26.7, 23.9, 20.7. LC-MS (ESI positive, *m*/*z*) calcd. for C_7_H_13_NO_4_ [M + H]^+^: 176.09, found: 176.05.

#### 3.2.3. (S)-5-Acetoxy-2-((S)-2-((tert-butoxycarbonyl)amino)-3-phenylpropanamido)pentanoic Acid (**8**)

To a solution of (*S*)-5-acetoxy-2-aminopentanoic acid trifluoroacetic acid salt (8.40 g, 26 mmol, theoretical) in anhydrous dimethylformamide (200 mL) under nitrogen, Hünig’s base (6.70 g, 9.04 mL, 51.9 mmol) was added, followed by 2,5-dioxopyrrolidin-1-yl *(tert*-butoxycarbonyl)-*L*-phenylalaninate (6.27 g, 17.3 mmol), and the mixture was stirred overnight at room temperature. The reaction was quenched with 1 M hydrochloric acid (100 mL) and extracted with ethyl acetate (3 × 150 mL). The combined organic layers were washed with water (3 × 200 mL), satd. brine (300 mL), dried (MgSO_4_) and concentrated to give (*S*)-5-acetoxy-2-((*S*)-2-((*tert*-butoxycarbonyl)amino)-3-phenylpropanamido)pentanoic acid (7.57 g) as a white foam. ^1^H NMR (300 MHz, DMSO-*d*_6_): δ 8.13 (d, 1 H, *J* = 6.9 Hz, NH), 7.33–7.23 (m, 5 H, 5 × ArH), 6.90 (d, 1 H, *J* = 8.7 Hz, NH), 4.30–4.12 (m, 2 H, 2 × α-CH), 4.04–3.94 (m, 2 H, CH_2_O), 2.97 (dd, 1 H, *J* = 13.7, 3.8 Hz, 0.5 × benzylic CH_2_), 2.73 (dd, 1 H, *J* = 13.5, 10.8 Hz, 0.5 × benzylic CH_2_), 2.00 (s, 3 H, CH_3_), 1.86–1.74 (m, 1 H, 0.5 × CH_2_), 1.70–1.57 (m, 3 H, 1.5 × CH_2_), 1.29 (s, 9 H, Boc). ^13^C{^1^H} NMR (75 MHz, DMSO-*d*_6_): δ 173.3, 171.9, 170.4, 155.2, 138.2, 129.2, 128.0, 126.2, 78.0, 63.4, 55.6, 51.4, 37.3, 28.1, 27.8, 24.6, 20.7. LC-MS (ESI positive, *m*/*z*) calcd. for C_21_H_30_N_2_O_7_ [M + H]^+^: 423.21, found: 423.20.

#### 3.2.4. (S)-2-((S)-2-((tert-Butoxycarbonyl)amino)-3-phenylpropanamido)-5-hydroxypentanoic Acid (**9**)

To a solution of (*S*)-5-acetoxy-2-((*S*)-2-((*tert*-butoxycarbonyl)amino)-3-phenylpropanamido)pentanoic acid (7.57 g, 17.9 mmol) in methanol (102 mL), 1 M aqueous sodium hydroxide solution (44.9 mL, 44.9 mmol) was added, and the mixture was stirred for 3 h at room temperature. The methanol was removed under vacuum, and the aqueous mixture was acidified to pH 2 with 1 M hydrochloric acid and extracted with ethyl acetate (3 × 50 mL). The combined organic layers were washed with satd. brine (100 mL), dried (MgSO_4_) and concentrated to give (*S*)-2-((*S*)-2-((*tert*-butoxycarbonyl)amino)-3-phenylpropanamido)-5-hydroxypentanoic acid (6.76 g, 68% over 3 steps) as a white foam. ^1^H NMR (300 MHz, DMSO-*d*_6_): δ 8.09 (d, 1 H, *J* = 7.8 Hz, NH), 7.35–7.13 (m, 5 H, 5 × ArH), 6.88 (d, 1 H, *J* = 8.7 Hz, NH), 4.28–4.13 (m, 2 H, 2 × α-CH), 3.40 (t, 2 H, *J* = 6.2 Hz, CH_2_O), 2.98 (dd, 1 H, *J* = 13.8, 3.6 Hz, 0.5 × benzylic CH_2_), 2.78–2.64 (m, 1 H, 0.5 × benzylic CH_2_), 1.85–1.71 (m, 1 H, 0.5 × CH_2_), 1.71–1.57 (m, 1 H, 0.5 × CH_2_), 1.54–1.42 (m, 2 H, CH_2_), 1.29 (s, 9 H, Boc). ^13^C{^1^H} NMR (75 MHz, DMSO-*d*_6_): δ 173.6, 171.8, 155.2, 138.2, 129.2, 128.0, 126.2, 78.0, 60.2, 55.6, 51.8, 47.4, 37.5, 28.7, 28.2, 28.0, 24.6, 20.7. LC-MS (ESI positive, *m*/*z*) calcd. for C_19_H_28_N_2_O_6_ [M + H]^+^: 381.20, found: 381.15.

#### 3.2.5. Tert-Butyl ((S)-1-oxo-1-(((S)-2-oxotetrahydro-2H-pyran-3-yl)amino)-3-phenylpropan-2-yl) carbamate (**10**)

To a solution of (*S*)-2-((*S*)-2-((*tert*-butoxycarbonyl)amino)-3-phenylpropanamido)-5-hydroxypentanoic acid (8.62 g, 22.7 mmol) in anhydrous dichloromethane (90 mL) under nitrogen, EDCI hydrochloride (4.78 g, 24.9 mmol) was added, followed by Hünig’s base (8.79 g, 11.8 mL, 67.9 mmol), and the mixture was stirred for 2 h at room temperature. The reaction was quenched with 1 M hydrochloric acid (200 mL) and extracted with dichloromethane (3 × 100 mL). The combined organic layers were washed with water (150 mL), satd. aqueous sodium bicarbonate (150 mL), satd. brine (300 mL), dried (MgSO_4_) and concentrated to give *tert*-butyl ((*S*)-1-oxo-1-(((*S*)-2-oxotetrahydro-2*H*-pyran-3-yl)amino)-3-phenylpropan-2-yl) carbamate (6.94 g, 86%) as a white solid. TLC: R*_f_* = 0.45 (ethyl acetate—dichloromethane, 3:7 *v*/*v*). ^1^H NMR (300 MHz, DMSO-*d*_6_): δ 8.29 (m, 1 H, NH), 7.24 (m, 5 H, 5 × ArH), 6.90 (d, 1 H, *J* = 8.7 Hz, NH), 4.66–4.54 (m, 1 H, lactone α-CH), 4.46–4.33 (m, 1 H, 0.5 × CH_2_O), 4.28–4.15 (m, 2 H, α-CH and 0.5 × CH_2_O), 3.08–2.91 (m, 1 H, 0.5 × benzylic CH_2_), 2.81–2.68 (m, 1 H, 0.5 × benzylic CH_2_), 2.20–2.04 (m, 1 H, 0.5 × CH_2_), 1.97–1.80 (m, 2 H, CH_2_), 1.79–1.60 (m, 1 H, 0.5 × CH_2_), 1.29 (s, 9 H, Boc). ^13^C{^1^H} NMR (75 MHz, DMSO-*d*_6_): δ 171.6, 171.5, 171.3, 155.2, 138.0, 129.2, 128.0, 126.2, 78.0, 67.7, 59.8, 55.6, 37.4, 28.1, 25.0, 24.7, 21.2, 21.1, 14.1 (additional resonances are due to rotamers). LC-MS (ESI positive, *m*/*z*) calcd. for C_19_H_26_N_2_O_5_ [M + H]^+^: 363.19, found: 363.15.

#### 3.2.6. (3S,6S)-3-Benzyl-6-(3-hydroxypropyl)piperazine-2,5-dione (**11**)

A solution of *tert*-butyl ((*S*)-1-oxo-1-(((*S*)-2-oxotetrahydro-2*H*-pyran-3-yl)amino)-3-phenylpropan-2-yl) carbamate (4.90 g, 13.5 mmol) in trifluoroacetic acid: anhydrous dichloromethane (1:1, 90 mL) was stirred for 45 min at room temperature. The solution was subjected to azeotropic distillation with diethyl ether and dichloromethane to give (*S*)-2-amino-*N*-((*S*)-2-oxotetrahydro-2*H*-pyran-3-yl)-3-phenylpropanamide trifluoroacetic acid salt that was used without further purification.

To a solution of (*S*)-2-amino-*N*-((*S*)-2-oxotetrahydro-2*H*-pyran-3-yl)-3-phenylpropanamide trifluoroacetic acid salt (13.5 mmol) in anhydrous dimethylformamide (80 mL) under nitrogen, Hünig’s base (3.49 g, 4.71 mL, 27.1 mmol) was added, and the mixture was stirred overnight at room temperature. The suspension was diluted with diethyl ether (200 mL) and the resulting solid was collected by Büchner filtration to give (3*S*,6*S*)-3-benzyl-6-(3-hydroxypropyl)piperazine-2,5-dione (2.66 g, 75%) as a white solid. ^1^H NMR (300 MHz, DMSO-*d*_6_): δ 8.10 (br s, 1 H, NH), 8.03 (br s, 1 H, NH), 7.28–7.12 (m, 5 H, 5 × ArH), 4.26 (t, 1 H, *J* = 5.1 Hz, OH), 4.18–4.14 (m, 1 H, α-CH), 3.58–3.54 (m, 1 H, α-CH), 3.14–3.09 (m, 3 H, CH_2_O and 0.5 × benzylic CH_2_), 2.85 (dd, 1 H, *J* = 13.5, 4.9 Hz, 0.5 × benzylic CH_2_), 1.20–1.09 (m, 1 H, 0.5 × CH_2_), 0.99–0.90 (m, 2 H, CH_2_), 0.70–0.60 (m, 1 H, 0.5 × CH_2_). ^13^C{^1^H} NMR (75 MHz, DMSO-*d*_6_): δ 167.2, 166.2, 136.2, 130.3, 128.0, 126.6, 60.4, 55.4, 53.9, 38.3, 30.4, 27.4. LC-MS (ESI positive, *m*/*z*) calcd. for C_14_H_18_N_2_O_3_ [M + H]^+^: 263.14, found: 263.10.

#### 3.2.7. (3S,8aS)-3-Benzyl-6-hydroxyhexahydropyrrolo[1,2-a]pyrazine-1,4-dione (**12**)

To a solution of (3*S*,6*S*)-3-benzyl-6-(3-hydroxypropyl)piperazine-2,5-dione (2.66 g, 10.2 mmol) in anhydrous DMSO (50 mL), 2-iodoxybenzoic acid (containing stabiliser, 30%, IBX) (10.9 g, 11.7 mmol) was added, and the mixture was stirred at room temperature overnight. The suspension was diluted with water (100 mL) and stirred for 5 min. The solid was removed by Büchner filtration and discarded. The filtrate was concentrated under vacuum to ~50 mL and the residual material was purified using automated chromatography under reversed-phase conditions (gradient of 0 → 100% acetonitrile in water). The fractions containing the product were concentrated under vacuum to remove acetonitrile. The aqueous mixture was basified to pH 8–9 with satd. aqueous sodium bicarbonate and extracted with ethyl acetate (6 × 100 mL). The combined organic layers were washed with water (125 mL), satd. brine (125 mL), dried (MgSO_4_) and concentrated to give (3*S*,8a*S*)-3-benzyl-6-hydroxyhexahydropyrrolo[1,2-*a*]pyrazine-1,4-dione (1.77 g, 68%) as a 7 to 3 mixture of diastereomers. ^1^H NMR (300 MHz, DMSO-*d*_6_): δ 8.04 (br s, 0.7 H, 0.7 × NH), 7.87 (br s, 0.3 H, 0.3 × NH), 7.39–7.15 (m, 5 H, 5 × ArH), 6.06 (d, 1 H, *J* = 4.2 Hz, OH), 5.52–5.45 (m, 0.7 H, 0.7 × CHNO), 5.45–5.39 (m, 0.3 H, 0.3 × CHNO), 4.39–4.12 (m, 2 H, 2 × α-CH), 3.25–2.87 (m, 2 H, benzylic CH_2_), 2.11–1.41 (m, 4 H, 2 × CH_2_). ^13^C{^1^H} NMR (75 MHz, DMSO-*d*_6_): δ 170.7, 169.0, 166.7, 165.3, 138.0, 137.2, 129.8, 129.5, 129.3, 128.6, 128.1, 126.4, 126.2, 79.7, 78.5, 58.7, 56.7, 55.8, 55.6, 35.4, 33.8, 32.7, 31.6, 25.5, 24.2. LC-MS (ESI positive, *m*/*z*) calcd. for C_14_H_16_N_2_O_3_ [M + H]^+^: 261.12, found: 261.10.

#### 3.2.8. (3S,8aS)-3-Benzyl-6-methoxyhexahydropyrrolo[1,2-a]pyrazine-1,4-dione (**Fragment A, 13**)

To a solution of (3*S*,8a*S*)-3-benzyl-6-hydroxyhexahydropyrrolo[1,2-*a*]pyrazine-1,4-dione (1.36 g, 5.23 mmol) in anhydrous methanol (25 mL) under nitrogen, *p*-toluenesulfonic acid (30.0 mg, 0.16 mmol) was added, and the mixture was stirred at room temperature for 1.5 h. The reaction was concentrated and the material was partitioned between satd. aqueous sodium bicarbonate (20 mL) and ethyl acetate (20 mL). The layers were separated and the aqueous layer was extracted with ethyl acetate (2 × 20 mL). The combined organic layers were washed with satd. brine (30 mL), dried (MgSO_4_) and concentrated to give (3*S*,8a*S*)-3-benzyl-6-methoxyhexahydropyrrolo[1,2-*a*]pyrazine-1,4-dione (1.22 g, 85%) as a white foam. ^1^H NMR (300 MHz, DMSO-*d*_6_): δ 8.11 (br s, 0.7 H, 0.7 × NH), 7.96 (br s, 0.3 H, 0.3 × NH), 7.38–7.18 (m, 5 H, 5 × ArH), 5.28 (t, 0.7 H, *J* = 3.5 Hz, 0.7 × CHNO), 5.16 (d, 0.3 H, *J* = 3.5 Hz, 0.3 × CHNO), 4.45 (t, 0.7 H, *J* = 5.2 Hz, 0.7 × α-CH), 4.36 (t, 0.3 H, *J* = 5.6 Hz, 0.3 × α-CH), 4.21–4.13 (m, 1 H, α-CH), 3.26 (s, 2.1 H. 2.1 × CH_3_), 3.22–2.92 (m, 2.9 H, 0.9 × CH_3_ and benzylic CH_2_), 2.06–1.80 (m, 2 H, CH_2_), 1.67–1.46 (m, 2 H, CH_2_). ^13^C{^1^H} NMR (75 MHz, DMSO-*d*_6_): δ 170.6, 168.6, 168.1, 166.5, 137.7, 137.0, 129.7, 129.6, 128.1, 128.0, 126.5, 126.3, 87.4, 86.3, 58.9, 56.9, 56.3, 56.1, 55.7, 55.5, 35.6, 33.8, 30.6, 29.6, 25.2, 24.00. LC-MS (ESI positive, *m*/*z*) calcd. for C_15_H_18_N_2_O_3_ [M + H + CH_3_CN]^+^: 316.17, found: 316.15.

#### 3.2.9. Rac.-Diethyl 2-(benzylidyne-λ^6^-oxidaneyl)-2-methylmalonate (**15**)

To a suspension of sodium hydride (60% disp. in oil, 1.39 g, 34.8 mmol) in anhydrous toluene (176 mL) in an oven-dried flask under nitrogen, benzyl alcohol (3.24 g, 3.28 mL, 31.6 mmol) was added, and the suspension was stirred for 1 h at room temperature. Diethyl 2-bromo-2-methylmalonate (8.00 g, 6.04 mL, 31.6 mmol) was added dropwise and the mixture was heated at 110 °C for 2 h. The reaction mixture was cooled to room temperature and quenched with 1 M hydrochloric acid (150 mL) and extracted with diethyl ether (3 × 80 mL). The combined organic layers were washed with satd. aqueous sodium bicarbonate (150 mL), satd. brine (150 mL), dried (MgSO_4_) and concentrated to give a yellow oil. This material was purified using automated chromatography under normal phase conditions (gradient of 0 → 100% diethyl ether in petrol) to give rac.-diethyl 2-(benzylidyne-λ^6^-oxidaneyl)-2-methylmalonate (7.58 g) as a colourless oil. This material was used without further purification.

#### 3.2.10. Rac.-2-(Benzylidyne-λ^6^-oxidaneyl)-3-ethoxy-2-methyl-3-oxopropanoic Acid (**16a/16b**)

To a solution of diethyl 2-(benzylidyne-λ^6^-oxidaneyl)-2-methylmalonate (7.58 g, 27.1 mmol) in ethanol (142 mL), potassium hydroxide (1.82 g, 32.5 mmol) was added, and the mixture was stirred for 2 h at room temperature. The suspension was filtered through Celite, and the filtrate was concentrated under vacuum to give a yellow oil. This material was partitioned between satd. aqueous sodium bicarbonate (150 mL) and dichloromethane (150 mL) and the layers were separated. The aqueous layer was washed with dichloromethane (3 × 80 mL) and the organic layers were discarded. The aqueous layer was acidified to pH 1–2 with 6 M hydrochloric acid and extracted with dichloromethane (3 × 80 mL). The combined organic layers were washed with 1 M hydrochloric acid (100 mL), water (100 mL), satd. brine (150 mL), dried (MgSO_4_) and concentrated to give an oil. This material was purified using automated chromatography under reversed-phase conditions (gradient of 0 → 100% acetonitrile in water) to give rac.-2-(benzylidyne-λ^6^-oxidaneyl)-3-ethoxy-2-methyl-3-oxopropanoic acid (3.76 g, 47% over 2 steps) as a colourless oil.

#### 3.2.11. (R)-2-(Benzylidyne- λ^6^-oxidaneyl)-3-ethoxy-2-methyl-3-oxopropanoic Acid (**16b, Fragment B**)

To a solution of rac.-2-(benzylidyne-λ^6^-oxidaneyl)-3-ethoxy-2-methyl-3-oxopropanoic acid (3.18 g, 12.6 mmol) in dimethyl carbonate (90 mL), cinchonidine (3.71 g, 12.6 mmol) was added, and the suspension was stirred for 15 min at room temperature. The suspension was warmed until a clear solution was obtained, which was allowed to cool to room temperature. The resulting solid was collected by suction filtration (6.17 g). The filtrate was concentrated to give an oily solid. This material was partitioned between 1 M hydrochloric acid (20 mL) and ethyl acetate (20 mL). The layers were separated, and the aqueous layer was extracted with ethyl acetate (2 × 20 mL). The combined organic layers were washed with satd. brine (30 mL), dried (MgSO_4_) and concentrated under reduced pressure to give (*R*)-2-(benzylidyne- λ^6^-oxidaneyl)-3-ethoxy-2-methyl-3-oxopropanoic acid (1.06 g, 75.2% ee) as an oil. The solid collected above (6.17 g) was suspended in dimethyl carbonate (120 mL) and warmed until a clear solution was obtained, which was allowed to warm to room temperature. The resulting solid was collected by suction filtration (2.7 g). The filtrate was concentrated under vacuum to give an oily solid. This material was partitioned between 1 M hydrochloric acid (40 mL) and ethyl acetate (40 mL). The layers were separated and the aqueous layer was extracted with ethyl acetate (2 × 40 mL). The combined organic layers were washed with satd. brine (80 mL), dried (MgSO_4_) and concentrated to give (*R*)-2-(benzylidyne-λ^6^-oxidaneyl)-3-ethoxy-2-methyl-3-oxopropanoic acid (0.87 g, 66.6% ee) as an oil. ^1^H NMR (300 MHz, CDCl_3_): δ 8.25 (br s, COOH), 7.38 (m, 5 H, 5 × ArH), 4.63 (s, 2 H, benzylic CH_2_), 4.28 (q, 2 H, *J* = 7.1 Hz, C*H*_2_CH_3_), 1.78 (s, 3 H, CH_3_), 1.31 (t, 3 H, *J* = 7.1 Hz, CH_2_C*H*_3_). ^13^C{^1^H} NMR (75 MHz, CDCl_3_): δ 172.3, 168.8, 136.9, 128.6, 128.3, 128.2, 81.9, 68.7, 62.7, 20.6, 14.1. LC-MS (ESI negative, *m*/*z*) calcd. for C_13_H_16_O_5_ [M − H]^−^: 251.09, found: 251.05. Enantiomeric excesses were measured with a Phenomenex Lux-Cellulose 2 column (250 × 4.6 mm, 5 µm) eluting with hexane:ethanol:diethylamine (93:7:0.1 *v*/*v*/*v*).

#### 3.2.12. Ethyl (R)- and (S)-3-((3S,6R,8aS)-3-benzyl-6-methoxy-1,4-dioxohexahydropyrrolo[1,2-a]pyrazin-2(1H)-yl)-2-(benzyloxy)-2-methyl-3-oxopropanoate (major isomer: **18**)

To an ice-cold solution of (*R*)-2-(benzylidyne-λ^6^-oxidaneyl)-3-ethoxy-2-methyl-3-oxopropanoic acid (1.87 g, 7.42 mmol) and anhydrous dimethylformamide (0.58 g, 0.62 mL, 7.99 mmol) in an oven -dried flask under nitrogen in anhydrous dichloromethane (26 mL), thionyl chloride (1.76 g, 1.10 mL, 14.8 mmol) was added, and the mixture was heated at 41 °C for 2.5 h. The reaction was cooled to room temperature and concentrated under high vacuum to give a yellow oil. A solution of this material in anhydrous dichloromethane (10 mL) was added to a −30 °C solution of (3*S*,8a*S*)-3-benzyl-6-methoxyhexahydropyrrolo[1,2-*a*]pyrazine-1,4-dione (1.41 g, 5.15 mmol) in an oven-dried flask under nitrogen in anhydrous pyridine:anhydrous dichloromethane (1:1, 20 mL), and the mixture was allowed to warm slowly to room temperature and stirred overnight. The orange suspension was cooled to 0 °C and the reaction was quenched with 1 M hydrochloric acid (20 mL) and diluted with ethyl acetate (20 mL). The layers were separated, and the aqueous layer was extracted with ethyl acetate (2 × 30 mL). The combined organic extracts were washed with 1 M hydrochloric acid (40 mL), water (40 mL), satd. aqueous sodium bicarbonate (40 mL), satd. brine (40 mL), dried (MgSO_4_) and concentrated to give an orange oily foam. This material was purified using automated chromatography under normal phase conditions (gradient of 0 → 100% ethyl acetate in petrol) to give ethyl (*R*)- and (*S*)-3-((3*S*,6*R*,8a*S*)-3-benzyl-6-methoxy-1,4-dioxohexahydropyrrolo[1,2-*a*]pyrazin-2(1*H*)-yl)-2-(benzyloxy)-2-methyl-3-oxopropanoate (1.75 g, 67%) in an 8 to 2 ratio. ^1^H NMR (400 MHz, DMSO-*d*_6_): Major isomer—δ 7.30–7.20 (m, 8 H, 8 × ArH), 6.96–6.90 (m, 2 H, 2 × ArH), 5.35–5.30 (m, 1 H, CHNO), 4.93 (t, 1 H, *J* = 3.8 Hz, α-CH), 4.70 (d, 1 H, *J* = 10.9 Hz, 0.5 × benzylic CH_2_), 4.32 (d, 1 H, *J* = 10.9 Hz, 0.5 × benzylic CH_2_), 4.29–4.17 (m, 2 H, CH_2_), 3.70–3.64 (m, 1 H, α-CH), 3.30 (s, 3 H. OCH_3_), 3.27–3.23 (m, 2 H, benzylic CH_2_), 1.75 (s, 3 H, CH_3_), 1.69–1.52 (m, 3 H, 0.5 × CH_2_ and CH_2_), 1.39–1.22 (m, 4 H, 0.5 × CH_2_ and CH_3_). ^13^C{^1^H} NMR (100 MHz, DMSO-*d*_6_): δ 171.0, 168.7, 168.1, 164.0, 137.2, 134.3, 130.7, 128.4, 128.2, 181.1, 127.9, 127.5, 87.9, 82.7, 66.8, 61.1, 59.4, 57.7, 55.9, 37.9, 28.7, 26.3, 20.5, 13.9 (not all carbon resonances were detected). TLC: R*_f_* = 0.55 (ethyl acetate—petrol, 4:6 *v*/*v*). LC-MS (ESI positive, *m*/*z*) calcd. for C_28_H_32_N_2_O_7_ [M + Na]^+^: 531.21, found: 531.20.

#### 3.2.13. Ethyl (2R,5S,10aS,10bS)-5-benzyl-10b-hydroxy-8-methoxy-2-methyl-3,6-dioxooctahydro-8H-oxazolo[3,2-a]pyrrolo[2,1-c]pyrazine-2-carboxylate (**19**)

To a suspension of Pd on C (10%) (0.70 g, 40% weight) in tetrahydrofuran:methanol (1:10, 24 mL) was added a solution of ethyl 3-((3*S*,8a*S*)-3-benzyl-6-methoxy-1,4-dioxohexahydropyrrolo[1,2-*a*]pyrazin-2(1*H*)-yl)-2-(benzyloxy)-2-methyl-3-oxopropanoate (1.75 g, 3.44 mmol) in methanol (5.6 mL) and the mixture was evacuated and filled with hydrogen (via balloon). This procedure was repeated three times, and the mixture was stirred overnight at room temperature. The suspension was filtered through Celite, and the filtrate was concentrated to give a mixture of product and starting material. This material was purified using automated chromatography under normal phase conditions (gradient of 0 → 100% ethyl acetate in petrol) to give ethyl (2*R*,5*S*,10a*S*,10b*S*)-5-benzyl-10b-hydroxy-8-methoxy-2-methyl-3,6-dioxooctahydro-8*H*-oxazolo[3,2-*a*]pyrrolo[2,1-*c*]pyrazine-2-carboxylate (490 mg) and starting material (0.59 g).

To a suspension of Pd on C (10%) (236 mg, 40% weight) in tetrahydrofuran:methanol (1:10, 9 mL) was added a solution of ethyl 3-((3*S*,8a*S*)-3-benzyl-6-methoxy-1,4-dioxohexahydropyrrolo[1,2-*a*]pyrazin-2(1*H*)-yl)-2-(benzyloxy)-2-methyl-3-oxopropanoate (0.59 g, 1.16 mmol) in methanol (5.6 mL) and the mixture was evacuated and filled with hydrogen (via balloon). This procedure was repeated three times, and the mixture was stirred overnight at room temperature. The suspension was filtered through Celite, and the filtrate was concentrated to give a white foam. This material was purified using automated chromatography under normal phase conditions (gradient of 0 → 100% ethyl acetate in petrol) to give ethyl (2*R*,5*S*,10a*S*,10b*S*)-5-benzyl-10b-hydroxy-8-methoxy-2-methyl-3,6-dioxooctahydro-8*H*-oxazolo[3,2-*a*]pyrrolo[2,1-*c*]pyrazine-2-carboxylate (366 mg). This material was combined with the first purification to give ethyl (2*R*,5*S*,10a*S*,10b*S*)-5-benzyl-10b-hydroxy-8-methoxy-2-methyl-3,6-dioxooctahydro-8*H*-oxazolo[3,2-*a*]pyrrolo[2,1-*c*]pyrazine-2-carboxylate (0.86 g, 59%) as a single isomer. TLC: R*_f_* = 0.55 (ethyl acetate—petrol, 45:55 *v*/*v*). ^1^H NMR (300 MHz, DMSO-*d*_6_): δ 7.43 (br s, 1 H, OH), 7.31–7.11 (m, 5 H, 5 × ArH), 5.48–5.45 (m, 1 H, CHNO), 4.58 (dd, 1 H, *J* = 8.1, 5.0 Hz, α-CH), 4.18 (q, 2 H, *J* = 7.1 Hz, C*H*_2_CH_3_), 3.88 (dd, 1 H, *J* = 8.7, 4.1 Hz, α-CH), 3.35–3.21 (m, 4 H, OMe and 0.5 × benzylic CH_2_), 2.88 (dd, 1 H, *J* = 13.6, 8.1 Hz, 0.5 × benzylic CH_2_), 2.19–2.00 (m, 2 H, CH_2_), 1.80–1.68 (m, 2 H, CH_2_), 1.46 (s, 3 H, CH_3_), 1.22 (t, 3 H, *J* = 7.1 Hz, CH_2_C*H*_3_). ^13^C{^1^H} NMR (75 MHz, DMSO-*d*_6_): δ 168.1, 165.4, 165.2, 138.0, 129.7, 127.8, 126.1, 104.4, 88.3, 82.0, 62.9, 61.9, 55.9, 55.4, 38.4, 29.6, 23.5, 20.6, 13.9. LC-MS (ESI positive, *m*/*z*) calcd. for C_21_H_26_N_2_O_7_ [M + Na]^+^: 441.16, found: 441.15.

#### 3.2.14. (2R,5S,8R,10aS,10bS)-5-benzyl-10b-hydroxy-8-methoxy-2-methyl-3,6-dioxooctahydro-8H-oxazolo[3,2-a]pyrrolo[2,1-c]pyrazine-2-carboxylic Acid (**20**)

To a solution of ethyl (2*R*,5*S*,10a*S*,10b*S*)-5-benzyl-10b-hydroxy-8-methoxy-2-methyl-3,6-dioxooctahydro-8*H*-oxazolo[3,2-*a*]pyrrolo[2,1-*c*]pyrazine-2-carboxylate (0.85 g, 2.03 mmol) in tetrahydrofuran (5.5 mL), 1 M aqueous sodium hydroxide solution (5.08 mL, 5.08 mmol) was added, and the mixture was stirred at room temperature for 2 h. The solution was cooled to 0 °C and quenched with 1 M hydrochloric acid (10 mL, to pH 1–2) and extracted with ethyl acetate (3 × 20 mL). The combined organic extracts were washed with satd. brine (30 mL), dried (MgSO_4_) and concentrated to give (2*R*,5*S*,10a*S*,10b*S*)-5-benzyl-10b-hydroxy-8-methoxy-2-methyl-3,6-dioxooctahydro-8*H*-oxazolo [3,2-*a*]pyrrolo[2,1-*c*]pyrazine-2-carboxylic acid (0.80 g, 99%) as a white foam. TLC: R*_f_* = 0.55 (ethyl acetate—petrol, 4:6 *v*/*v*). ^1^H NMR (300 MHz, DMSO-*d*_6_): δ 7.31–7.10 (m, 5 H, 5 × ArH), 5.44–5.40 (m, 1 H, CHNO), 4.57 (dd, 1 H, *J* = 6.8, 4.0 Hz, α-CH), 3.84 (dd, 1 H, *J* = 7.9, 4.8 Hz, α-CH), 3.21 (s, 3 H, OMe), 3.15 (dd, 1 H, *J* = 13.7, 6.9 Hz, 0.5 × benzylic CH_2_), 3.00 (dd, 1 H, *J* = 13.7, 3.9 Hz, 0.5 × benzylic CH_2_), 2.13–2.01 (m, 2 H, CH_2_), 1.77–1.66 (m, 2 H, CH_2_), 1.39 (s, 3 H, CH_3_). ^13^C{^1^H} NMR (75 MHz, DMSO-*d*_6_): δ 170.7, 169.0, 166.3, 138.7, 129.8, 127.8, 125.9, 104.3, 88.0, 85.1, 62.0, 55.3, 37.5, 29.6, 23.6, 20.0. LC-MS (ESI negative, *m*/*z*) calcd. for C_19_H_22_N_2_O_7_ [M − H]^−^: 389.14, found: 389.10.

#### 3.2.15. (2R,5S,10aS,10bS)-5-Benzyl-8,10b-dihydroxy-2-methyl-3,6-dioxooctahydro-8H-oxazolo[3,2-a]pyrrolo[2,1-c]pyrazine-2-carbonyl Azide (**21**)

To an ice-cold solution of (2*R*,5*S*,8*R*,10a*S*,10b*S*)-5-benzyl-10b-hydroxy-8-methoxy-2-methyl-3,6-dioxooctahydro-8*H*-oxazolo[3,2-*a*]pyrrolo[2,1-*c*]pyrazine-2-carboxylic acid (100 mg, 0.25 mmol) and anhydrous dimethylformamide (3.7 mg, 4 µL, 0.05 mmol) in anhydrous dichloromethane (2 mL) under nitrogen, oxalyl chloride (84 mg, 57 µL, 0.51 mmol) was added, and the mixture was stirred for 2 h at room temperature and subsequently concentrated under high vacuum. This material was dissolved in acetone (2 mL) and cooled to −78 °C. A solution of *tetra*-butylammonium azide (78 mg, 0.28 mmol) in acetone (1 mL) was added, and the mixture was stirred at −78 °C for 1 h followed by 0 °C for 15 min. The reaction was quenched with water (10 mL) and extracted with ethyl acetate (3 × 10 mL). The combined organic extracts were washed with satd. aqueous sodium bicarbonate (20 mL), satd. brine (20 mL), dried (MgSO_4_) and concentrated to give an off-white foam. This material was purified using automated chromatography under normal phase conditions (gradient of 0 → 100% ethyl acetate in dichloromethane) to give (2*R*,5*S*,10aS,10b*S*)-5-benzyl-8,10b-dihydroxy-2-methyl-3,6-dioxooctahydro-8*H*-oxazolo[3,2-*a*]pyrrolo[2,1-*c*]pyrazine-2-carbonyl azide (52 mg, 52%) as an impure 8 to 2 mixture of diastereomers. TLC: R*_f_* = 0.45 (ethyl acetate—dichloromethane, 3:7 *v*/*v*). ^1^H NMR (300 MHz, CDCl_3_): δ 7.26–6.98 (m, 5 H, 5 × ArH), 5.65–5.52 (m, 1 H, CHNO), 4.67–4.58 (m, 1 H, α-CH), 4.04 (br s, 1H, OH), 3.79–3.62 (m, 1 H, α-CH), 3.34–3.13 (m, 2 H, benzylic CH_2_), 2.28–2.05 (m, 2 H, CH_2_), 1.81–1.53 (m, 2 H, CH_2_), 1.41 (s, 3 H, CH_3_). Minor LC-MS (ESI positive, *m*/*z*) calcd. for C_18_H_19_N_5_O_6_ [M + Na]^+^: 424.12, found: 424.10. Major LC-MS (ESI positive, *m*/*z*) calcd. for C_18_H_19_N_5_O_6_ [M + Na]^+^: 424.12, found: 424.15.

#### 3.2.16. Benzyl ((2R,5S,10aS,10bS)-5-benzyl-10b-hydroxy-8-methoxy-2-methyl-3,6-dioxooctahydro-8H-oxazolo[3,2-a]pyrrolo[2,1-c]pyrazin-2-yl)carbamate (**22**)

A solution of (2*R*,5*S*,10aS,10b*S*)-5-benzyl-8,10b-dihydroxy-2-methyl-3,6-dioxooctahydro-8*H*-oxazolo[3,2-*a*]pyrrolo[2,1-*c*]pyrazine-2-carbonyl azide (287 mg, 0.72 mmol) in benzyl alcohol (4 mL) under nitrogen was heated at 130 °C for 3 min, and the benzyl alcohol was removed by Kugelrohr distillation at 50 °C to give a yellow oil. This material was purified using automated chromatography under normal phase conditions (gradient of 0 → 100% ethyl acetate in petrol) to give benzyl ((2*R*,5*S*,10a*S*,10b*S*)-5-benzyl-10b-hydroxy-8-methoxy-2-methyl-3,6-dioxooctahydro-8*H*-oxazolo[3,2-*a*]pyrrolo[2,1-*c*] pyrazin-2-yl)carbamate (240 mg, 70%) as a mixture of diastereomers.

To a solution of benzyl ((2*R*,5*S*,10a*S*,10b*S*)-5-benzyl-10b-hydroxy-8-methoxy-2-methyl-3,6-dioxooctahydro-8*H*-oxazolo[3,2-*a*]pyrrolo[2,1-*c*] pyrazin-2-yl)carbamate (240 mg, 0.59 mmol) in anhydrous methanol (6.5 mL), *p*-toluenesulfonic acid (3.20 mg, 18.0 µmol) was added, and the mixture was stirred at room temperature for 45 min. The reaction was quenched with sat. aqueous sodium bicarbonate (20 mL) and extracted with ethyl acetate (3 × 20 mL). The combined organic extracts were washed with satd. aqueous sodium bicarbonate (20 mL), satd. brine (30 mL), dried (MgSO_4_) and concentrated to give benzyl ((2*R*,5*S*,10a*S*,10b*S*)-5-benzyl-10b-hydroxy-8-methoxy-2-methyl-3,6-dioxooctahydro-8*H*-oxazolo[3,2-*a*]pyrrolo[2,1-*c*]pyrazin-2-yl)carbamate (223 mg, 90%) as a mixture of diastereomers.

*Major diastereomer:*

TLC: R*_f_* = 0.55 (ethyl acetate—petrol, 4:6 *v*/*v*). ^1^H NMR (300 MHz, CD_3_CN): δ 7.46–7.16 (m, 10 H, 10 × ArH), 6.95 (br s, 1 H, carbamate NH), 5.66–5.62 (m, 1 H, OH), 5.55–5.49 (m, 1 H, CHNO), 5.13 (s, 2 H, carbamate benzylic CH_2_), 4.69–4.62 (m, 1 H, α-CH), 3.88–3.79 (m, 1 H, α-CH), 3.39–3.15 (m, 5 H, benzylic CH_2_ and OMe), 2.19–2.08 (m, 2 H, CH_2_), 1.81–1.70 (m, 2 H, CH_2_), 1.49 (s, 3 H, CH_3_). ^13^C{^1^H} NMR (75 MHz, CD_3_CN): δ 166.6, 166.6, 156.5, 139.4, 136.7, 130.7, 129.1, 128.9, 128.7, 128.5, 126.8, 103.5, 89.2, 87.6, 67.7, 62.8, 57.1, 55.9, 38.8, 30.3, 24.1, 23.9. LC-MS (ESI positive, *m*/*z*) calcd. for C_26_H_29_N_3_O_7_ [M + Na]^+^: 518.19, found: 518.15.

*Minor diastereomer:*

TLC: R*_f_* = 0.2 (ethyl acetate—petrol, 4:6 *v*/*v*). ^1^H NMR (300 MHz, CD_3_CN): δ 7.38–7.12 (m, 10 H, 10 × ArH), 6.88 (br s, 1 H, carbamate NH), 5.69 (br s, 1 H, OH), 5.19–5.15 (m, 1 H, CHNO), 5.11 (s, 2 H, carbamate benzylic CH_2_), 4.52 (t, 1 H, *J* = 5.6 Hz α-CH), 3.57–3.47 (m, 1 H, α-CH), 3.34–3.18 (m, 5 H, benzylic CH_2_ and OMe), 2.01–1.59 (m, 4 H, 2 × CH_2_), 1.39 (s, 3 H, CH_3_). ^13^C{^1^H} NMR (75 MHz, CD_3_CN): δ 167.4, 166.9, 156.9, 139.1, 137.2, 131.4, 129.6, 129.3, 129.2, 128.8, 127.3, 104.1, 88.7, 87.8, 68.1, 65.7, 58.6, 57.0, 39.9, 30.9, 30.7, 24.5, 24.3. LC-MS (ESI positive, *m*/*z*) calcd. for C_26_H_29_N_3_O_7_ [M + Na]^+^: 518.19, found: 518.15.

#### 3.2.17. (6aR,9R,10aR)-N-((2R,5S,10aS,10bS)-5-Benzyl-10b-hydroxy-8-methoxy-2-methyl-3,6-dioxo octahydro-8H-oxazolo[3,2-a]pyrrolo[2,1-c]pyrazin-2-yl)-7-methyl-4,6,6a,7,8,9,10,10a-octa hydroindolo[4,3-fg]quinoline-9-carboxamide Formate Salt (**31**)

To a suspension of Pd on C (10%) (0.70 g, 40% weight) in tetrahydrofuran:methanol (1:10, 6.6 mL), a solution benzyl ((2*R*,5*S*,10a*S*,10b*S*)-5-benzyl-10b-hydroxy-8-methoxy-2-methyl-3,6-dioxooctahydro-8*H*-oxazolo[3,2-*a*]pyrrolo[2,1-*c*]pyrazin-2-yl)carbamate (223 mg, 0.45 mmol) in methanol (1.3 mL) was added, and the mixture was evacuated and filled with hydrogen (via balloon). This process was repeated three times. Hydrochloric acid in methanol (1.25 M, 1.44 mL, 1.80 mmol) was added, and the mixture was stirred for 1 h at room temperature. The suspension was filtered through Celite and the filtrate was concentrated azeotropically with dichloromethane and diethyl ether to give (2*R*,5*S*,10a*S*,10b*S*)-2-amino-5-benzyl-10b-hydroxy-8-methoxy-2-methyltetrahydro-8*H*-oxazolo [3,2-*a*] pyrrolo[2,1-*c*]pyrazine-3,6(2*H*,5*H*)-dione hydrochloride (179 mg) as an off-white solid.

To a solution of dihydrolysergic acid hydrochloride (277 mg, 0.90 mmol) in anhydrous dimethylformamide (4.5 mL) under nitrogen, HATU (360 mg, 0.95 mmol) was added, followed by Hünig’s base (349 mg, 0.48 mL, 2.71 mmol), and the mixture was stirred at room temperature for 1.5 h. (2*R*,5*S*,10a*S*,10b*S*)-2-Amino-5-benzyl-10b-hydroxy-8-methoxy-2-methyltetrahydro-8*H*-oxazolo [3,2-*a*] pyrrolo[2,1-*c*]pyrazine-3,6(2*H*,5*H*)-dione hydrochloride (179 mg, 0.45 mmol) was added, and the mixture was stirred for 2.5 h at room temperature. The reaction was quenched with water (15 mL) and extracted with ethyl acetate (3 × 20 mL). The combined organic extracts were washed with water (3 × 15 mL), satd. brine (30 mL), dried (MgSO_4_) and concentrated to give an orange solid (286 mg). This material was purified using automated chromatography under reversed-phase conditions (gradient of 0 → 100% acetonitrile in 0.1% formic acid in water) with detection at 278 nm to give impure (6a*R*,9*R*,10a*R*)-*N*-((2*R*,5*S*,10a*S*,10b*S*)-5-benzyl-10b-hydroxy-8-methoxy-2-methyl-3,6-dioxooctahydro-8*H*-oxazolo[3,2-*a*]pyrrolo[2,1-*c*]pyrazin-2-yl)-7-methyl-4,6,6a,7,8,9,10,10a-octahydroindolo[4,3-*fg*]quinoline-9-carboxamide (160 mg). This material was purified using semi-preparative HPLC (Luna C18(2) (250 mm x 10 mm, 100 Å,10 µm) column eluting with water containing 0.1% HCOOH (A) and acetonitrile (B)) at a flow-rate of 5.0 mL/min and monitored at 280 nm). Separation of the diastereomers was achieved with a gradient ratio starting at 90:10 (A:B), changing to 70:30 (A:B) after 40 min to give (6a*R*,9*R*,10a*R*)-*N*-((2*R*,5*S*,10a*S*,10b*S*)-5-benzyl-10b-hydroxy-8-methoxy-2-methyl-3,6-dioxooctahydro-8*H*-oxazolo [3,2-*a*]pyrrolo[2,1-*c*]pyrazin-2-yl)-7-methyl-4,6,6a,7,8,9,10,10a-octahydroindolo[4,3-*fg*]quinoline-9-carboxamide formate salt (59 mg, 21% over 2 steps from **26**) as a white solid. ^1^H NMR (700 MHz, CD_3_CN): δ 8.20 (s, 1 H, HCO_2_), 7.36 (d, 2 H, *J* = 8.2 Hz, 2 × ArH), 7.24 (t, 2 H, *J* = 7.3 Hz, 2 × ArH), 7.21 (d, 1 H, *J* = 8.1 Hz, ArH), 7.18–7.16 (m, 1 H, ArH), 7.13–7.11 (m, 1 H, ArH), 6.95 (br s, 1 H, ArH), 6.88 (d, 1H, *J* = 7.1 Hz, ArH), 5.52 (dd, 1 H, *J* = 5.0, 1.4 Hz, CHNO), 4.65 (dd, 1 H, *J* = 6.7, 4.7 Hz, α-CH), 3.86 (dd, 1 H, *J* = 8.0, 5.3 Hz, α-CH), 3.43 (dd, 1 H, *J* = 14.5, 4.3 Hz, 0.5 × benzylic CH_2_), 3.30–3.23 (m, 5 H, CH_2_ and OMe), 3.17 (dd, 1 H, *J* = 14.5, 4.3 Hz, 0.5 × benzylic CH_2_), 3.12–3.08 (m, 1 H, 0.5 × CH_2_), 3.04–3.00 (m, 1 H, 0.5 × CH_2_), 2.80–2.73 (m, 2 H, CH_2_), 2.59–2.54 (m, 4 H, NCH_3_ and CH), ca. 2.48 (obscure m, 1 H, CH), 2.17–2.10 (m, 2H, CH_2_), 1.82–1.74 (m, 2 H, CH_2_), 1.61 (q, 1 H, *J* = 12.4 Hz, CH), 1.54 (s, 3 H, CH_3_). ^13^C{^1^H} NMR (175 MHz, CD_3_CN): δ 176.2, 167.2, 167.0, 164.8, 139.9, 134.5, 132.4, 132.3, 131.1, 128.1, 127.5, 126.8, 123.7, 119.6, 113.7, 110.8, 110.1, 104.2, 89.7, 87.1, 67.6, 63.3, 58.7, 57.7, 56.3, 42.5, 42.3, 39.8, 39.5, 31.2, 30.7, 26.5, 24.5, 24.1. LC-MS (ESI positive, *m*/*z*) calcd. for C_34_H_39_N_5_O_6_ [M + H]^+^: 614.30, found: 614.35. rt: 36.1 min.

#### 3.2.18. (6aR,9R,10aR)-N-((2R,5S,8R,10aS,10bS)-5-Benzyl-8,10b-dihydroxy-2-methyl-3,6-dioxooctahydro-8H-oxazolo[3,2-a]pyrrolo[2,1-c]pyrazin-2-yl)-7-methyl-4,6,6a,7,8,9,10,10a-octahydroindolo[4,3-fg]quinoline-9-carboxamide Hydrochloride/Formate (4:6) Salt and (8S)-epimer (**3a**)/(**3b**)

To a solution of (6a*R*,9*R*,10a*R*)-*N*-((2*R*,5*S*,8*R*,10a*S*,10b*S*)-5-benzyl-10b-hydroxy-8-methoxy-2-methyl-3,6-dioxooctahydro-8*H*-oxazolo[3,2-*a*]pyrrolo[2,1-*c*]pyrazin-2-yl)-7-methyl-4,6,6a,7,8,9,10,10a-[4,3-*fg*]quinoline-9-carboxamide (59 mg, 97 µmol) in acetonitrile (1.6 mL), 1 M hydrochloric acid (6.7 mL) was added, and the mixture was stirred for 5 h at room temperature. This material was purified using automated chromatography under reversed-phase conditions (gradient of 0 → 100% acetonitrile in 0.1% formic acid water) with detection at 278 nm to give (6a*R*,9*R*,10a*R*)-*N*-((2*R*,5*S*,8*R*,10a*S*,10b*S*)-5-benzyl-8,10b-dihydroxy-2-methyl-3,6-dioxooctahydro-8*H*-oxazolo[3,2-*a*]pyrrolo[2,1-*c*]pyrazin-2-yl)-7-methyl-4,6,6a,7,8,9,10,10a-octahydroindolo[4,3-*fg*]quinoline-9-carboxamide hydrochloride/formate (4:6) salt and (8*S*)-epimer (52 mg, 90%) as a white solid after lyophilization. ^1^H NMR (400 MHz, DMSO-d_6_): δ 10.7 (s, 1 H, NH indole), 9.37 (s, 0.6 H, 0.6 × NH), 9.32 (s, 0.4 H, 0.4 × NH), 8.14 (s, 0.6 H, 0.6 × HCO_2_), 7.34–7.30 (m, 2 H, 2 × ArH), 7.21 (q, 2 H, *J* = 7.6 Hz, 2 × ArH), 7.16–7.12 (m, 1 H, ArH), 7.07–7.03 (m, 1 H, ArH), 7.00 (br s, 1 H, ArH), 6.81 (d, 1H, *J* = 7.1 Hz, ArH), 6.72 (br s, 0.4 × OH), 6.66 (br s, 0.6 H, 0.6 × OH), 6.03 (d, 0.4 H, *J* = 5.1 Hz, 0.4 × OH), 6.02 (d, 0.6 H, *J* = 4.9 Hz, 0.6 × OH), 5.62 (dt, 0.6 H, *J* = 4.7, 5.0 Hz, 0.6 × CHNO), 5.43 (t, 0.4 H, *J* = 5.2 Hz, 0.6 × CHNO), 4.52 (dd, 0.6 H, *J* = 6.9, 4.4 Hz, 0.6 × α-CH), 4.47 (dd, 0.4 H, *J* = 6.5, 5.4 Hz, 0.4 × α-CH), 3.94 (td, 0.6 H, *J* = 7.2, 1.9 Hz, 0.6 × α-CH), 3.67 (ddd, 0.4 H, *J* = 11.4, 5.2, 1.8 Hz, 0.4 × α-CH), 3.22–3.17 (m, 1 H, 0.5 × benzylic CH_2_), 3.11–3.03 (m, 1 H, 0.5 × benzylic CH_2_), 2.91–2.84 (m, 1.8 H, 1.8 × NCH_3_), 2.72–2.65 (m, 1.2 H, 1.2 × NCH_3_), 2.60–2.55 (m, 1 H, 0.5 × CH_2_), ca. 2.44 (obscure m, 1 H, CH), 2.12–2.07 (m, 1 H, 0.5 × CH_2_), 2.05–1.97 (m, 2 H, CH_2_), 1.93–1.88 (m, 1 H, 0.5 × CH_2_), 1.85–1.81 (m, 1 H, 0.5 × CH_2_), 1.79–1.75 (m, 0.6 H, 0.6 × CH), 1.74–1.71 (m, 0.4 H, 0.4 × CH), 1.61–1.56 (m, 1 H, CH), 1.53–1.44 (m, 4 H, 3 H, CH_3_ and CH). ^13^C{^1^H} NMR (100 MHz, DMSO-d_6_): δ 166.0, 156.9, 165.0, 164.8, 163.1, 138.9, 138.6, 133.2, 129.8, 129.7, 127.8, 127.7, 126.0, 125.8, 122.1, 118.7, 112.0, 108.9, 102.8, 102.6, 85.6, 85.7, 80.9, 79.6, 66.3, 64.4, 62.3, 56.4, 56.1, 38.3, 37.8, 31.2, 30.5, 23.9, 23.0. Minor isomer HRMS (ESI positive, *m*/*z*) calcd. for C_33_H_37_N_5_O_6_ [M + H]^+^: 600.2817, found: 600.2811. Major isomer HRMS (ESI positive, *m*/*z*) calcd. for C_33_H_37_N_5_O_6_ [M + H]^+^: 600.2817, found: 600.2811.

#### 3.2.19. (6aR,9R,10aR)-N-((2R,5S,8R,10aS,10bS)-5-((phenyl-d5)methyl)-8,10b-dihydroxy-2-methyl-3,6-dioxooctahydro-8H-oxazolo[3,2-a]pyrrolo[2,1-c]pyrazin-2-yl)-7-methyl-4,6,6a,7,8,9,10,10a-octahydroindolo[4,3-fg]quinoline-9-carboxamide Hydrochloride/Formate (4:6) Salt and (8S)-epimer (**^2^H_5_-3a**)/(**^2^H_5_-3b**)

To a solution of (6a*R*,9*R*,10a*R*)-*N*-((2*R*,5*S*,8*R*,10a*S*,10b*S*)-5-((phenyl-d5)methyl)-10b-hydroxy-8-methoxy-2-methyl-3,6-dioxooctahydro-8*H*-oxazolo[3,2-*a*]pyrrolo[2,1-*c*]pyrazin-2-yl)-7-methyl-4,6,6a,7,8,9,10,10a-octahydroindolo[4,3-*fg*]quinoline-9-carboxamide (22 mg, 36 µmol) in acetonitrile (0.62 mL), 1 M hydrochloric acid (2.6 mL) was added, and the mixture was stirred for 5 h at room temperature. The product in the resulting solution was purified using automated chromatography under normal phase conditions (gradient of 0 → 100% acetonitrile in 0.1% formic acid water) with detection at 278 nm to give (6a*R*,9*R*,10a*R*)-*N*-((2*R*,5*S*,10a*S*,10b*S*)-5-((phenyl-d5)methyl)-8,10b-dihydroxy-2-methyl-3,6-dioxooctahydro-8*H*-oxazolo[3,2-*a*]pyrrolo[2,1-*c*]pyrazin-2-yl)-7-methyl-4,6,6a,7,8,9,10,10a-octahydroindolo[4,3-*fg*]quinoline-9-carboxamide hydrochloride/formate (4:6) salt (20 mg, 95%) as a white solid after lyophilization. ^1^H NMR (700 MHz, DMSO-d_6_): δ 10.7 (s, 1 H, NH indole), 9.37 (s, 0.6 H, 0.6 × NH), 9.32 (s, 0.4 H, 0.4 × NH), 8.17 (s, 0.6 H, 0.6 × HCO_2_), 7.17–7.13 (m, 1 H, ArH), 7.08–7.03 (m, 1 H, ArH), 7.01 (br s, 1 H, ArH), 6.82 (d, 1H, *J* = 7.3 Hz, ArH), 6.71 (br s, 0.4 × H, OH), 6.65 (br s, 0.6 H, 0.6 × H, OH), 6.03 (d, 0.4 H, *J* = 5.1 Hz, 0.4 × OH), 6.02 (d, 0.6 H, *J* = 4.8 Hz, 0.6 × OH), 5.62 (dt, 0.6 H, *J* = 4.6, 5.1 Hz, 0.6 × CHNO), 5.44 (t, 0.4 H, *J* = 5.3 Hz, 0.6 × CHNO), 4.52 (dd, 0.6 H, *J* = 6.9, 4.5 Hz, 0.6 × α-CH), 4.47 (dd, 0.4 H, *J* = 6.5, 5.4 Hz, 0.4 × α-CH), 3.94 (td, 0.6 H, *J* = 7.2, 1.9 Hz, 0.6 × α-CH), 3.68 (ddd, 0.4 H, *J* = 11.4, 5.2, 1.8 Hz, 0.4 × α-CH), 3.19 (ddd, 1 H, *J* = 13.9, 6.8, 5.0 Hz, 0.5 × benzylic CH_2_), 3.07 (ddd, 1 H, *J* = 23.1, 13.9, 4.9 Hz, 0.5 × benzylic CH_2_), 2.93–2.82 (m, 1.8 H, 1.8 × NCH_3_), 2.73–2.66 (m, 1 H, 1.2 × NCH_3_), ca. 2.52 (obscure m, 1 H, 0.5 × CH_2_), ca. 2.44 (obscure m, 1H, CH), 2.12–2.06 (m, 1 H, 0.5 × CH_2_), 2.05–1.97 (m, 2 H, CH_2_), 1.93–1.88 (m, 1 H, 0.5 × CH_2_), 1.85–1.81 (m, 1 H, CH), 1.80–1.75 (m, 0.6 H, 0.6 × CH), 1.74–1.70 (m, 0.4 H, 0.4 × CH), 1.62–1.56 (m, 1 H, CH), 1.53–1.44 (m, 3 H, CH_3_). Minor isomer HRMS (ESI positive, *m*/*z*) calcd. for C_33_H_32_D_5_N_5_O_6_ [M + H]^+^: 605.3125, found: 605.3125. Major isomer HRMS (ESI positive, *m*/*z*) calcd. for C_33_H_32_D_5_N_5_O_6_ [M + H]^+^: 605.3125, found: 605.3125. The ^1^H NMR spectrum showed no phenyl resonances consistent with the compound’s origin from [2,3,4,5,6-^2^H_5_]L-phenylalanine (Merck Darmstadt, Germany: ≥98 atom % D) and there was no detectable d4 component in the HRMS.

#### 3.2.20. Dihydrolysergic Acid Hydrochloride (**30**)

To a suspension of 10% Pd on carbon (0.70 g, 40% weight) in methanol (15 mL), a solution of D-lysergic acid methyl ester (500 mg, 1.77 mmol) in methanol (5 mL) was added, and the mixture was evacuated and filled with hydrogen (via balloon). This process was repeated three times, and the mixture was heated at 35 °C overnight. The suspension was filtered through Celite, and the filtrate was concentrated to give a brown residue. This material was purified using automated chromatography under normal phase conditions (gradient of 0 → 100% ethyl acetate in petrol) with detection at 278 nm to give dihydrolysergic acid methyl ester (337 mg, 67%) as a pale-yellow solid. TLC: R*_f_* = 0.29 (methanol—ethyl acetate, 5:95 *v*/*v*). ^1^H NMR data [44]. 

To a solution of dihydrolysergic acid methyl ester (0.68 g, 2.38 mmol) in methanol (12 mL), 1 M aqueous sodium hydroxide solution (11.9 mL, 11.9 mmol) was added, and the mixture was heated at 40 °C for 3 h. The reaction mixture was cooled to room temperature and adjusted to pH 6 with 1 M hydrochloric acid. The solution was concentrated to give a brown solid. This material was suspended in ice-cold water, and the resulting solid was collected by Büchner filtration to give a beige solid. The solid was suspended in acetonitrile (20 mL) and stirred for 1 h at room temperature. The solid was collected by Büchner filtration to give dihydrolysergic acid (478 mg, 74%) as an off-white solid. TLC: R*_f_* = 0.29 (methanol—ethyl acetate, 5: 95 *v*/*v*). ^1^H NMR data [44].

To a suspension of dihydrolysergic acid (316 mg, 1.17 mmol) in acetonitrile: water (2.5:1, 8 mL), 1 M hydrochloric acid was added, and the reaction mixture was stirred for 15 min at room temperature. The resulting solid collected by Büchner filtration was washed with acetonitrile (3 × 5 mL) to give dihydrolysergic acid hydrochloride (291 mg, 81%) as an off-white solid.

## 4. Conclusions

The 20-step synthesis described has been repeated several times, starting from protected glutamic acid on a 10 g scale, and has also been used to prepare **^2^H_5_-3a** starting from d_5_-phenylalanine (see Materials and Methods). Multiple challenges were overcome during the 8-hydroxy-dihydroergotamine syntheses described and serve as a testament to the excellence of the pioneering work of Hofmann and many others cited above, who laboured without modern methods of purification and structural characterisation.

## Data Availability

The original contributions presented in this study are included in the article and Supplementary Materials. Further inquiries can be directed to the corresponding author.

## Supplementary Materials

The following supporting information can be downloaded at https://www.mdpi.com/article/10.3390/molecules31091547/s1: HRMS of Compounds **3a**/**3b **and (^2^H_5_-3a)/(^2^H_5_-3b) S2–S32, NMR spectra of all compounds S33–S50, Crystallography data S51–S52.CCDC 2469731 contains the supplementary crystallographic data for this paper, which can be obtained from The Cambridge Crystallographic Data Centre via www.ccdc.cam.ac.uk/structures (accessed on 20 March 2026). Figure S1. A ball and stick depiction of the packing observed in **19**, as viewed along the [011] direction. The C(6) hydrogen bonding interactions are depicted by cyan dotted lines. Key: N–blue, O–red, C–grey, H–white. Table S1. Crystallographic tables for **19**.

## References

[1] Schiff P.L. (2006). Ergot and its alkaloids. Am. J. Pharm. Educ..

[2] Ashfaq M., Mushtaq I., Mehmood M.A., Ahmad F., ur Rehman S., Abd-Elsalam K.A., Mohamed H.I. (2024). Ergot alkaloids from Claviceps: Production and pharmacological properties. Nanobiotechnology for Plant Protection, Fungal Secondary Metabolites.

[3] Jastrzębski M.K., Kaczor A.A., Wróbel T.M. (2022). Methods of lysergic acid synthesis—The key ergot alkaloid. Molecules.

[4] Stoll A. (1921). Highly Active Preparation of Ergot and Process of Making Same. Patent.

[5] Stoll A. (1950). Recent investigations on ergot alkaloids. Chem. Rev..

[6] Wisniak J. (2024). Charles-Joseph Tanret: Natural Principles. Educ. Química.

[7] Barger G., Carr F.H. (1907). The alkaloids of ergot. J. Chem. Soc..

[8] Barger G. (1937). The alkaloids of ergot. Analyst.

[9] Pehlivanlar E., Carradori S., Simsek R. (2024). Migraine and its treatment from the medicinal chemistry perspective. ACS Pharmacol. Transl. Sci..

[10] Shrewsbury S.B. (2026). DHE—past, present, and future: A narrative review. Pain Manag..

[11] Tfelt-Hansen P.C., Koehler P.J. (2008). History of the use of ergotamine and dihydroergotamine in migraine from 1906 and onward. Cephalalgia.

[12] Hanoun N., Saurini F., Lanfumey L., Hamon M., Bourgoin S. (2003). Dihydroergotamine and its metabolite, 8’-hydroxy-dihydroergotamine, as 5-HT_1A_ receptor agonists in the rat brain. Br. J. Pharmacol..

[13] Wang C., Jiang Y., Ma J., Wu H., Wacker D., Katritch V., Han G.W., Liu W., Huang X.P., Vardy E. (2013). Structural basis for molecular recognition by serotonin receptors. Science.

[14] Stoll A., Hoffman A. (1943). Die dihydroderivate der naturlichen linksdrehenden mutterkornalkaloide. Helv. Chim. Acta..

[15] Giger R.K.A., Engel G. (2006). Albert Hofmann’s pioneering work on ergot alkaloids and its impact on the search for novel drugs at Sandoz, a predecessor company of Novartis. Chimia.

[16] Ailani J., Charleston L., Strom S., Albrecht D., Cowan R. (2025). Study participant impression of use and satisfaction with STS101 (dihydroergotamine nasal powder): Results from the open-label ASCEND acute migraine study. Headache.

[17] Maurer G., Frick W. (1984). Elucidation of the structure and receptor binding studies of the major primary metabolite of dihydroergotamine in man. Eur. J. Clin. Pharmacol..

[18] Pakhomova S., Ondrâcek J., Kratochvil B., Jegorov A., Cvak L. (1996). The structure of ergot alkaloid hydroxyergotamine. Z. Für Krist.-Cryst. Mater..

[19] Ott H., Frey A.J., Hofmann A. (1963). The stereospecific cyclolization of *N*-(α-hydroxyacyl)phenylalaninyl-proline lactams. Tetrahedron.

[20] Shemyakin M.M., Antonov V.K., Shkrob A.M., Sheinker Y.N., Senyavina L.B. (1962). Cyclol formation in peptide systems tautomerism of *N*-(α-hydroxyacyl)-amides. Tetrahedron Lett..

[21] Glenn A.L. (1954). The structure of the ergot alkaloids. Quart. Rev..

[22] Jacobs W.A., Craig L.C. (1934). The degradation of ergotinine with alkali. Lysergic acid. J. Biol. Chem..

[23] Hebert H. (1979). The crystal structure and absolute configuration of (-)-dihydroergotamine methanesulfonate monohydrate. Acta Crystallogr..

[24] Pakhomova S., Ondrâcek J., Husak M., Kratochvil B., Jegorov A., Cvak L., Sedmera P., Havhcek V. (1997). Conformation of ergopeptam and ergopeptine alkaloids (ergocristam and ergocristine). Z. Für Krist.-Cryst. Mater..

[25] Monch B., Kraus W., Köppen R., Emmerling F. (2016). The different conformations and crystal structures of dihydroergocristine. J. Mol. Struct..

[26] Hofmann A., Frey A.J., Ott H. (1961). Die totalsynthese des Ergotamins. Experientia.

[27] Giger R.K.A., Loosli H.R., Walkinshaw M.D., Clark B.J., Vigouret J.M. (1987). A new semisynthetic ergot peptide alkaloid: DCN 203-922. Experientia.

[28] Bauermeister A., Aguiar F.A., Marques L.M.M., Dos Santos Malta J., Barros F., Callejon D.R., Moraes de Oliveira A.R., Lopes N.P. (2016). In vitro metabolism evaluation of the ergot alkaloid dihydroergotamine: Application of microsomal and biomimetic oxidative model. Planta Med..

[29] Kellerman D.J., Armer T., Zhang J. (2014). 8’-Hydroxy-Dihydroergotamine Compounds and Composition. Patent.

[30] Chen X., Zhong D., Xu H., Schug B., Blume H. (2002). Sensitive and specific chromatographic-tandem mass spectrometric assay for dihydroergotamine and its major metabolite in human plasma. J. Chromatogr. B Biomed. Sci. Appl..

[31] Wang L., Zha Z., Qu W., Qiao H., Lieberman B.P., Plössl K., Kung H.F. (2012). Synthesis and evaluation of ^18^F labeled alanine derivatives as potential tumor imaging agents. Nucl. Med. Biol..

[32] Dubey A., Kandula S.R.V., Kumar P. (2008). Dimethyl sulfoxide pivaloyl chloride: A new reagent for oxidation of alcohols to carbonyls. Synth. Commun..

[33] Ozanne A., Pouysegu L., Depernet D., Francüois B., Quideau S. (2003). A stabilized formulation of IBX (SIBX) for safe oxidation reactions including a new oxidative demethylation of phenolic methyl aryl ethers. Org. Lett..

[34] Van Arman S.A. (2009). 2-Methyl-2-propanol as solvent for o-iodoxybenzoic acid (IBX) oxidation of 1° alcohols to aldehydes. Tetrahedron Lett..

[35] Jacobi P.A., Lee K. (1997). Total synthesis of (±)-stemoamide. J. Am. Chem. Soc..

[36] Guanti G., Banfi L., Powles K., Rasparini M., Scolastico C., Fossati N. (2001). Asymmetric synthesis of (*R*)-(−)-chlozolinate through a chemoenzymatic procedure. Tetrahedron Asym..

[37] Stadler P.A., Guttmann S., Hauth H., Huguenin R.L., Sandrin E., Wersin G., Willems H., Hofmann A. (1969). Die Synthese der Alkaloide der Ergotoxin-Gruppe. 70. Mitteilung über Mutterkornalkaloide. Helv. Chim. Acta..

[38] Ghosh A.K., Brindisi M., Sarkar A. (2018). The Curtius rearrangement: Applications in modern drug discovery and medicinal chemistry. Chem. Med. Chem..

[39] Jang D.O., Park D.J., Kim J. (1999). A Mild and efficient procedure for the preparation of acid chlorides from carboxylic acids. Tetrahedron Lett..

[40] Kaiser C., Weinstock J. (1971). Amines from mixed carboxylic-carbonic anhydrides: Phenylcyclopentylamine. Org. Synth..

[41] Dubé P., Nathel N.F.F., Vetelino M., Couturier M., Larrivée Aboussafy C., Pichette S., Jorgensen M.L., Hardink M. (2009). Carbonyldiimidazole-mediated Lossen rearrangement. Org. Lett..

[42] Kinoyama I., Taniguchi N., Toyoshima A., Nozawa E., Kamikubo T., Imamura M., Matsuhisa A., Samizu K., Kawanimani E., Niimi T. (2006). (+)-(*2R*,*5S*)-4-[4-Cyano-3-(trifluoromethyl)phenyl]-2,5-dimethyl-*N*-[6-(trifluoromethyl)pyridin-3- yl]piperazine-1-carboxamide (YM580) as an orally potent and peripherally selective nonsteroidal androgen receptor antagonist. J. Med. Chem..

[43] Green M., Lucken E.A.C. (1961). Die Struktur des Pyroergotamins. Helv. Chim. Acta..

[44] Wagger J., Požes A., Požgan F. (2013). Synthesis of European Pharmacopoeial Impurities A, B, C, and D of cabergoline. RSC Adv..

[45] Lee K., Poudel Y.B., Glinkerman C.M., Boger D.L. (2015). Total Synthesis of dihydrolysergic acid and dihydrolysergol: Development of a divergent synthetic strategy applicable to rapid assembly of D-ring analogs. Tetrahedron.

[46] Due-Hansen M.E., Pandey S.K., Christiansen E., Andersen R., Hansen S.V.F., Ulven T. (2016). A protocol for amide bond formation with electron deficient amines and sterically hindered substrates. Org. Biomol. Chem..

[47] Giger R.K.A. (1988). Ergot Alkaloids for Treating Senile Dementia. Patent.

[48] Magó née Karácsony E., Borsi J., Balogh T., Wolf L. (1975). New Dihydro-Lysergic Acid Derivative. Patent.

[49] Frey A. (1963). Lysergic Acid Halide Hydrohalides. Patent.

[50] Moldvai I., Temesvári-Major E., Incze M., Szentirmay É., Gács-Baitz E., Szántay C. (2004). Enantioefficient synthesis of α-ergocryptine:  first direct synthesis of (+)-lysergic acid. J. Org. Chem..

[51] Maurer G., Schreier E., Delaborde S., Loosli H.R., Nufer R., Shukla A.P. (1982). Fate and disposition of bromocriptine in animals and man I: Structural elucidation of metabolites. Eur. J. Drug Metab. Pharmacokin..

