# Supplementary material for: Total Synthesis of 8-Hydroxy-dihydroergotamine, the Major Human Metabolite of Dihydroergotamine
Source: Molecules. 2026 May 6;31(9):1547. doi: 10.3390/molecules31091547 (PMC13164584; doi:10.3390/molecules31091547)
Supplement: Supplementary file 1 [file molecules-31-01547-s001.zip › molecules-4240982-supplementary.pdf]

Supporting Information

Total Synthesis of 8-Hydroxy-dihydroergotamine, the Major Human Metabolite of Dihydroergotamine

Manuel Monerris Mascaro[1], Alistair P. Henderson[1], Marta Drozdowska[1], Rachel Richardson[1], Dylan Nagel-Savage[1], Michael J. Hall[2,3], Alexandra Longcake[2,3], Lina Mardiana[2,3,4], and Bernard T. Golding*[1,2]

[1] Sterling Newcastle, The Biosphere, Drayman's Way, Newcastle Helix, Corporation Street, Newcastle upon Tyne, NE4 5BX, UK

[2] School of Natural and Environmental Sciences - Chemistry, Bedson Building, Newcastle University, Newcastle upon Tyne, NE1 7RU, UK; E-mail: bernard.golding@ncl.ac.uk

[3] Indicatrix Crystallography Ltd, Newcastle University, Newcastle upon Tyne, NE1 7RU, UK.

[4] Department of Chemistry, Universitas Indonesia, Depok, Jawa Barat, 16424, Indonesia.

Table of contents Page

1. HRMS of Compounds **3a**/**3b** and (**^{2}H$_5$-3a**)/(**^{2}H$_5$-3b**)…………...…………………..S2
2. NMR Spectra of all compounds………………………………………………….S33
3. Crystallography data…………………………………………………….…S51

1. HRMS of Compounds 3a/3b and ($^2H_5$-3a)/($^2H_5$-3b)

SAgE Mass Spectrometry | HRMS Analysis

Newcastle University | Research Facilities

Sample Reference: 8-OH-DHE (2)
Submitted By: Alex Charlton
Data File: 8OH-DHE.swrx
Experiment: HR-MS 1: Small Molecules (Polar) - Electrospray Ionisation (ESI; 100-1500 Da)

Target Confirmation Summary

	Name	Formula	Adduct	Calculated *m/z*	Measured *m/z*	Response	Found RT	Error (mmu)	Error (ppm)	Target Status	Within Error Limits
1	8-OH-DHE	C33H37N5O6	[M+H]+	600.2817	600.2811	2,089,371,953.29	3.6262	-0.56	-0.93	Found	Yes
2	8-OH-DHE	C33H37N5O6	[M+H]+	600.2817	600.2811	3,246,488,827.10	3.8287	-0.56	-0.93	Found	Yes
3	8-OH-DHE	C33H37N5O6	[M+Na]+	622.2636	622.2633	20,564,108.17	3.6262	-0.30	-0.49	Found	Yes
4	8-OH-DHE	C33H37N5O6	[M+Na]+	622.2636	622.2633	27,636,374.74	3.8287	-0.30	-0.49	Found	Yes
5	8-OH-DHE	C33H37N5O6	[M+NH4]+	617.3082	0.0000	0.00	0.0000			Not Found	
6	8-OH-DHE	C33H37N5O6	[M]+	599.2738	599.2728	1,905,707.88	3.8287	-1.04	-1.73	Found	Yes
7	8-OH-DHE	C33H37N5O6	[M-H]-	598.2671	598.2670	414,122,366.55	3.8035	-0.11	-0.18	Found	Yes

An error of < 5 ppm indicates that the measured mass is consistent with the proposed formula.

ge Result - 8-OH-DHE

8OH-DHE - 8-OH-DHE, *m/z* 600.2817 ± 0.00250, FTMS + c ESI Full ms [100.0000-1500.0000]

NL: 8.445E08

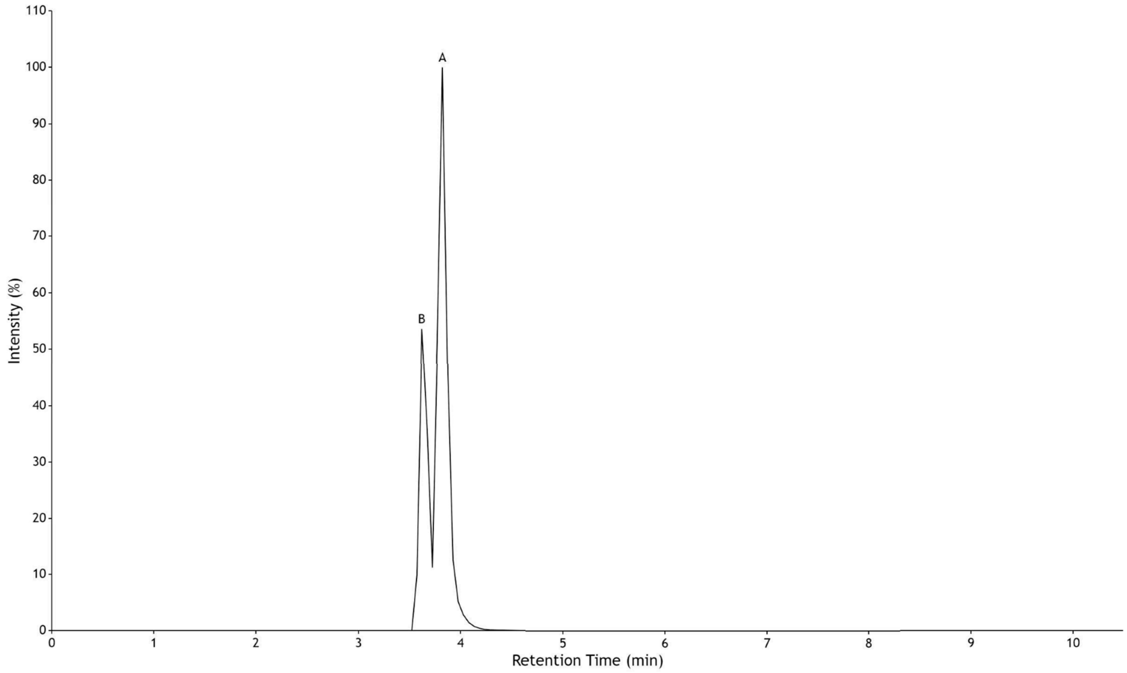

8OH-DHE, Base Peak, FTMS + c ESI Full ms [100.0000-1500.0000]

NL: 8.445E08

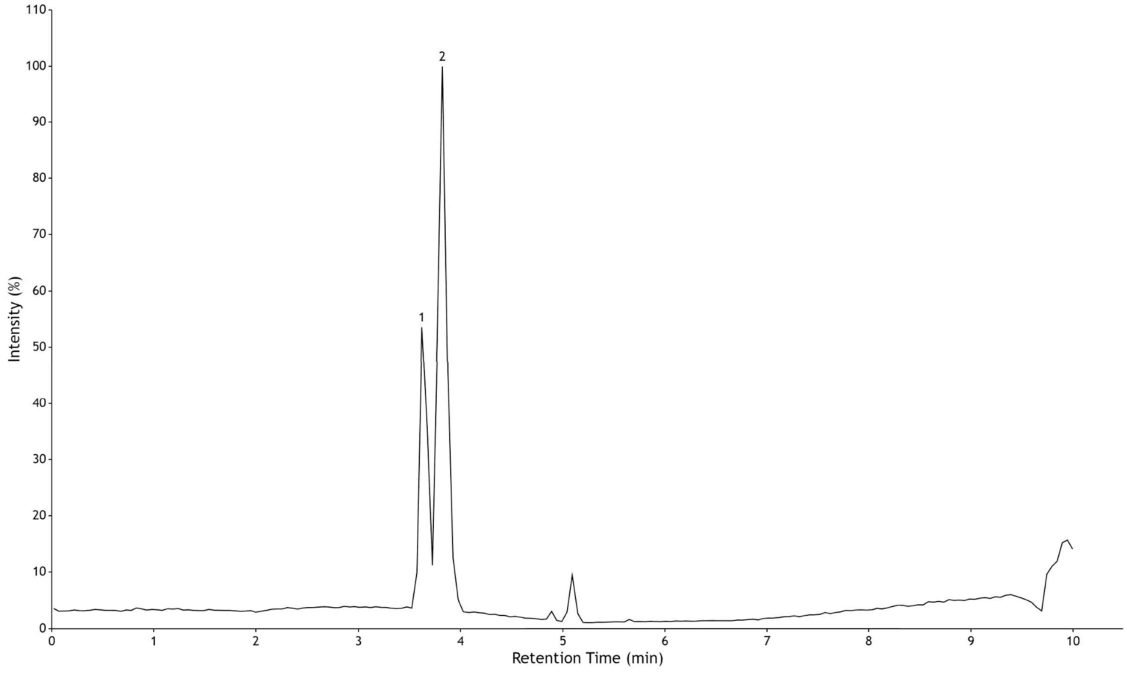

Page 2 of 16

ge Peak A Average Spectrum

8-OH-DHE

RT: 3.8287, NL: 2.378E08, BP: 600.2811, Match Score: 67.23

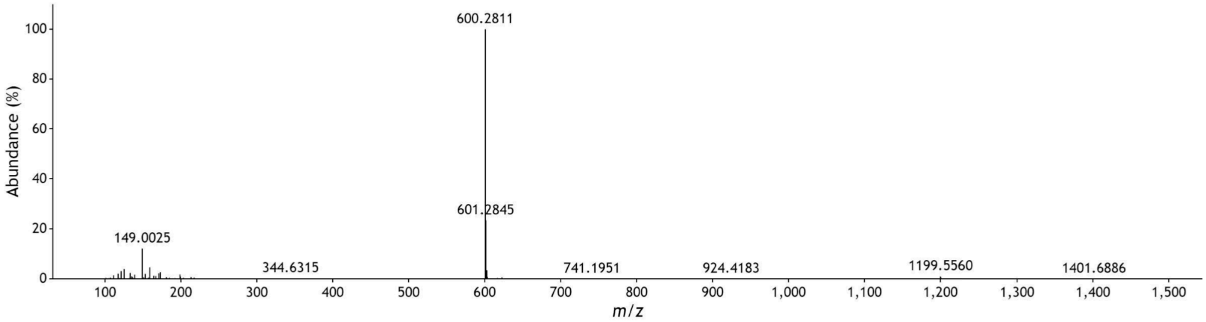

Expanded Target Peak A Average Spectrum

8-OH-DHE

RT: 3.8287, NL: 2.378E08, BP: 600.2811, Match Score: 67.23

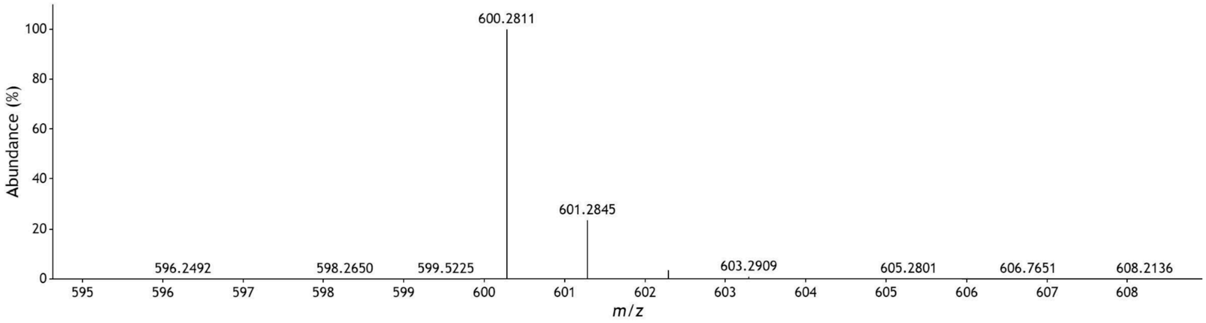

Theoretical Spectrum

C33H37N5O6 [M+H]+

BP: 600.2817

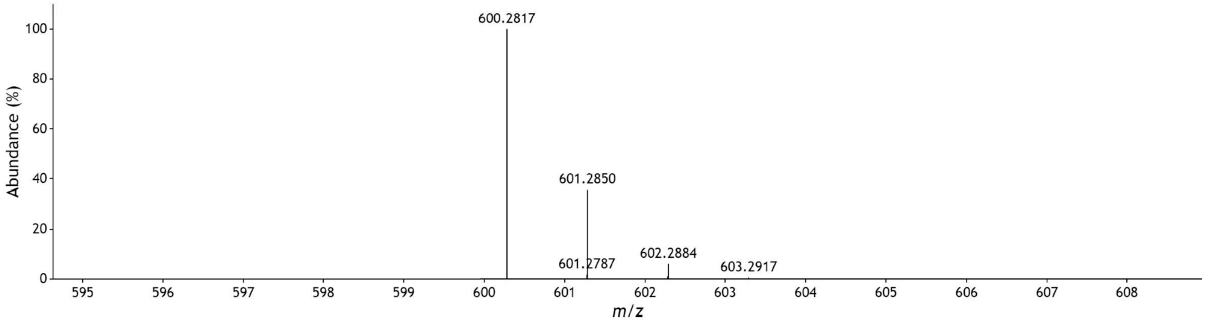

ge Peak B Average Spectrum

8-OH-DHE

RT: 3.6262, NL: 2.281E08, BP: 600.2811, Match Score: 0.93

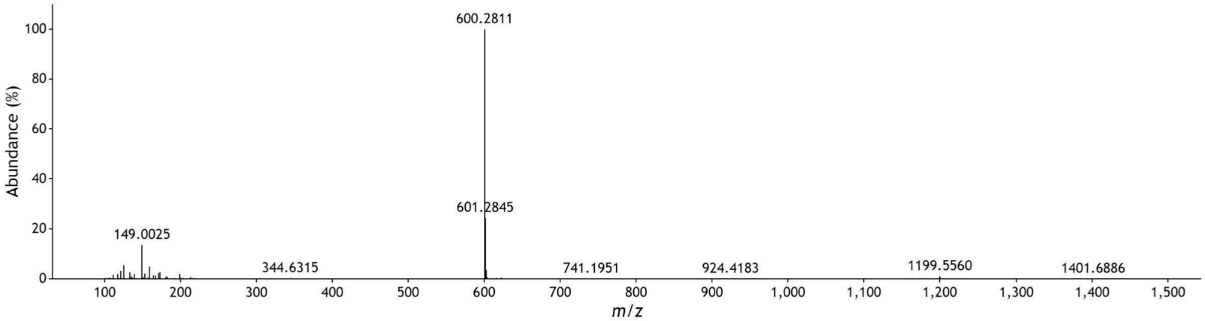

Expanded Target Peak B Average Spectrum

8-OH-DHE

RT: 3.6262, NL: 2.281E08, BP: 600.2811, Match Score: 0.93

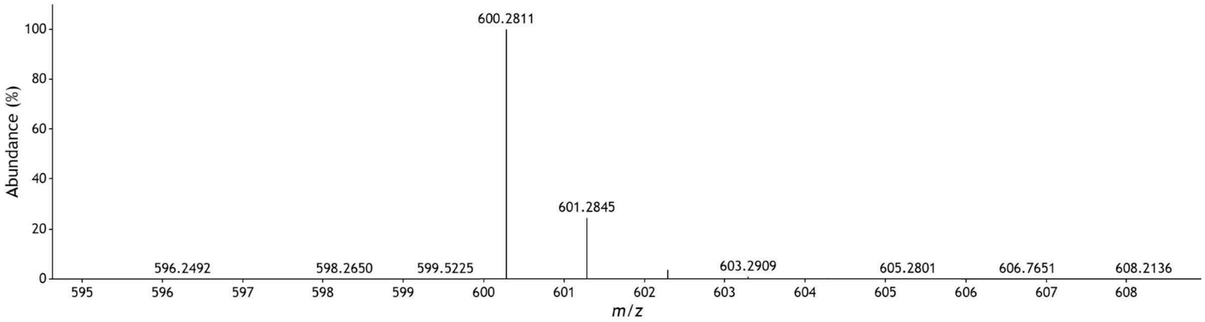

Theoretical Spectrum

C33H37N5O6 [M+H]+

BP: 600.2817

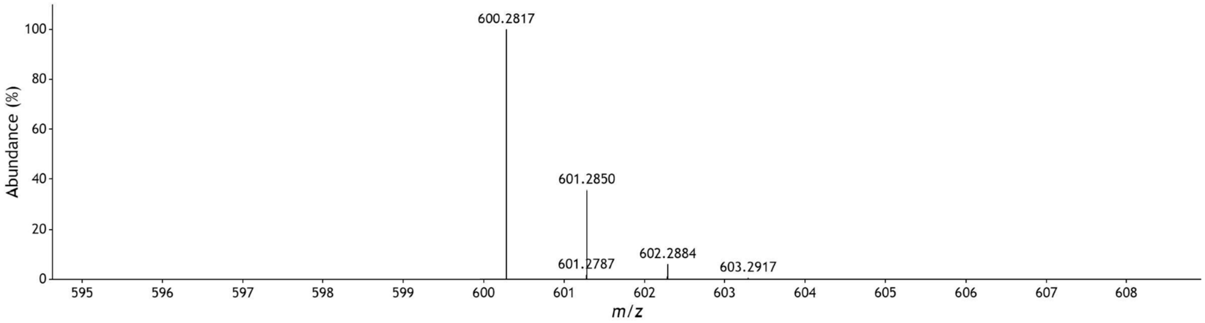

ge Structure

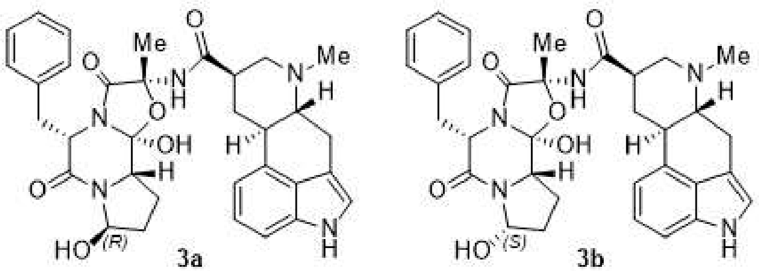

Chemical Formula: C33H37N5O6Exact Mass: 599.27

Target 8-OH-DHE | C33H37N5O6 [M+H]+ | Calculated *m/z* 600.2817 | Confirmation Trace - Base Peak

	Found RT	Area	Height	Area %	Total %	Target Peak	Measured *m/z*	Error (mmu)	Error (ppm)
1	3,6262	1,851,343,893,29	421,278,300,00	40,15	28,65	B	600,2811	-0,56	-0,93
2	3,8287	4,610,857,101,59	784,122,436,00	100,00	71,35	A	600,2811	-0,56	-0,93

ge Result - 8-OH-DHE

8OH-DHE - 8-OH-DHE, *m/z* 622.2636 ± 0.00250, FTMS + c ESI Full ms [100.0000-1500.0000]

NL: 5.199E06

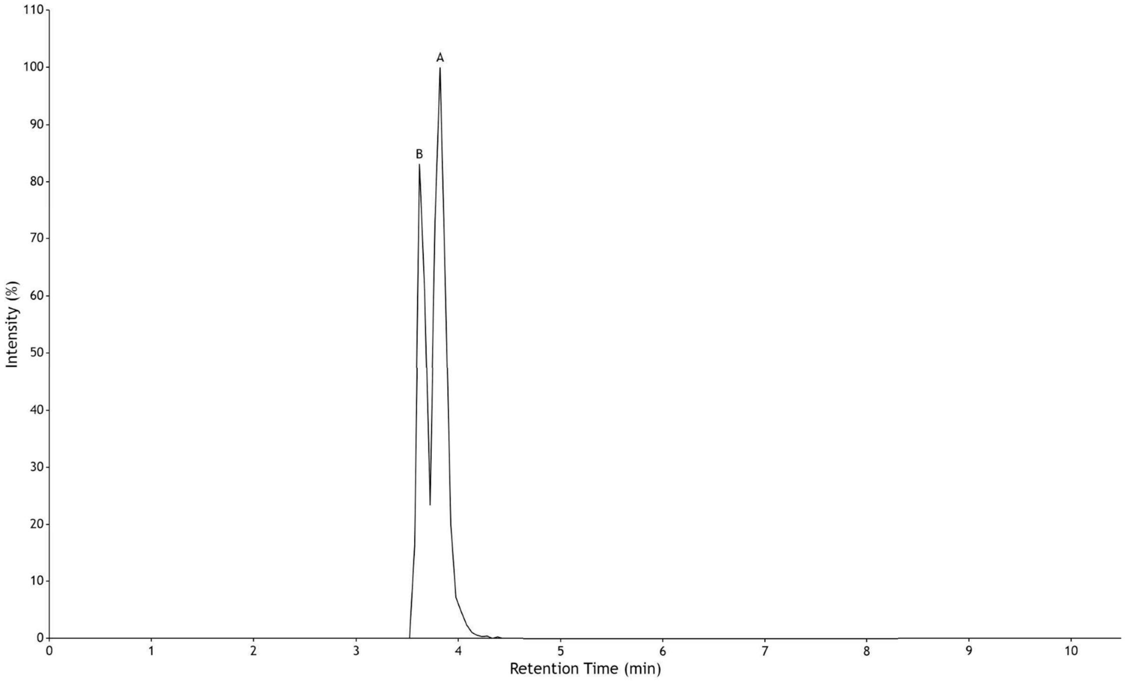

8OH-DHE, Base Peak, FTMS + c ESI Full ms [100.0000-1500.0000]

NL: 8.445E08

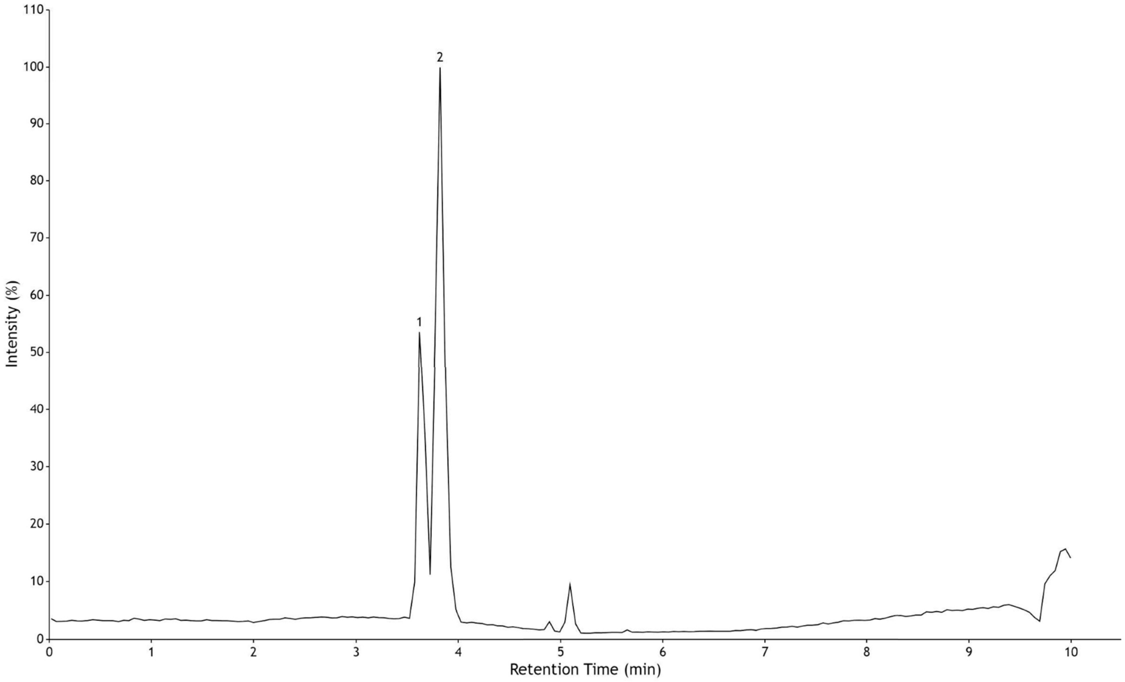

ge Peak A Average Spectrum

8-OH-DHE

RT: 3.8287, NL: 2.378E08, BP: 600.2811, Match Score: 41.03

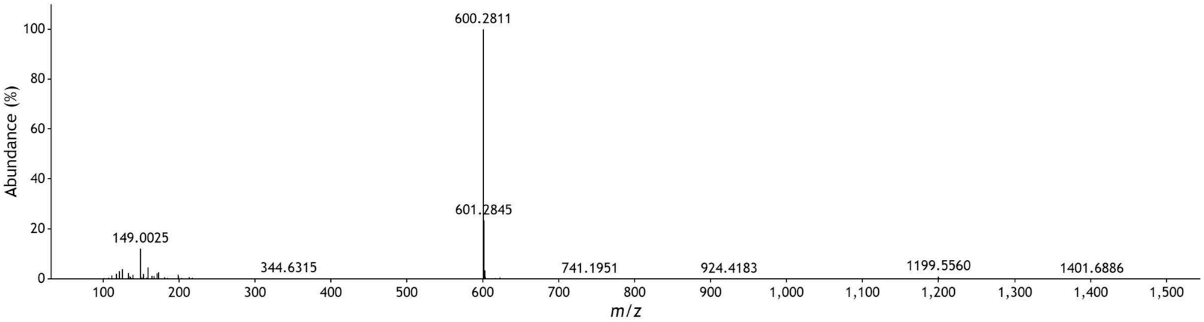

Expanded Target Peak A Average Spectrum

8-OH-DHE

RT: 3.8287, NL: 2.378E08, BP: 600.2811, Match Score: 41.03

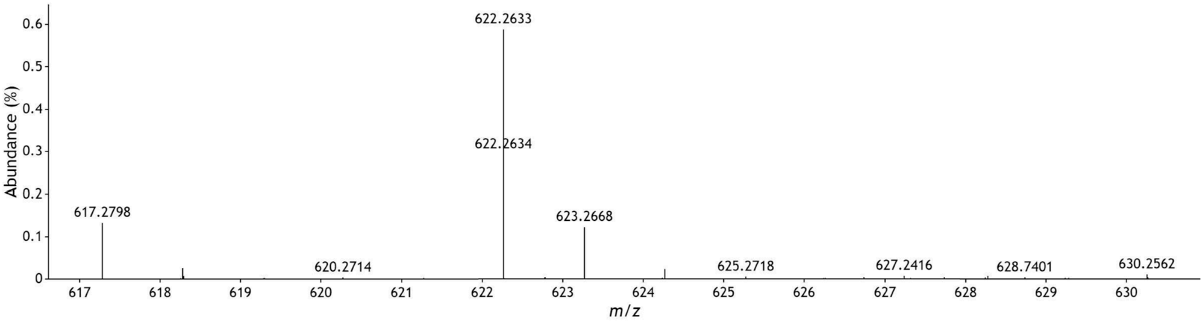

Theoretical Spectrum

C33H37N5O6 [M+Na]+

BP: 622.2636

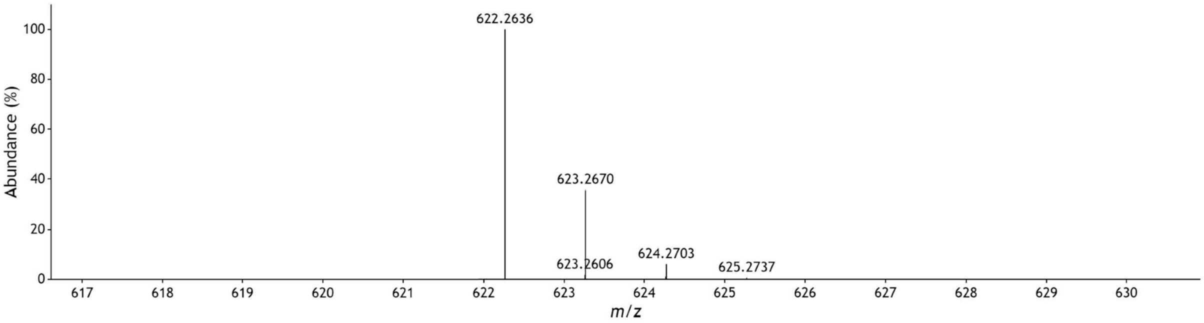

ge Peak B Average Spectrum

8-OH-DHE

RT: 3.6262, NL: 2.281E08, BP: 600.2811, Match Score: 99.95

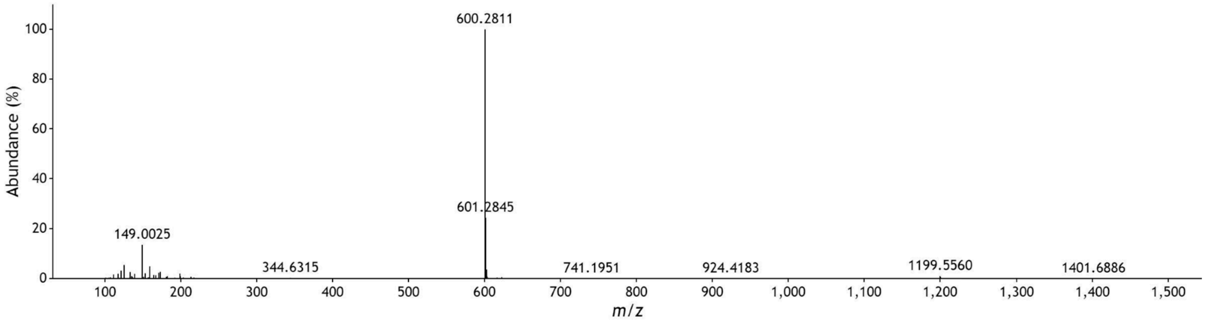

Expanded Target Peak B Average Spectrum

8-OH-DHE

RT: 3.6262, NL: 2.281E08, BP: 600.2811, Match Score: 99.95

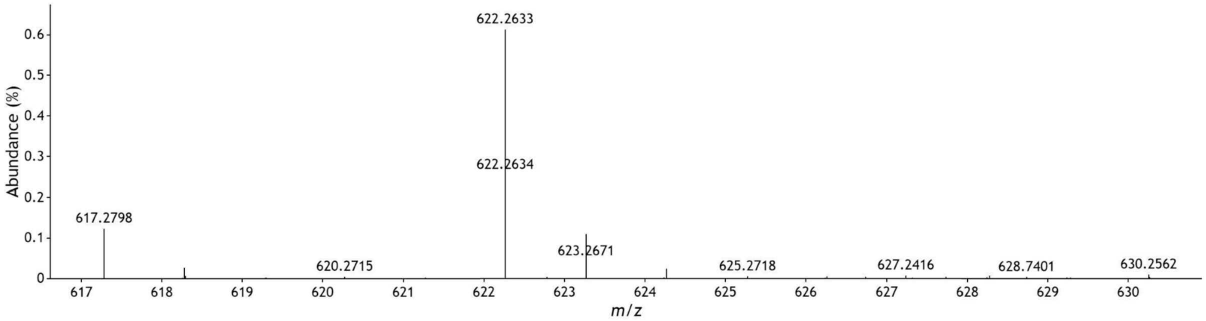

Theoretical Spectrum

C33H37N5O6 [M+Na]+

BP: 622.2636

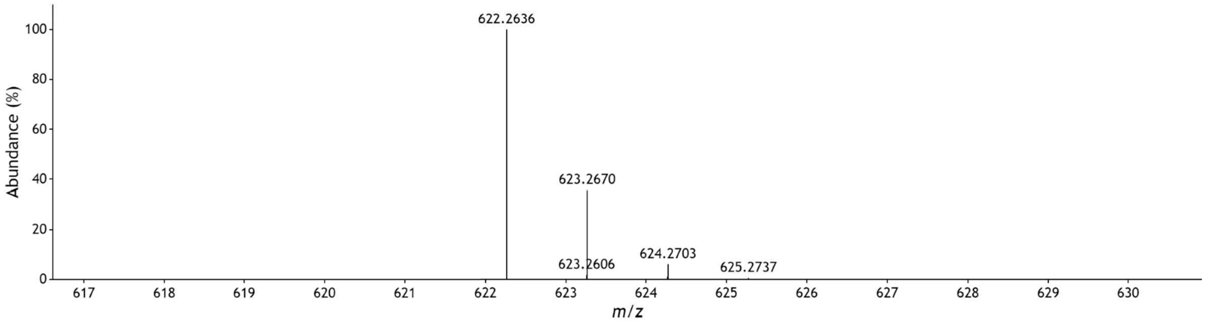

ge Structure

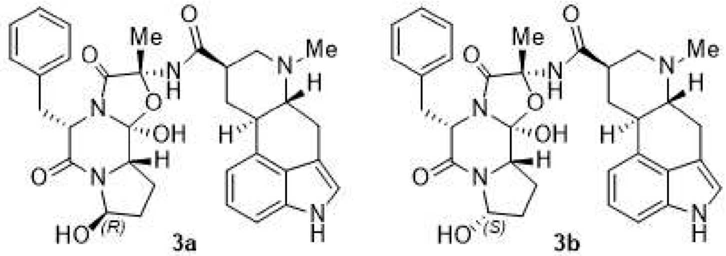

3a

3b

Chemical Formula: C33H37N5O6 Exact Mass: 599.27

Target 8-OH-DHE | C33H37N5O6 [M+Na]+ | Calculated *m/z* 622.2636 | Confirmation Trace - Base Peak

	Found RT	Area	Height	Area %	Total %	Target Peak	Measured *m/z*	Error (mmu)	Error (ppm)
1	3.6262	1,851,343,893.29	421,278,300.00	40.15	28.65	B	622.2633	-0.30	-0.49
2	3.8287	4,610,857,101.59	784,122,436.00	100.00	71.35	A	622.2633	-0.30	-0.49

ge Result - 8-OH-DHE

8OH-DHE - 8-OH-DHE, *m/z* 599.2738 ± 0.00250, FTMS + c ESI Full ms [100.0000-1500.0000]

NL: 4.039E05

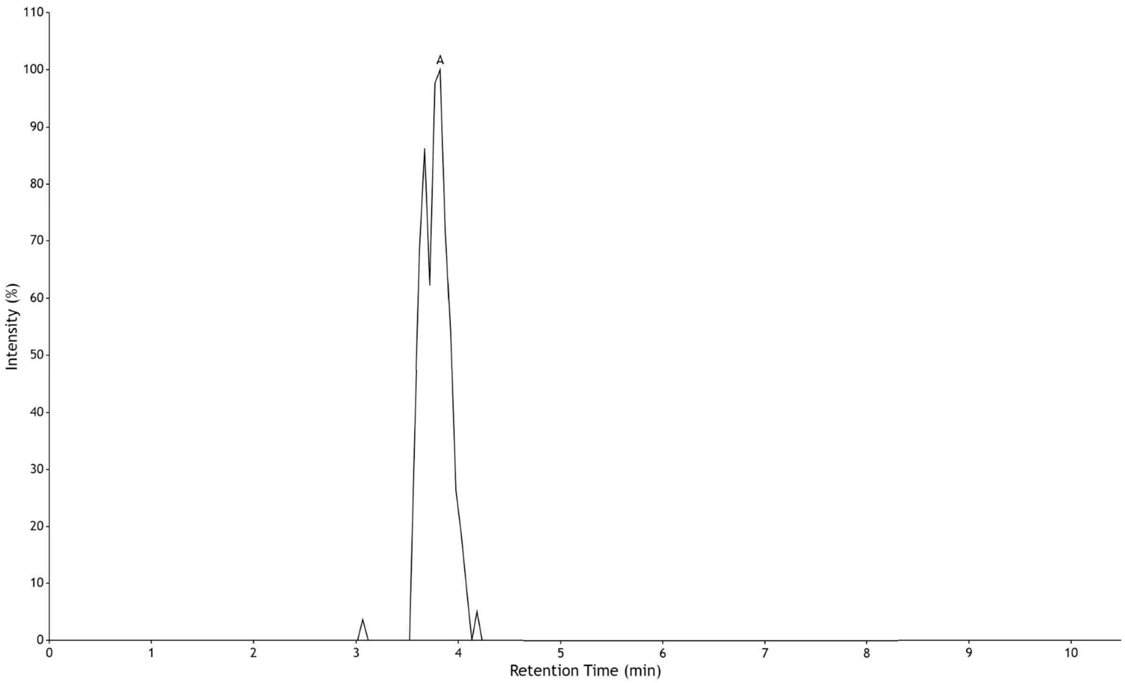

8OH-DHE, Base Peak, FTMS + c ESI Full ms [100.0000-1500.0000]

NL: 8.445E08

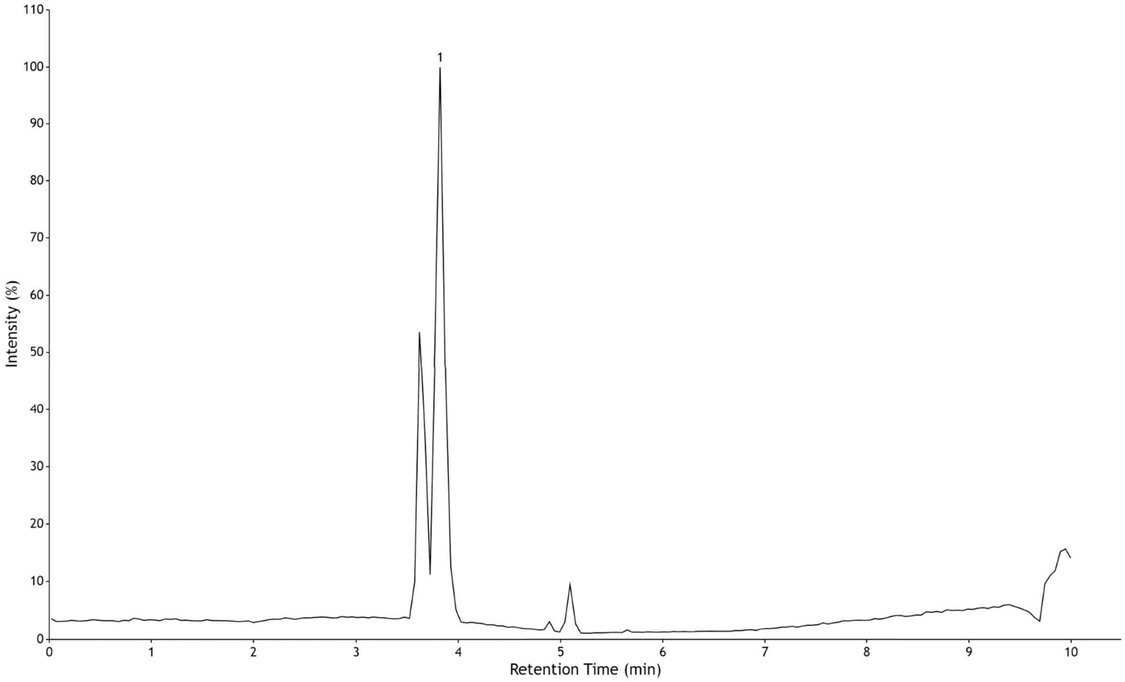

Page 10 of 16

e Peak A Average Spectrum

8-OH-DHE

RT: 3.8287, NL: 2.378E08, BP: 600.2811, Match Score: 11.09

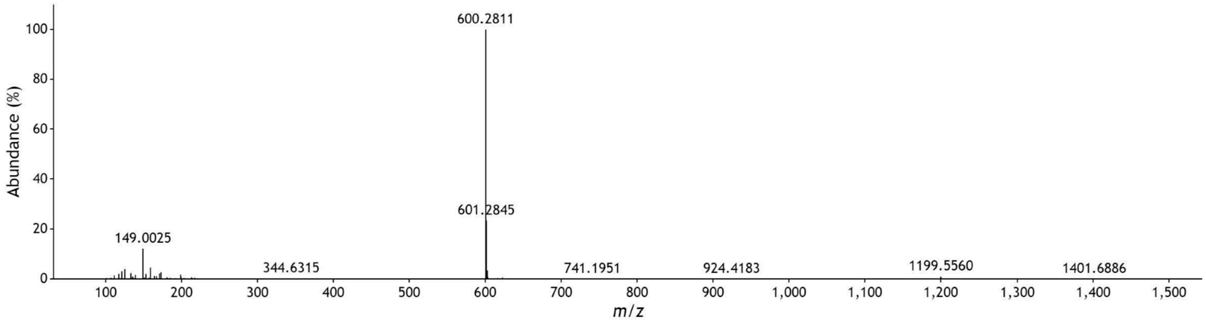

Expanded Target Peak A Average Spectrum

8-OH-DHE

RT: 3.8287, NL: 2.378E08, BP: 600.2811, Match Score: 11.09

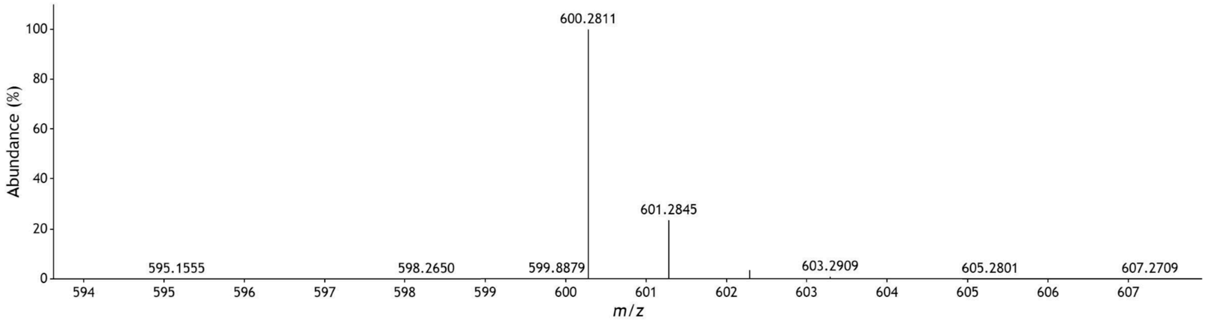

Theoretical Spectrum

C33H37N5O6 [M]+

BP: 599.2738

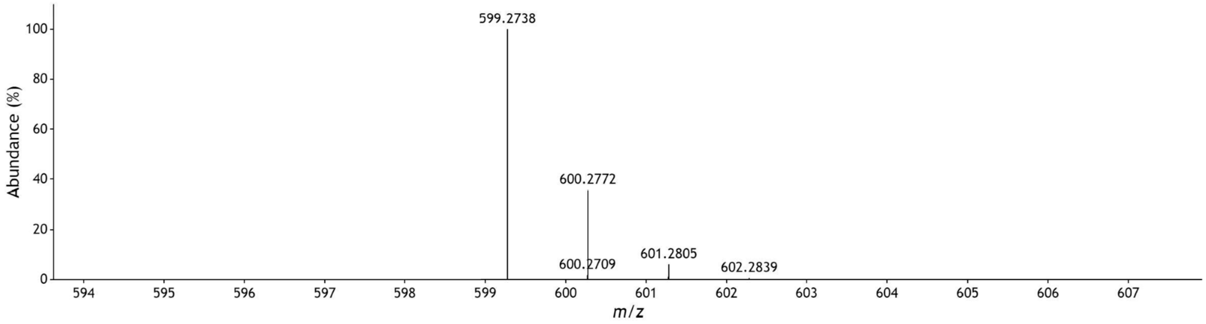

ge Structure

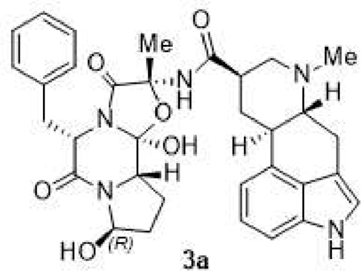

3a

Chemical Formula: C33H37N5O6 Exact Mass: 599.27

Target 8-OH-DHE | C33H37N5O6 [M]+ | Calculated *m/z* 599.2738 | Confirmation Trace - Base Peak

	Found RT	Area	Height	Area %	Total %	Target Peak	Measured *m/z*	Error (mmu)	Error (ppm)
1	3.8287	4,610,857,101.59	784,122,436.00	100.00	100.00	A	599.2728	-1.04	-1.73

e Result - 8-OH-DHE

8OH-DHE - 8-OH-DHE, *m/z* 598.2671 ± 0.00250, FTMS - c ESI Full ms [100.0000-1500.0000]

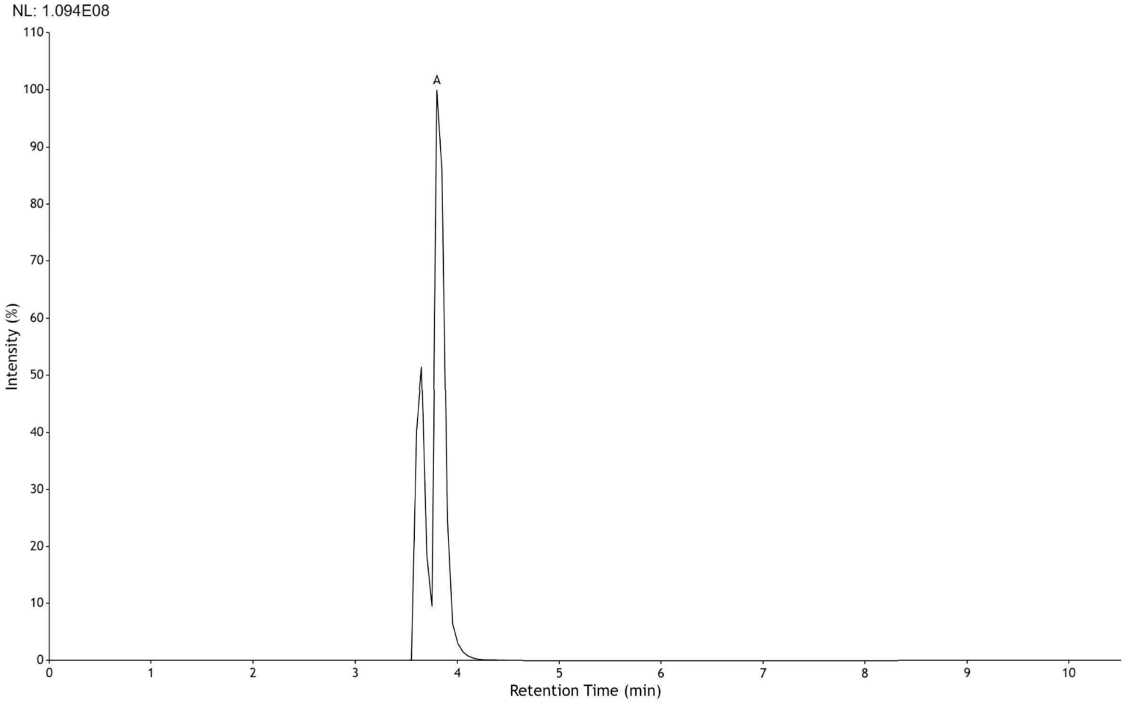

8OH-DHE, Base Peak, FTMS - c ESI Full ms [100.0000-1500.0000]

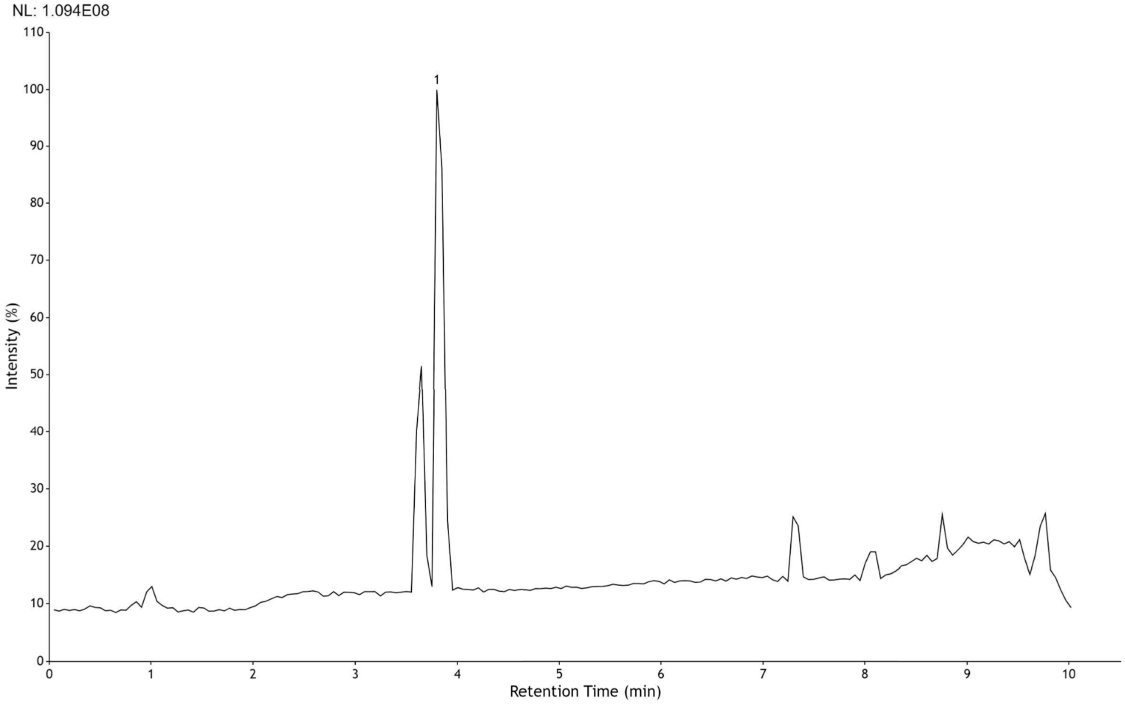

Page 13 of 16

e Peak A Average Spectrum

8-OH-DHE

RT: 3.8035, NL: 2.032E07, BP: 598.2670, Match Score: 99.74

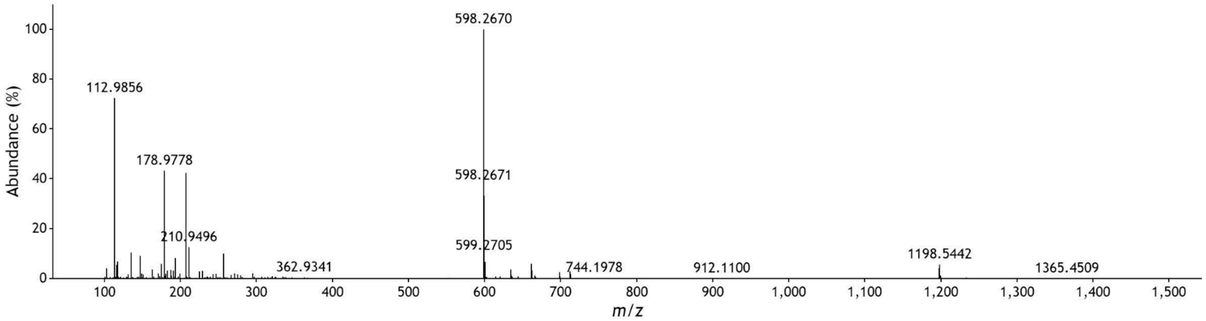

Expanded Target Peak A Average Spectrum

8-OH-DHE

RT: 3.8035, NL: 2.032E07, BP: 598.2670, Match Score: 99.74

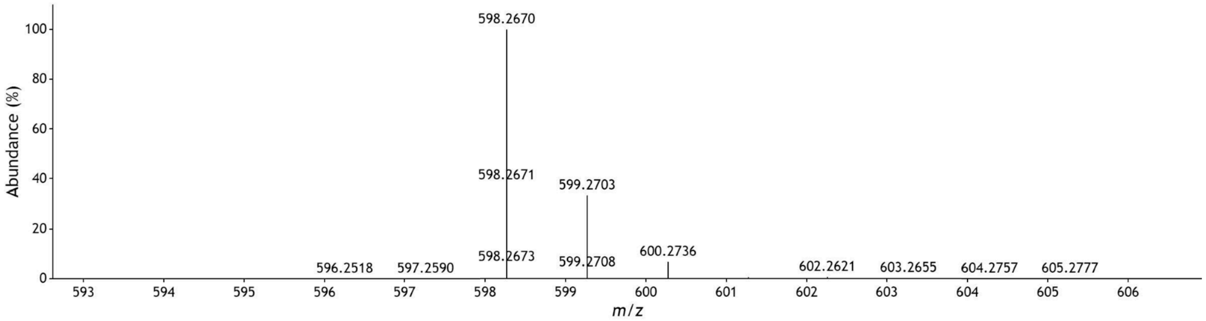

Theoretical Spectrum

C33H37N5O6 [M-H]-

BP: 598.2671

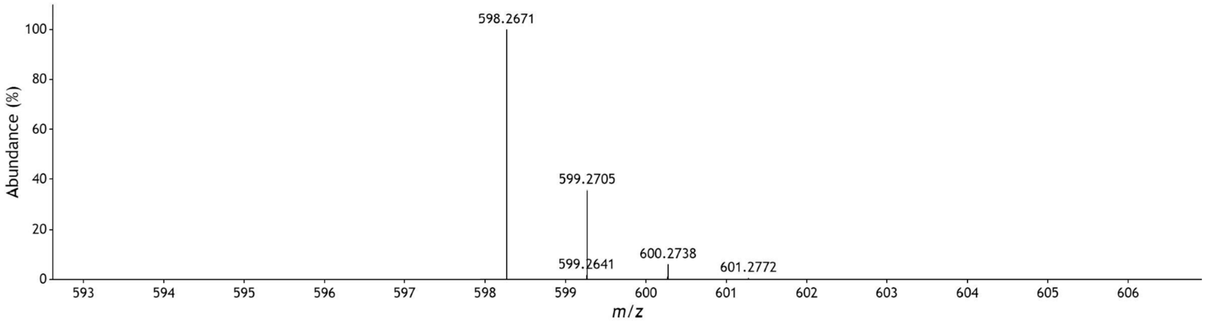

Sample Reference: 8-OH-DHE-d5 (2)
Submitted By: Alex Charlton
Data File: 8OH-DHE-d5.swrx
Experiment: HR-MS 1: Small Molecules (Polar) - Electrospray Ionisation (ESI; 100-1500 Da)

Target Confirmation Summary

	Name	Formula	Adduct	Calculated *m/z*	Measured *m/z*	Response	Found RT	Error (mmu)	Error (ppm)	Target Status	Within Error Limits
1	8-OH-DHE-d5	C33H32D5N5O6	[M+H]+	605.3130	605.3125	250,064,632.69	3.6184	-0.55	-0.90	Found	Yes
2	8-OH-DHE-d5	C33H32D5N5O6	[M+H]+	605.3130	605.3125	1,331,073,163.27	3.8212	-0.55	-0.90	Found	Yes
3	8-OH-DHE-d5	C33H32D5N5O6	[M+Na]+	627.2950	627.2950	3,838,174.87	3.6184	0.01	0.02	Found	Yes
4	8-OH-DHE-d5	C33H32D5N5O6	[M+Na]+	627.2950	627.2950	13,618,229.15	3.8212	0.01	0.02	Found	Yes
5	8-OH-DHE-d5	C33H32D5N5O6	[M+NH4]+	622.3396	0.0000	0.00	0.0000			Not Found	
6	8-OH-DHE-d5	C33H32D5N5O6	[M]+	604.3052	604.3066	8,137,845.10	3.6184	1.38	2.28	Found	Yes
7	8-OH-DHE-d5	C33H32D5N5O6	[M]+	604.3052	604.3066	52,501,143.47	3.8212	1.38	2.28	Found	Yes
8	8-OH-DHE-d5	C33H32D5N5O6	[M-H]-	603.2985	603.2985	52,153,991.79	3.6437	0.01	0.01	Found	Yes
9	8-OH-DHE-d5	C33H32D5N5O6	[M-H]-	603.2985	603.2985	156,633,772.67	3.7960	0.01	0.01	Found	Yes

An error of < 5 ppm indicates that the measured mass is consistent with the proposed formula.

ge Result - 8-OH-DHE-d5

8OH-DHE-d5 - 8-OH-DHE-d5, *m/z* 605.3130 ± 0.00250, FTMS + c ESI Full ms [100.0000-1500.0000]

NL: 2.466E08

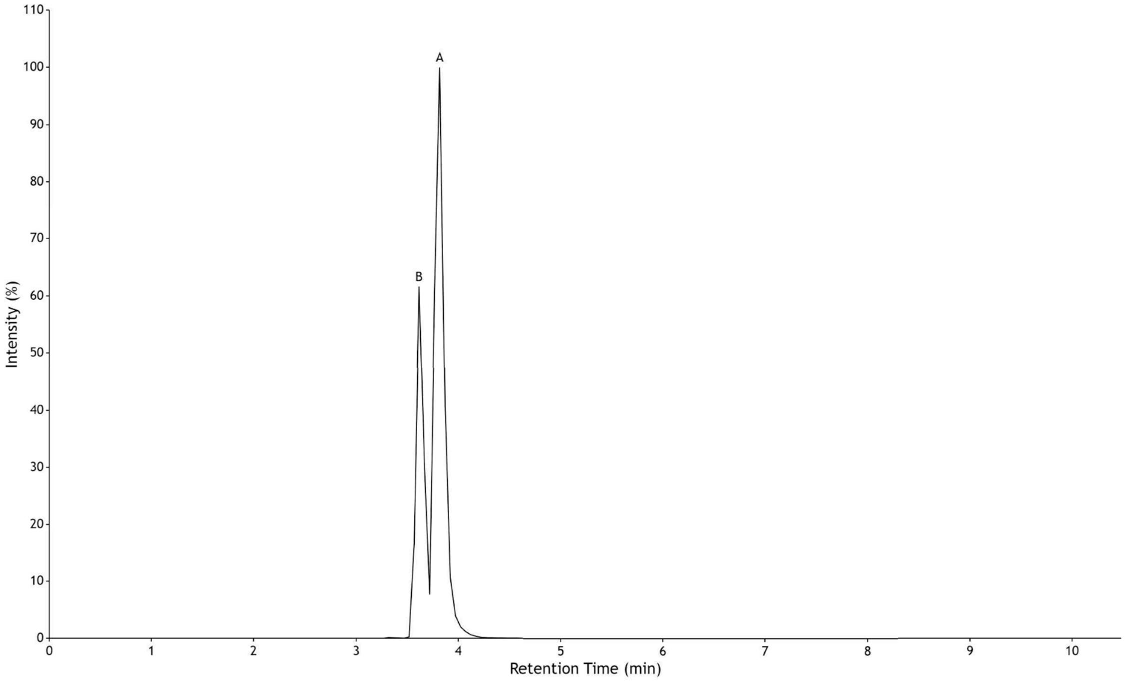

8OH-DHE-d5, Base Peak, FTMS + c ESI Full ms [100.0000-1500.0000]

NL: 2.466E08

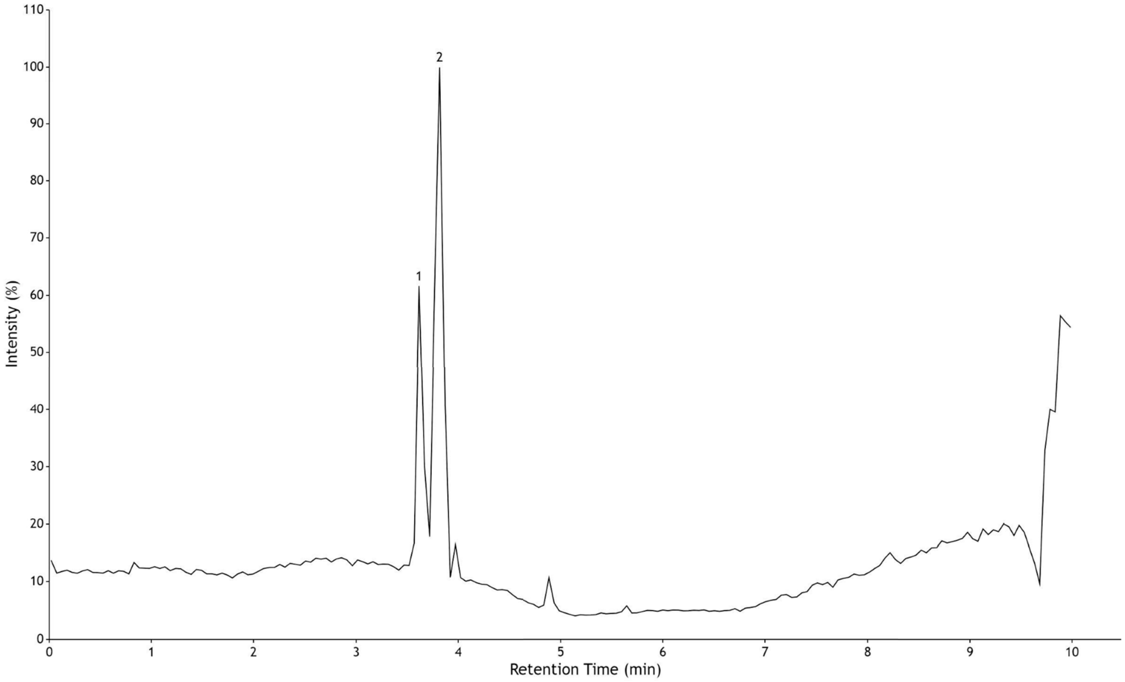

Page 2 of 18

ge Peak A Average Spectrum

8-OH-DHE-d5

RT: 3.8212, NL: 4.975E07, BP: 605.3125, Match Score: 18.47

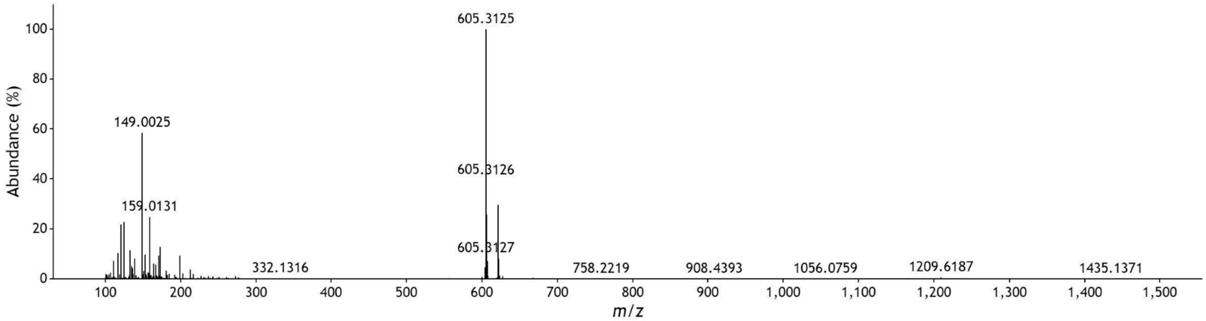

Expanded Target Peak A Average Spectrum

8-OH-DHE-d5

RT: 3.8212, NL: 4.975E07, BP: 605.3125, Match Score: 18.47

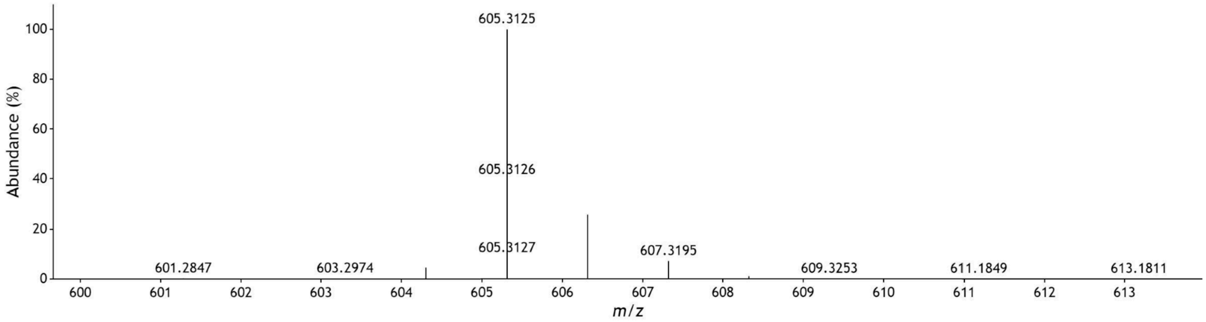

Theoretical Spectrum

C33H32D5N5O6 [M+H]+

BP: 605.3130

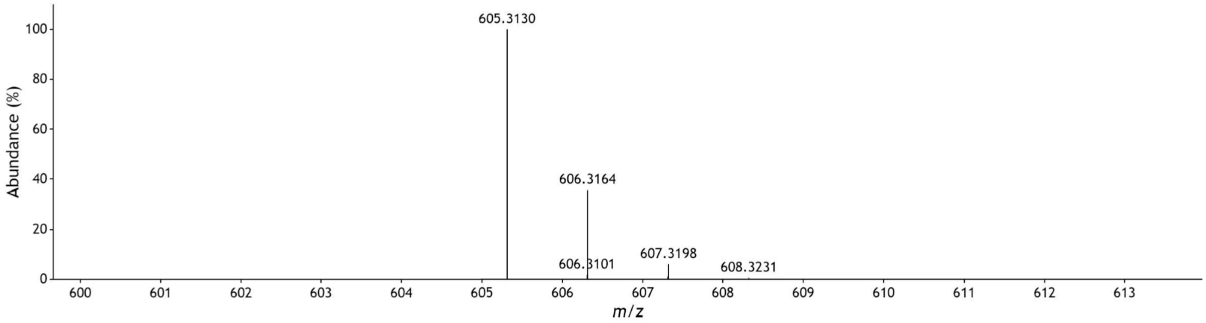

ge Peak B Average Spectrum

8-OH-DHE-d5
RT: 3.6184, NL: 4.975E07, BP: 605.3125, Match Score: 2.36

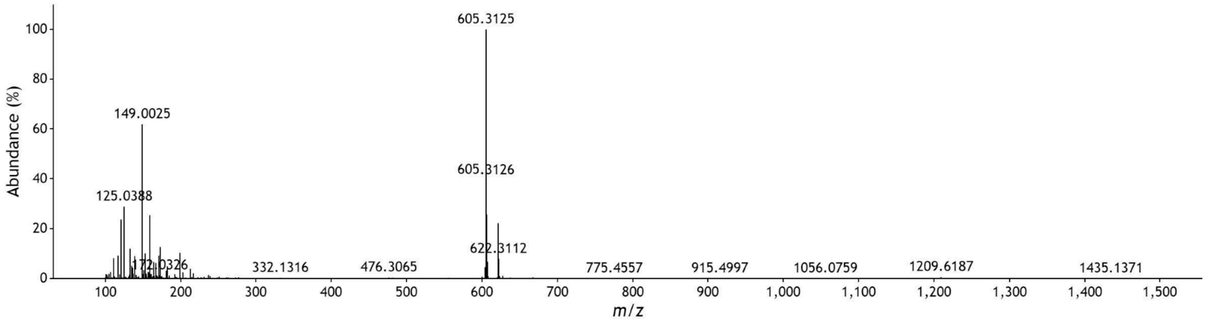

Expanded Target Peak B Average Spectrum

8-OH-DHE-d5
RT: 3.6184, NL: 4.975E07, BP: 605.3125, Match Score: 2.36

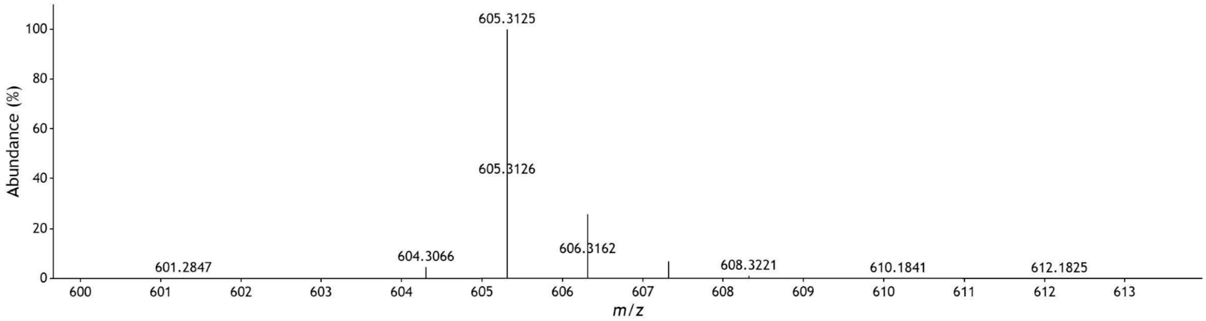

Theoretical Spectrum

C33H32D5N5O6 [M+H]+
BP: 605.3130

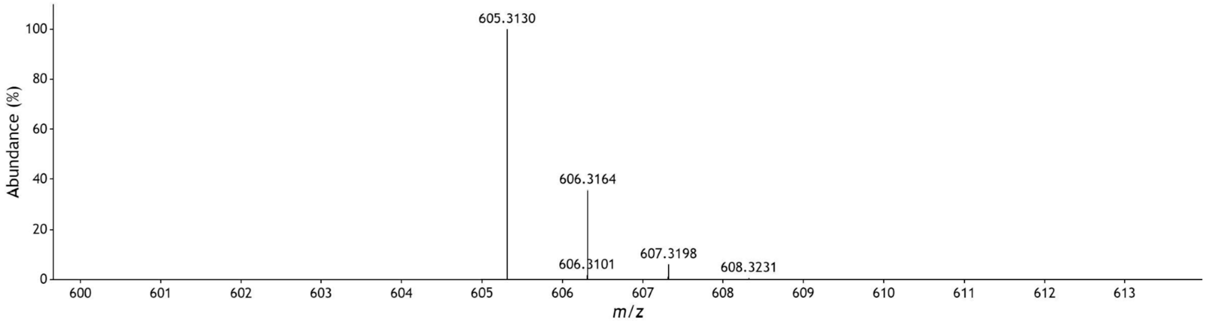

ge Structure

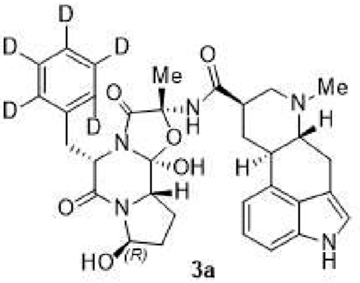

3a

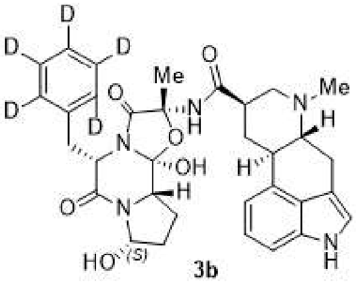

3b

Chemical Formula: C33H37N5O6 Exact Mass: 599.27

Target 8-OH-DHE-d5 | C33H32D5N5O6 [M+H]+ | Calculated *m/z* 605.3130 | Confirmation Trace - Base Peak

	Found RT	Area	Height	Area %	Total %	Target Peak	Measured *m/z*	Error (mmu)	Error (ppm)
1	3.6184	265,053,436.22	99,152,694.00	34.88	25.86	B	605.3125	-0.55	-0.90
2	3.8212	760,006,882.79	173,825,358.00	100.00	74.14	A	605.3125	-0.55	-0.90

ge Result - 8-OH-DHE-d5

8OH-DHE-d5 - 8-OH-DHE-d5, *m/z* 627.2950 ± 0.00250, FTMS + c ESI Full ms [100.0000-1500.0000]

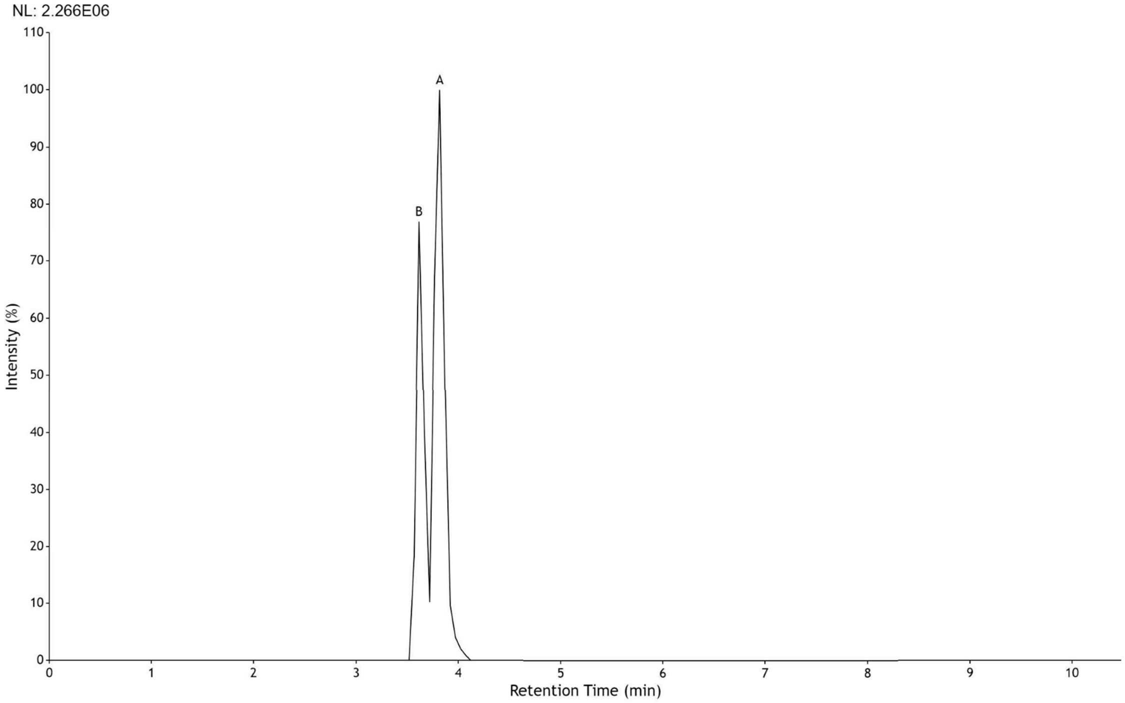

8OH-DHE-d5, Base Peak, FTMS + c ESI Full ms [100.0000-1500.0000]

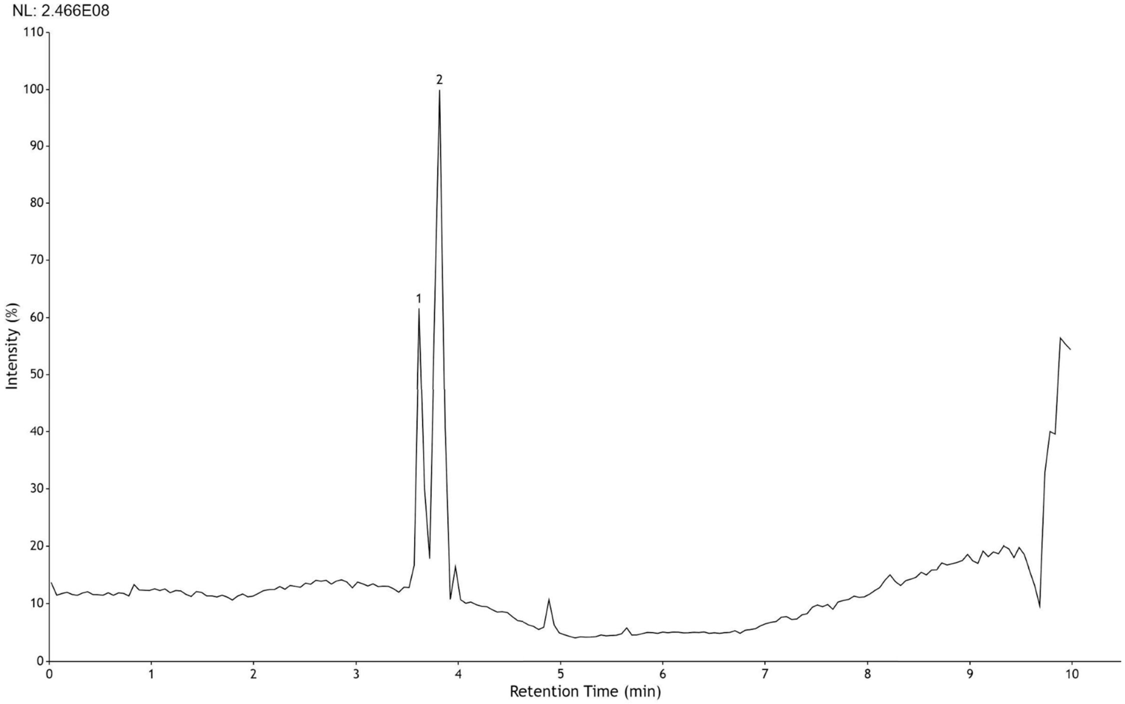

Page 6 of 18

ge Peak A Average Spectrum

8-OH-DHE-d5

RT: 3.8212, NL: 4.975E07, BP: 605.3125, Match Score: 99.75

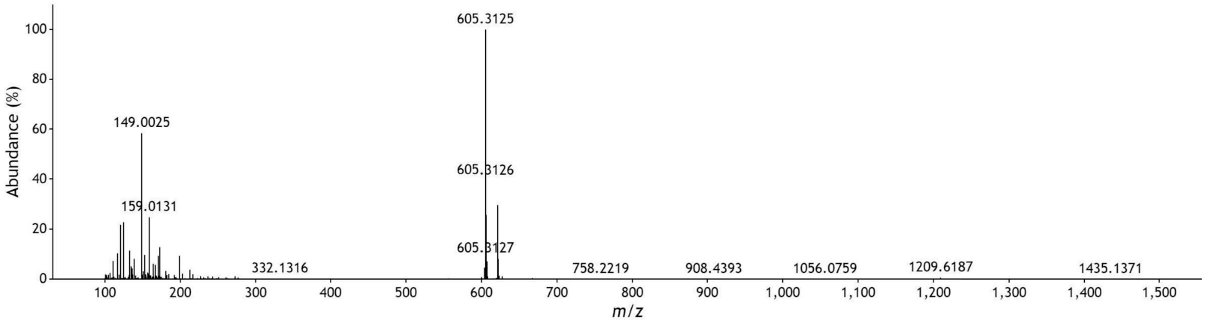

Expanded Target Peak A Average Spectrum

8-OH-DHE-d5

RT: 3.8212, NL: 4.975E07, BP: 605.3125, Match Score: 99.75

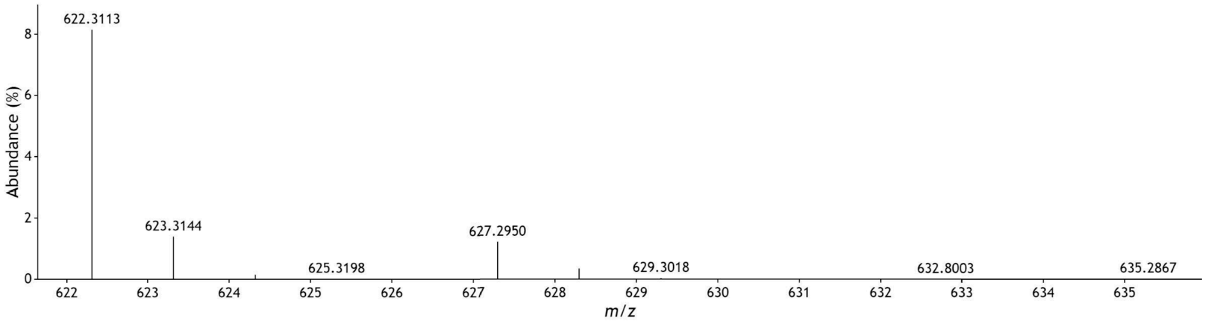

Theoretical Spectrum

C33H32D5N5O6 [M+Na]+

BP: 627.2950

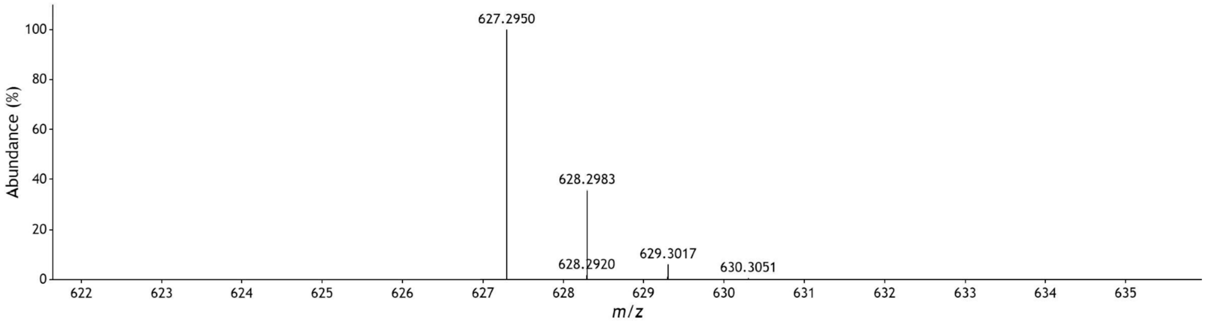

ge Peak B Average Spectrum

8-OH-DHE-d5

RT: 3.6184, NL: 4.975E07, BP: 605.3125, Match Score: 99.55

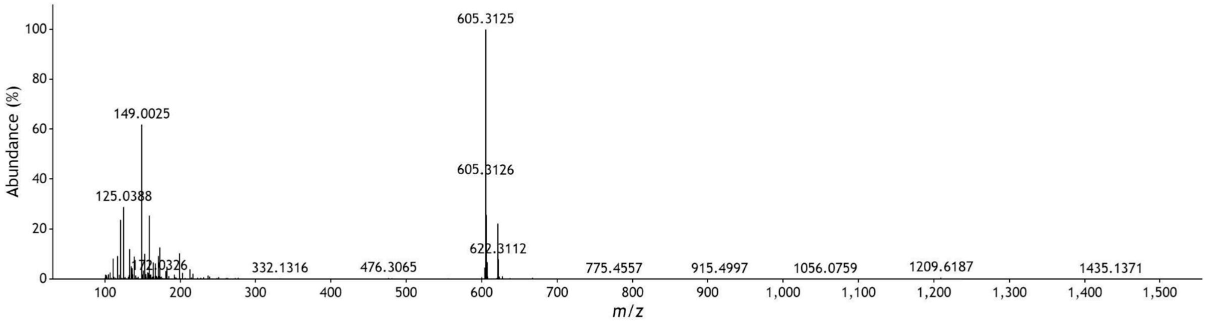

Expanded Target Peak B Average Spectrum

8-OH-DHE-d5

RT: 3.6184, NL: 4.975E07, BP: 605.3125, Match Score: 99.55

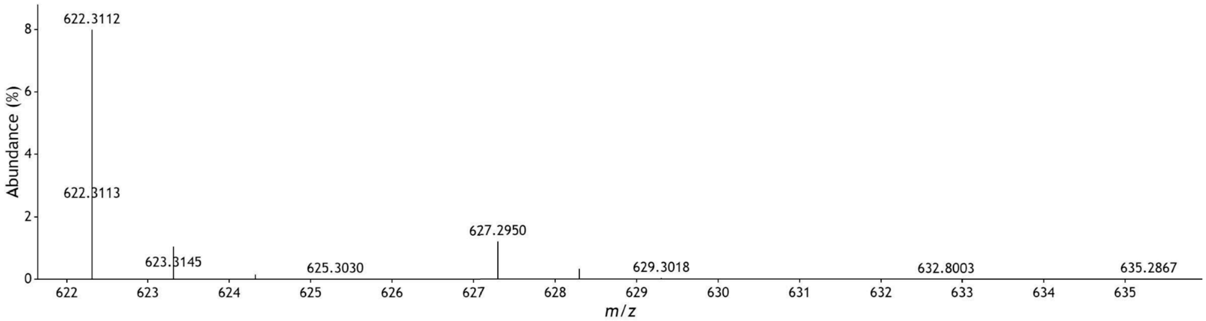

Theoretical Spectrum

C33H32D5N5O6 [M+Na]+

BP: 627.2950

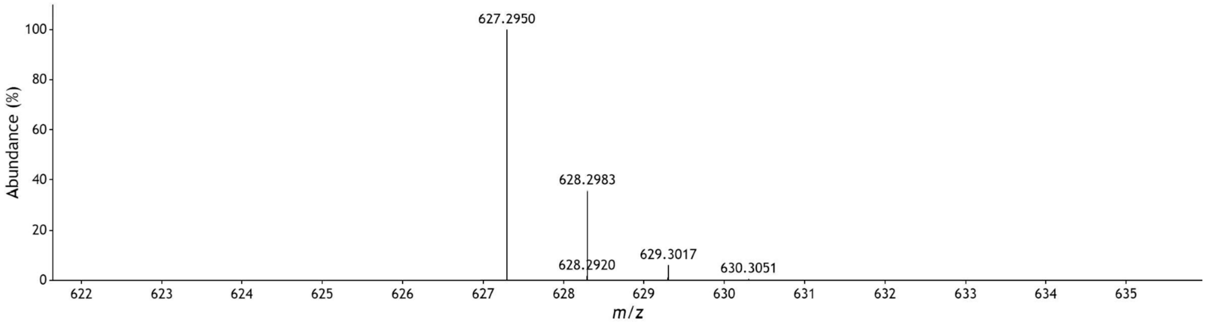

ge Structure

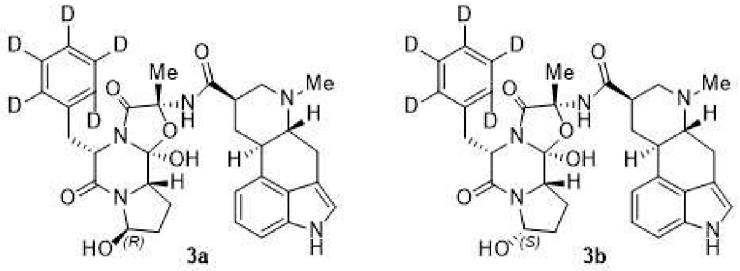

Chemical Formula: C33H37N5O6 Exact Mass: 599.27

Target 8-OH-DHE-d5 | C33H32D5N5O6 [M+Na]+ | Calculated *m/z* 627.2950 | Confirmation Trace - Base Peak

	Found RT	Area	Height	Area %	Total %	Target Peak	Measured *m/z*	Error (mmu)	Error (ppm)
1	3.6184	265,053,436.22	99,152,694.00	34.88	25.86	B	627.2950	0.01	0.02
2	3.8212	760,006,882.79	173,825,358.00	100.00	74.14	A	627.2950	0.01	0.02

ge Result - 8-OH-DHE-d5

8OH-DHE-d5 - 8-OH-DHE-d5, *m/z* 604.3052 ± 0.00250, FTMS + c ESI Full ms [100.0000-1500.0000]

NL: 9.329E06

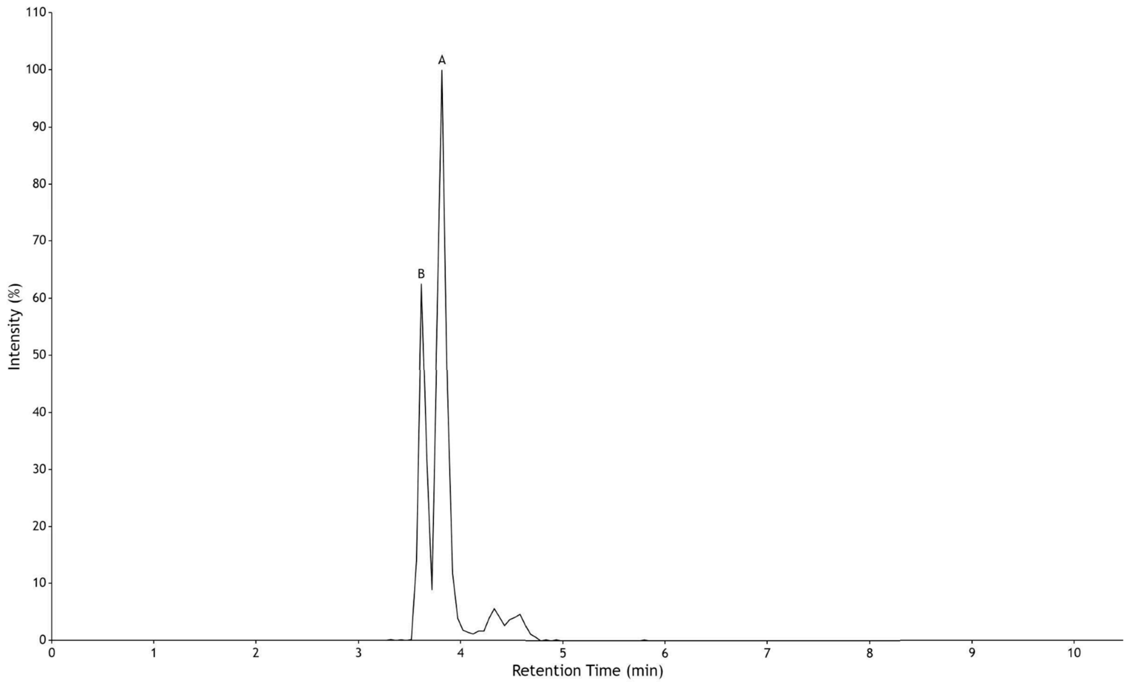

8OH-DHE-d5, Base Peak, FTMS + c ESI Full ms [100.0000-1500.0000]

NL: 2.466E08

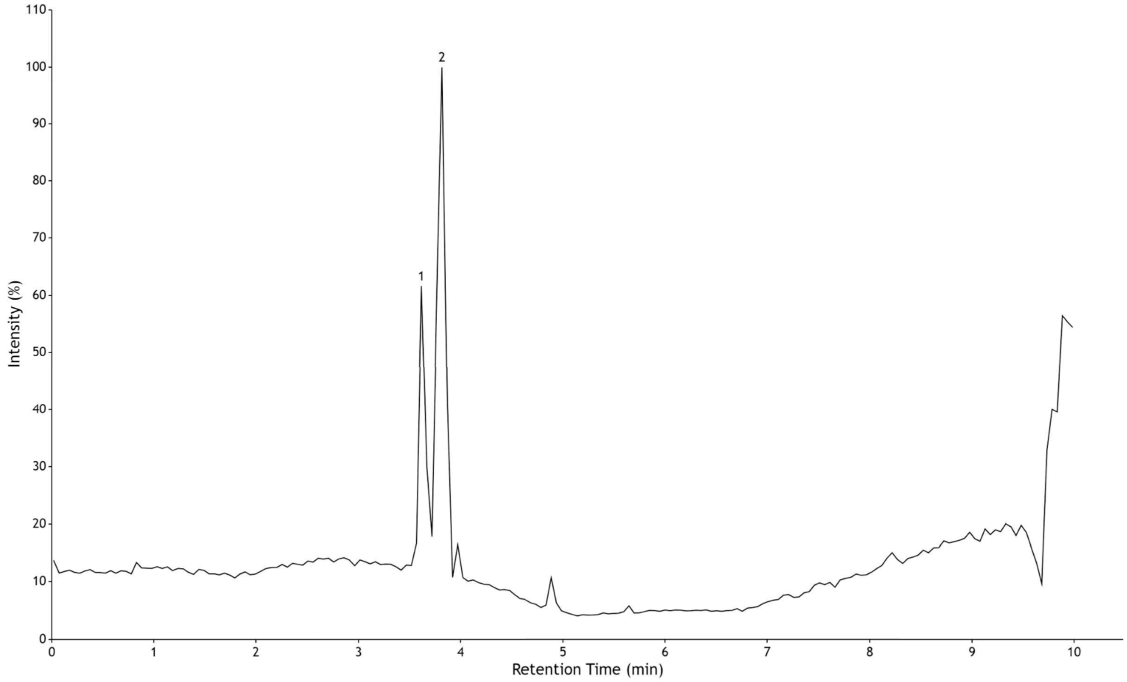

Page 10 of 18

e Peak A Average Spectrum

8-OH-DHE-d5

RT: 3.8212, NL: 4.975E07, BP: 605.3125, Match Score: 50.76

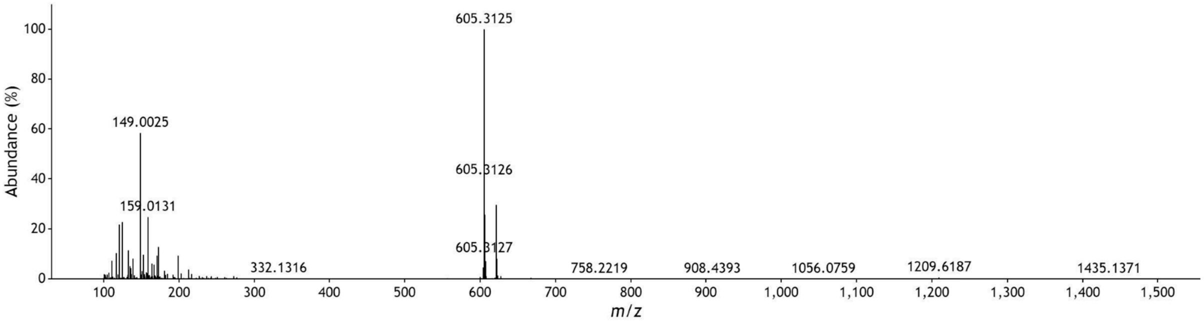

Expanded Target Peak A Average Spectrum

8-OH-DHE-d5

RT: 3.8212, NL: 4.975E07, BP: 605.3125, Match Score: 50.76

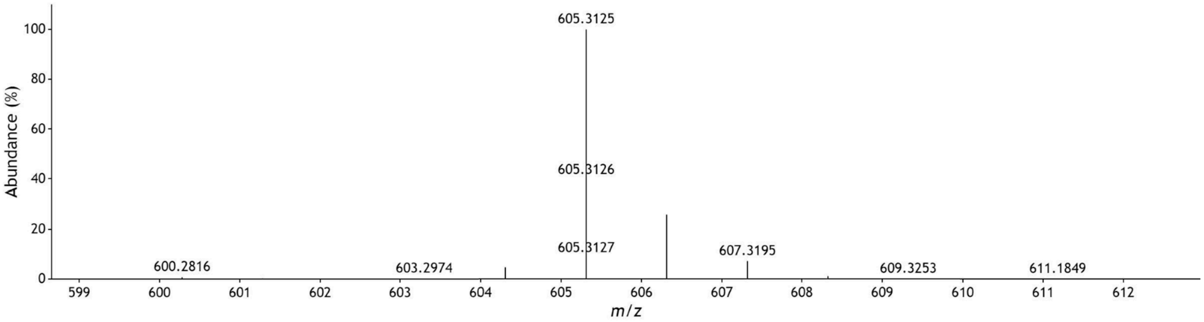

Theoretical Spectrum

C33H32D5N5O6 [M]+

BP: 604.3052

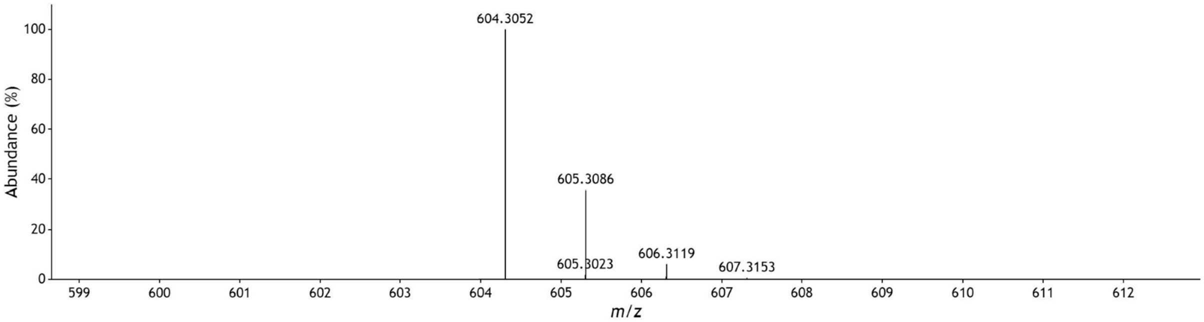

e Peak B Average Spectrum

8-OH-DHE-d5

RT: 3.6184, NL: 4.975E07, BP: 605.3125, Match Score: 1.18

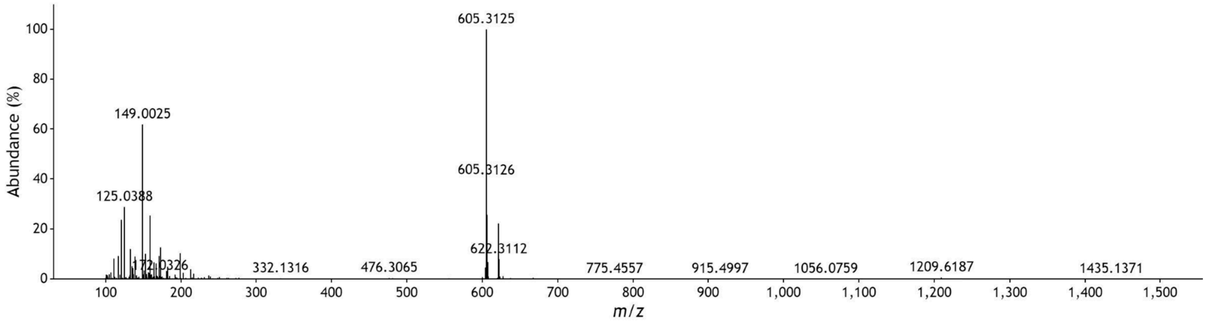

Expanded Target Peak B Average Spectrum

8-OH-DHE-d5

RT: 3.6184, NL: 4.975E07, BP: 605.3125, Match Score: 1.18

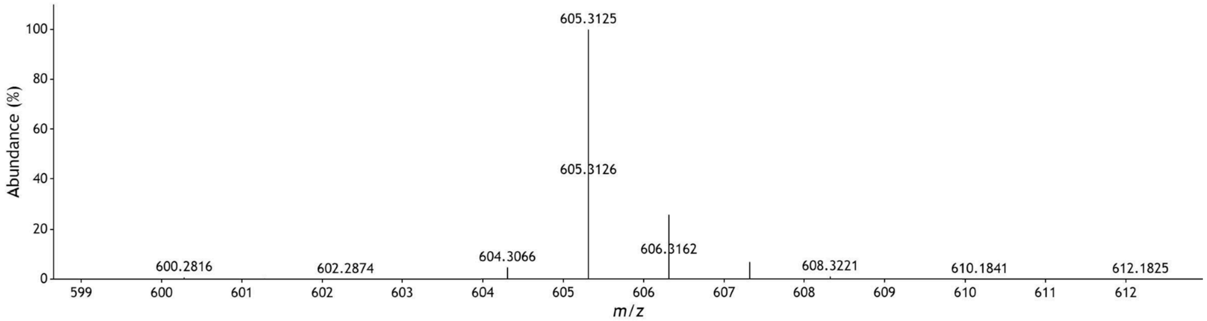

Theoretical Spectrum

C33H32D5N5O6 [M]+

BP: 604.3052

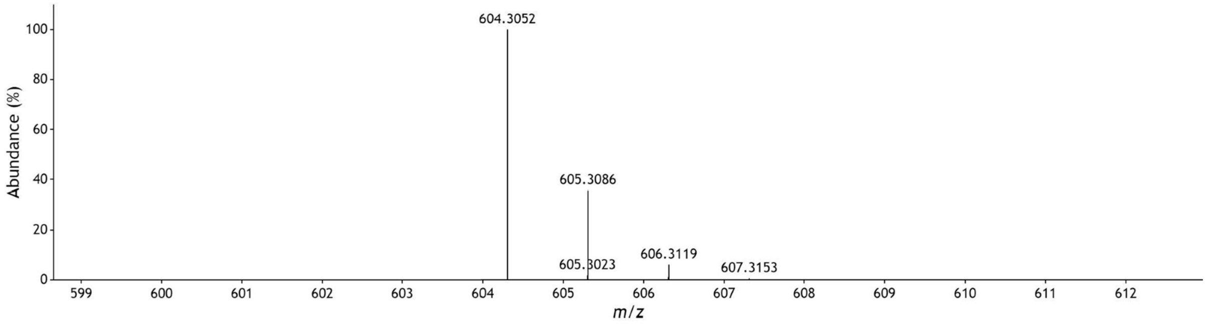

e Structure

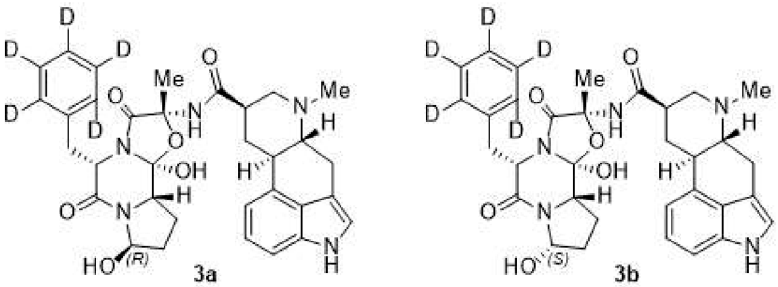

Chemical Formula: C33H37N5O6 Exact Mass: 599.27

Target 8-OH-DHE-d5 | C33H32D5N5O6 [M]+ | Calculated *m/z* 604.3052 | Confirmation Trace - Base Peak

	Found RT	Area	Height	Area %	Total %	Target Peak	Measured *m/z*	Error (mmu)	Error (ppm)
1	3.6184	265,053,436.22	99,152,694.00	34.88	25.86	B	604.3066	1.38	2.28
2	3.8212	760,006,882.79	173,825,358.00	100.00	74.14	A	604.3066	1.38	2.28

e Result - 8-OH-DHE-d5

8OH-DHE-d5 - 8-OH-DHE-d5, *m/z* 603.2985 ± 0.00250, FTMS - c ESI Full ms [100.0000-1500.0000]

NL: 3.392E07

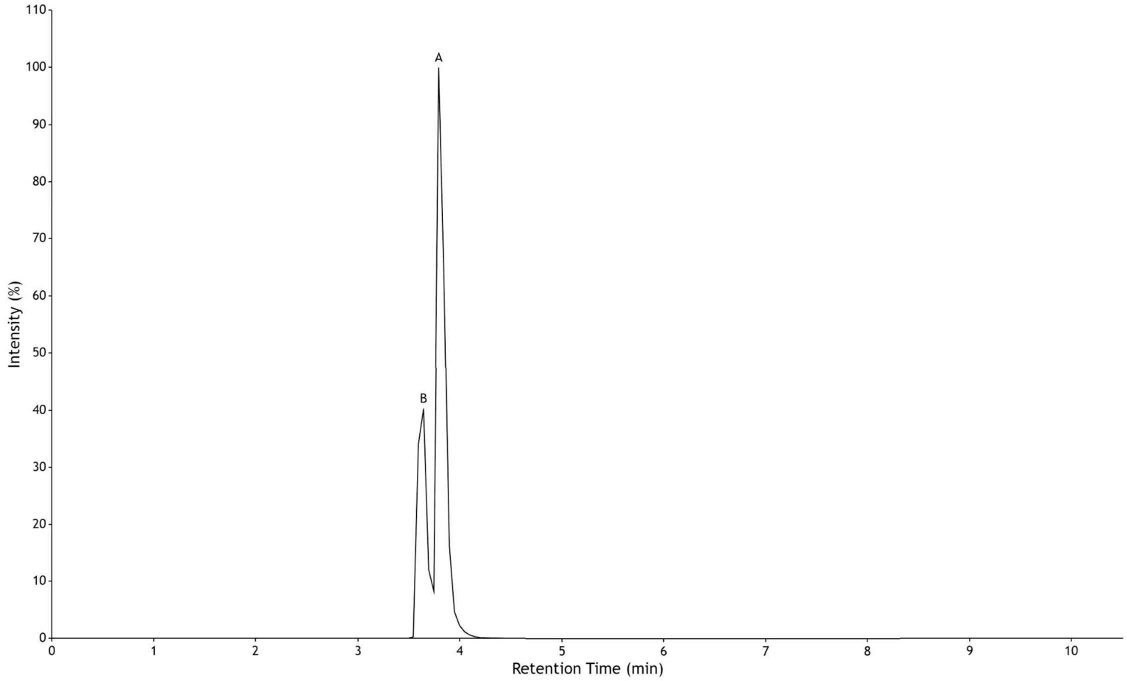

8OH-DHE-d5, Base Peak, FTMS - c ESI Full ms [100.0000-1500.0000]

NL: 3.392E07

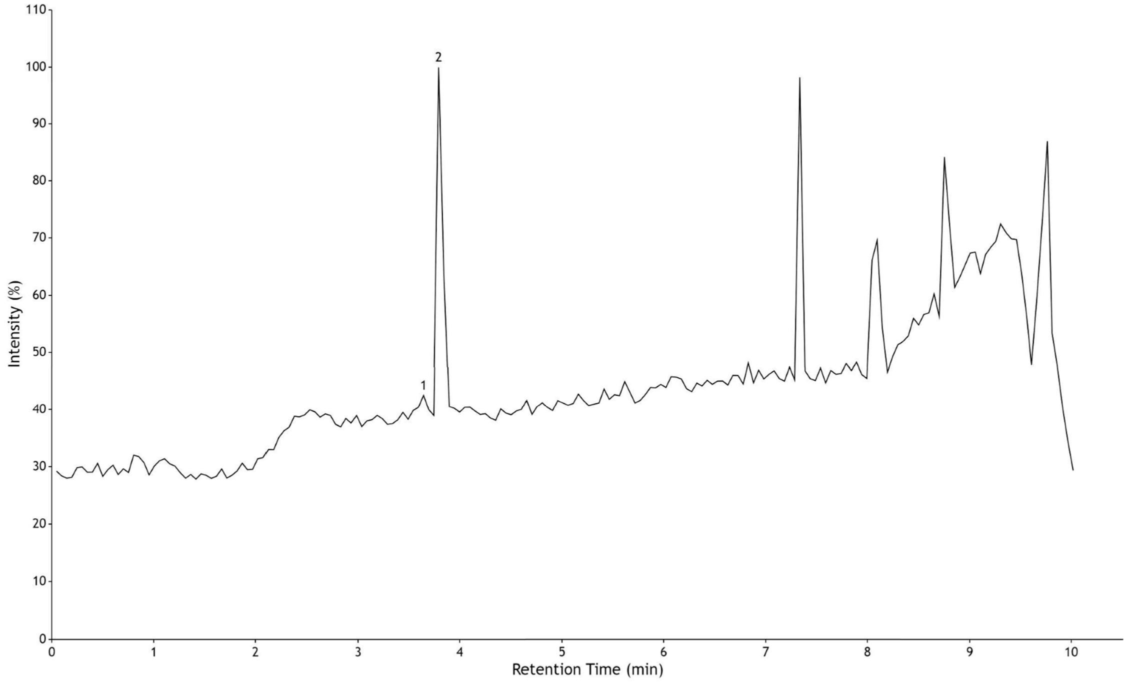

Page 14 of 18

e Peak A Average Spectrum

8-OH-DHE-d5

RT: 3.7960, NL: 1.39E07, BP: 112.9856, Match Score: 98.67

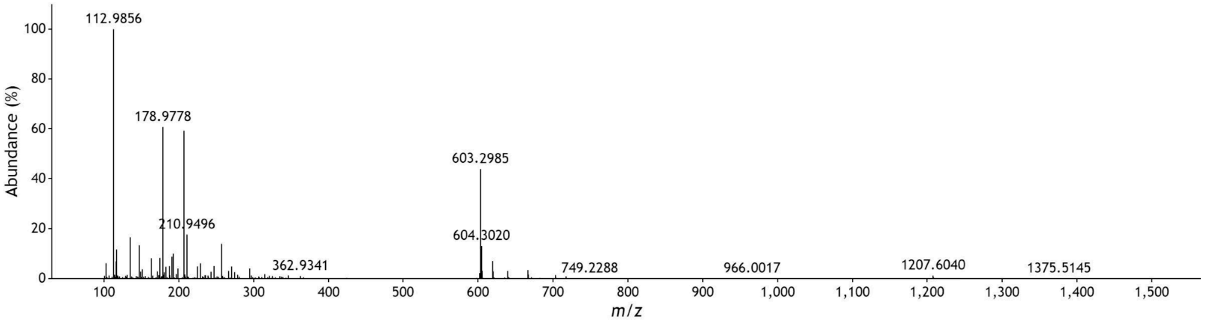

Expanded Target Peak A Average Spectrum

8-OH-DHE-d5

RT: 3.7960, NL: 1.39E07, BP: 112.9856, Match Score: 98.67

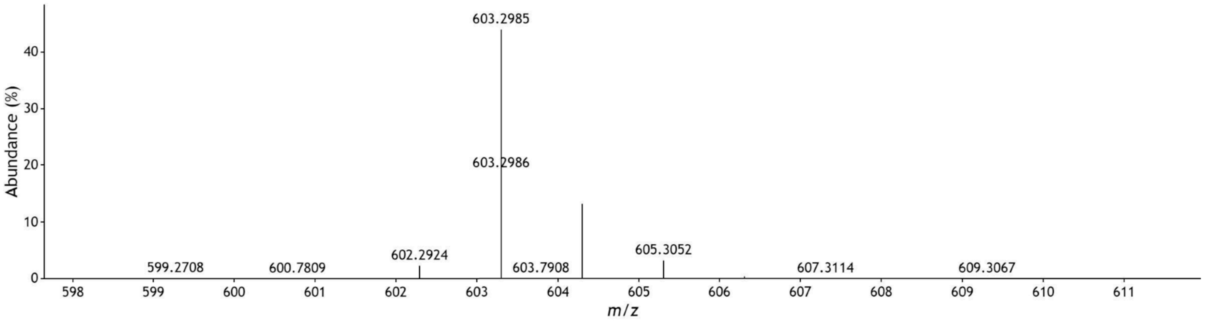

Theoretical Spectrum

C33H32D5N5O6 [M-H]-

BP: 603.2985

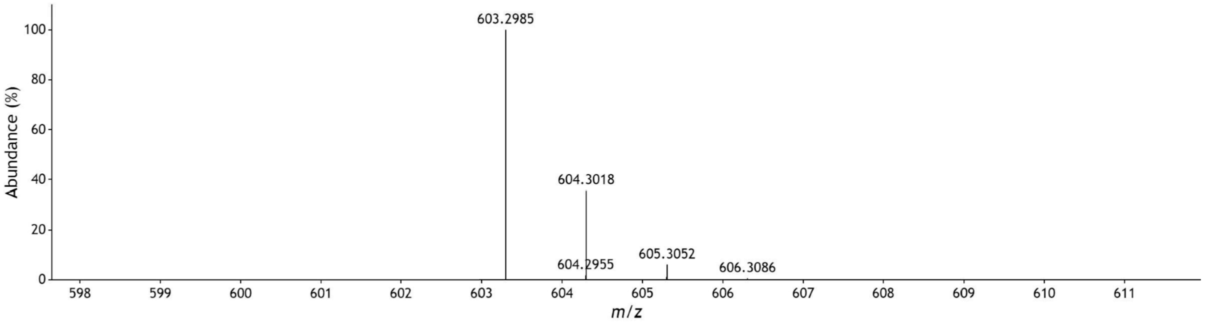

e Peak B Average Spectrum

8-OH-DHE-d5

RT: 3.6437, NL: 1.377E07, BP: 112.9856, Match Score: 98.72

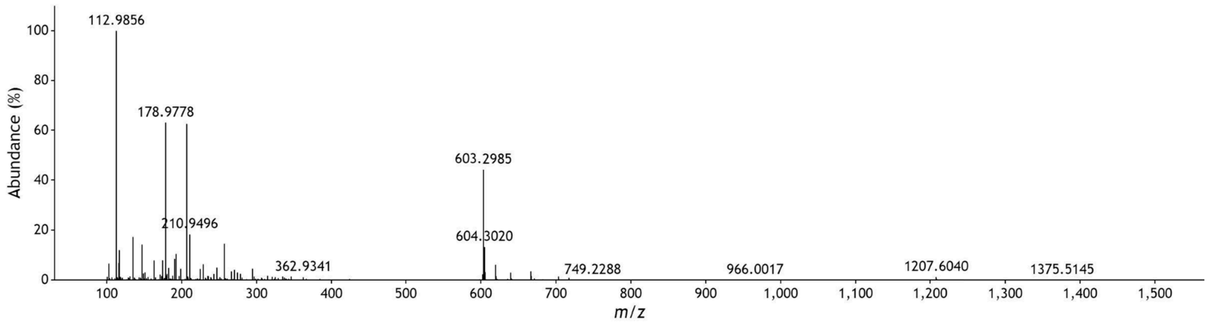

Expanded Target Peak B Average Spectrum

8-OH-DHE-d5

RT: 3.6437, NL: 1.377E07, BP: 112.9856, Match Score: 98.72

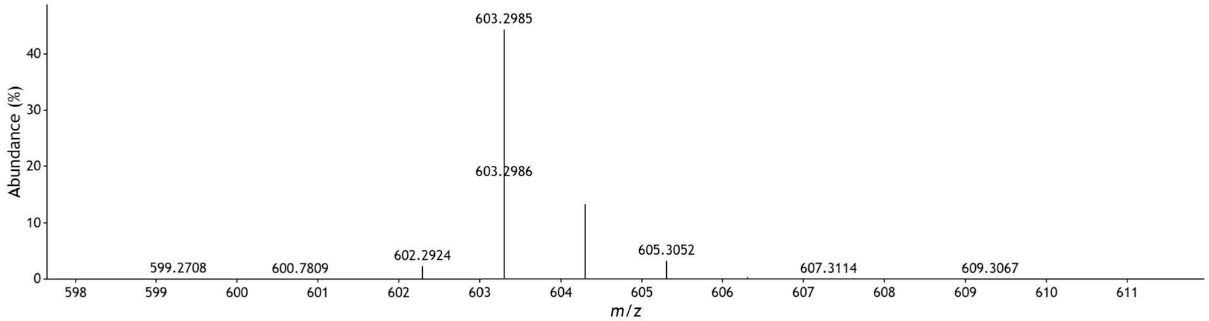

Theoretical Spectrum

C33H32D5N5O6 [M-H]-

BP: 603.2985

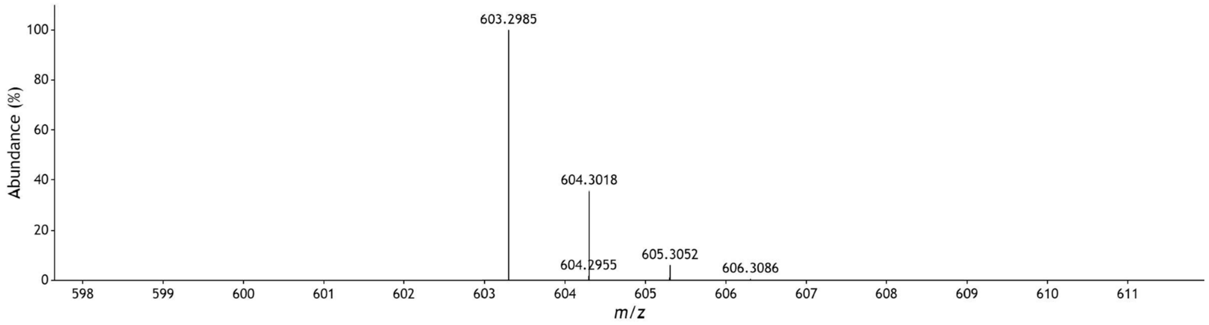

e Structure

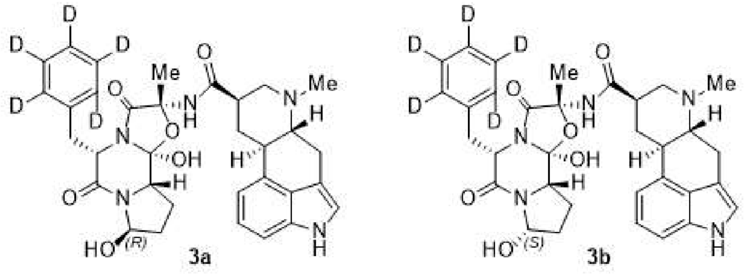

Chemical Formula: $C_{33}H_{37}N_5O_6$ Exact Mass: 599.27

Target 8-OH-DHE-d5 | $C_{33}H_{32}D_5N_5O_6$ [M-H]- | Calculated *m/z* 603.2985 | Confirmation Trace - Base Peak

	Found RT	Area	Height	Area %	Total %	Target Peak	Measured *m/z*	Error (mmu)	Error (ppm)
1	3.6437	5,496,283.26	1,154,290.00	6.23	5.86	B	603.2985	0.01	0.01
2	3.7960	88,278,801.91	20,487,165.00	100.00	94.14	A	603.2985	0.01	0.01

2. NMR Spectra

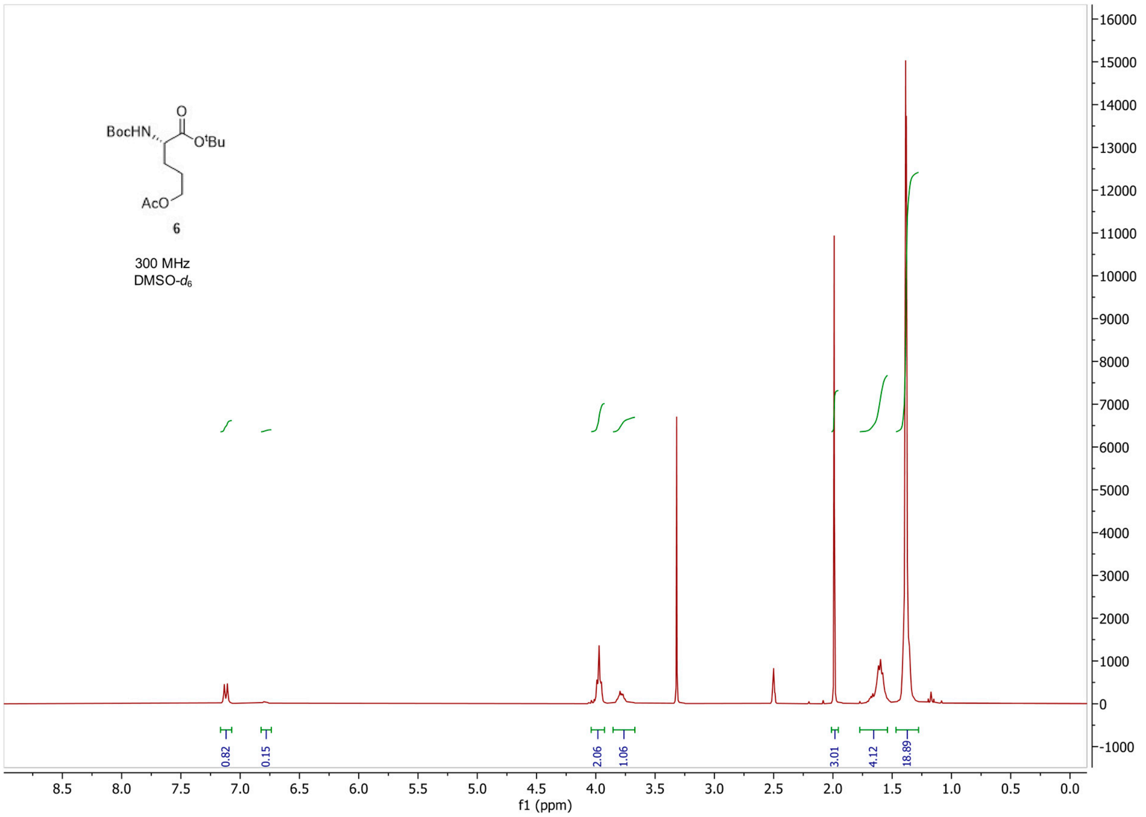

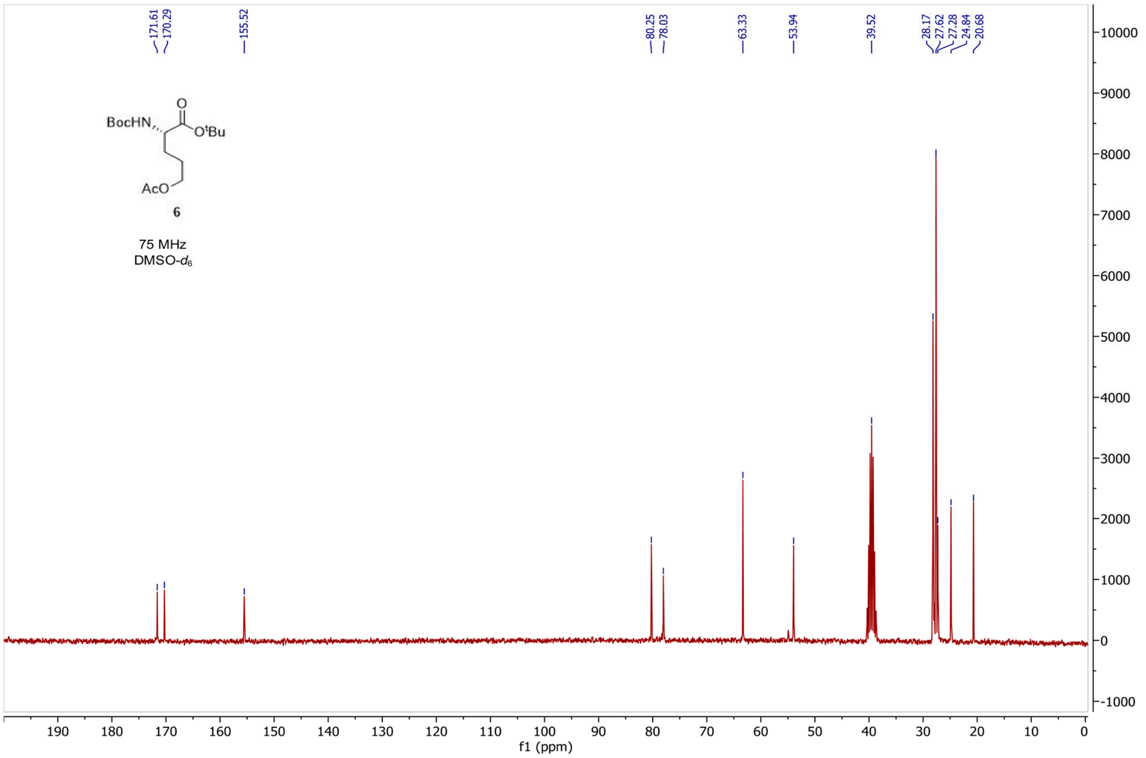

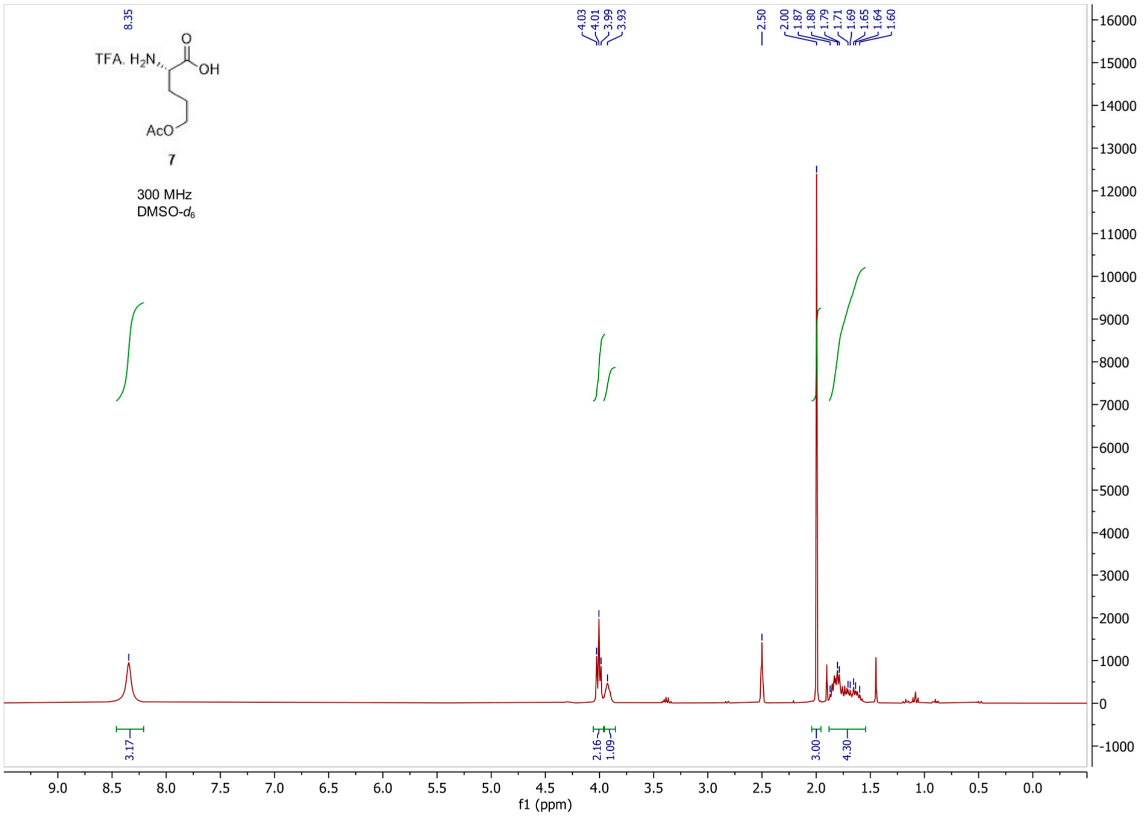

8.35
4.03
4.01
3.99
3.93
2.50
2.00
1.87
1.80
1.79
1.71
1.69
1.65
1.64
1.60
TFA. H2N
O
OH
AcO
7
300 MHz
DMSO-d6
3.17
2.16
1.09
3.00
4.30
f1 (ppm)

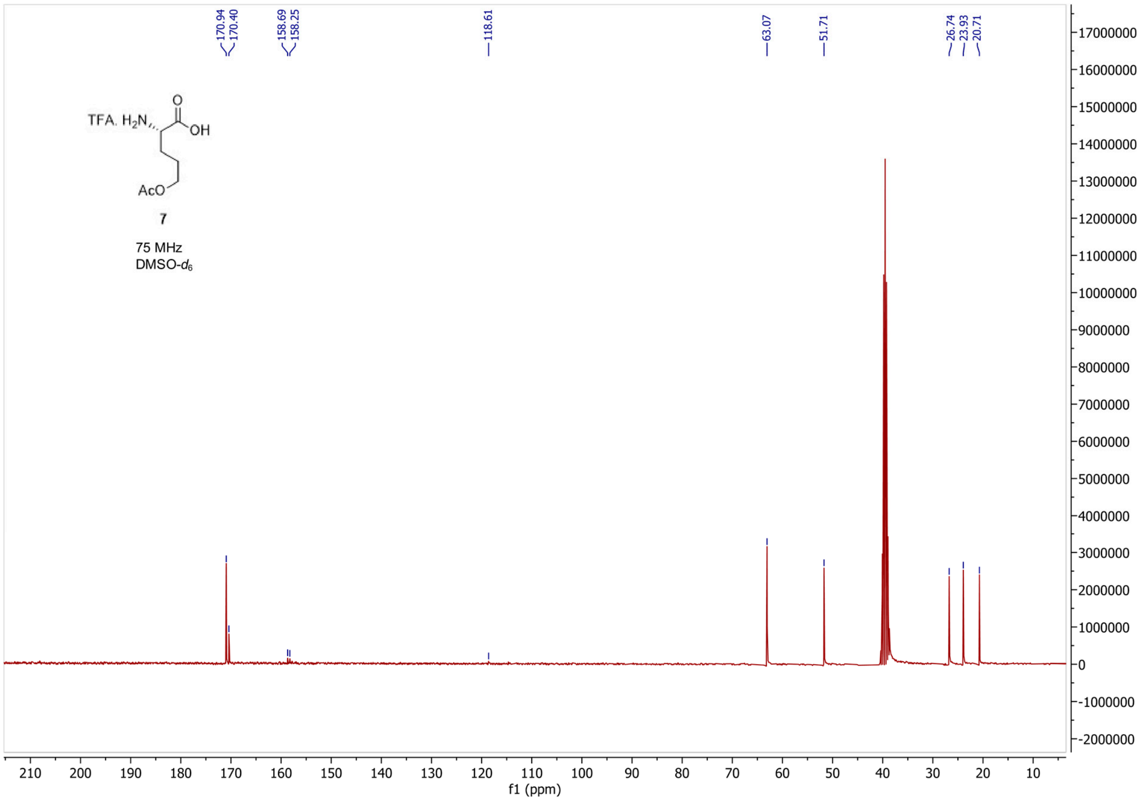

170.94
170.40
158.69
158.25
118.61
63.07
51.71
26.74
23.93
20.71
TFA. H2N
O
OH
AcO
7
75 MHz
DMSO-d6
f1 (ppm)

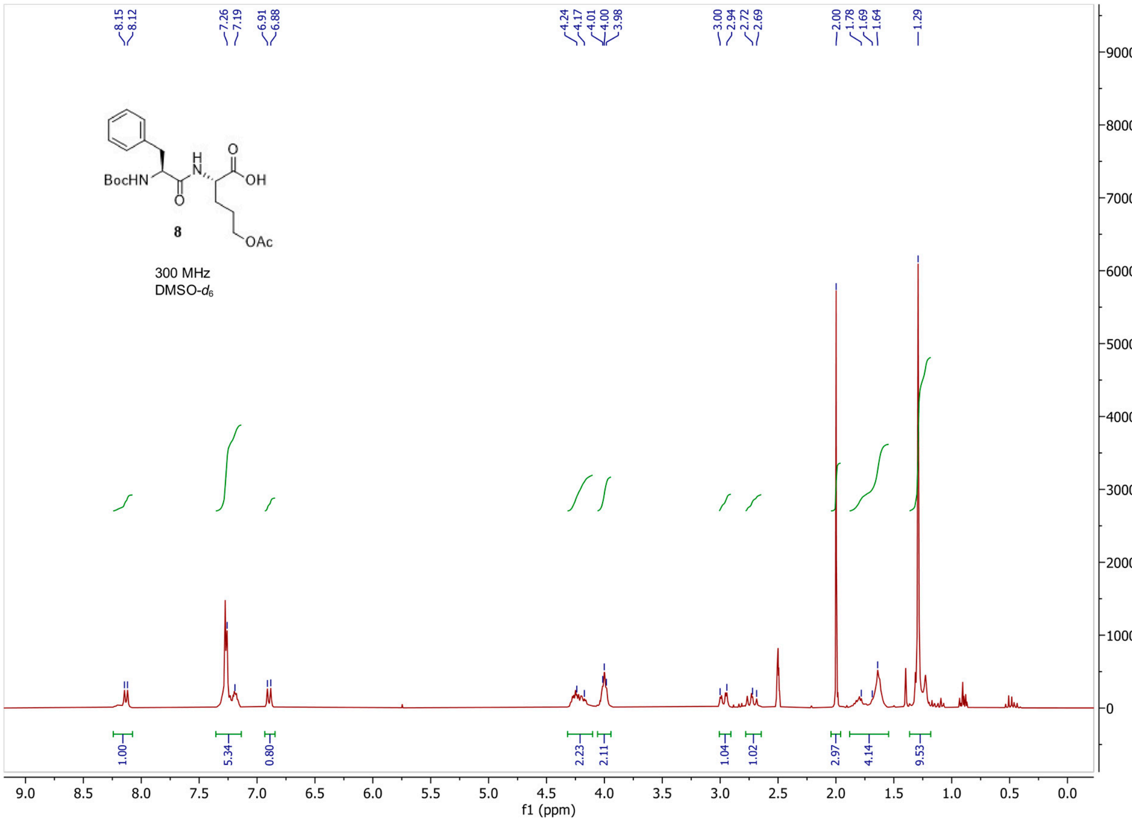
8.15
8.12
7.26
7.19
6.91
6.88
4.24
4.17
4.01
4.00
3.98
3.00
2.94
2.72
2.69
2.00
1.78
1.69
1.64
1.29
BocHN
H
N
O
OH
O
8
OAc
300 MHz
DMSO-d_6
1.00
5.34
0.80
2.23
2.11
1.04
1.02
2.97
4.14
9.53
f1 (ppm)

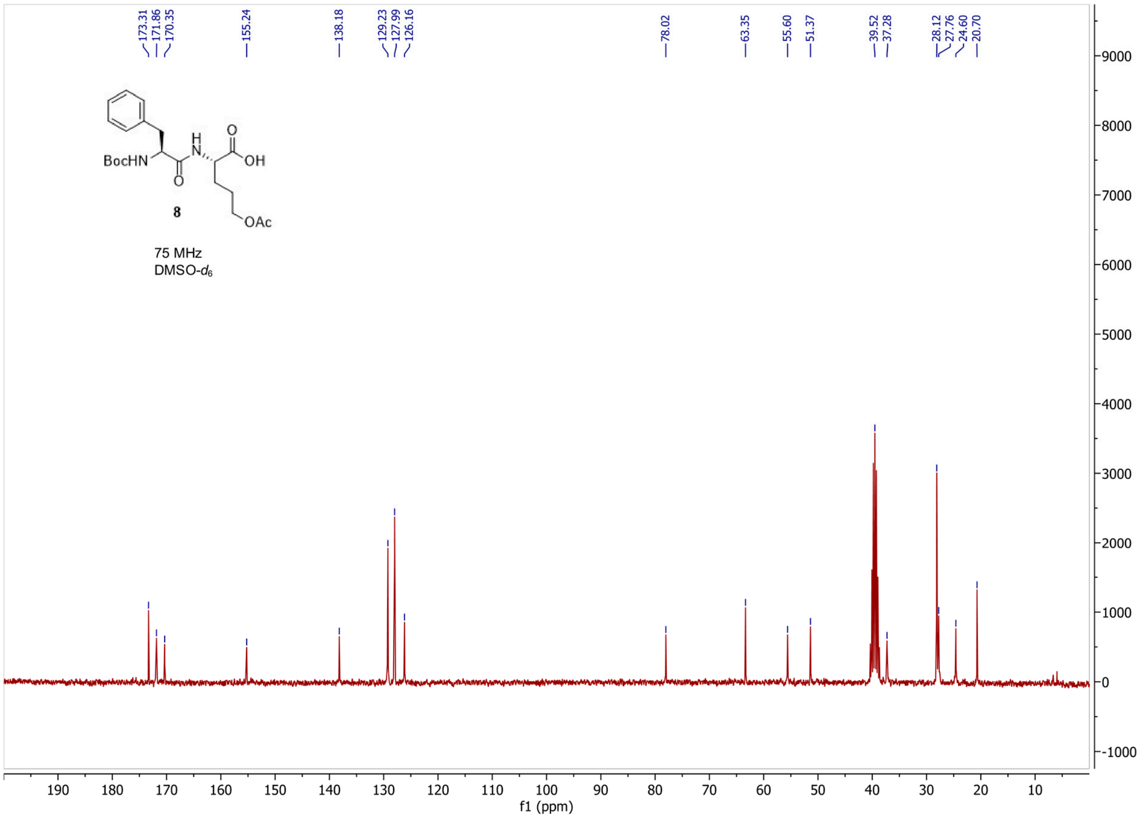
173.31
171.86
170.35
155.24
138.18
129.23
127.99
126.16
78.02
63.35
55.60
51.37
39.52
37.28
28.12
27.76
24.60
20.70
BocHN
H
N
O
OH
O
8
OAc
75 MHz
DMSO-d_6
f1 (ppm)

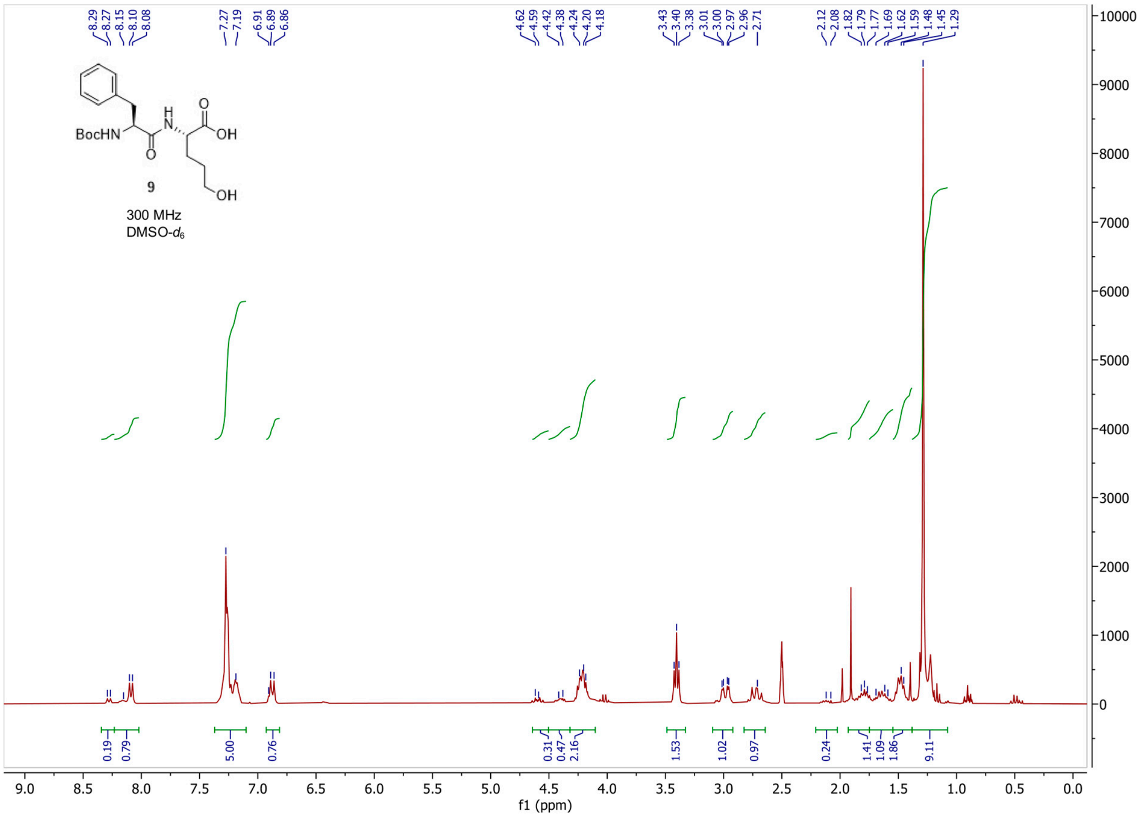
BocHN
OH
9
300 MHz
DMSO-d_6
f1 (ppm)

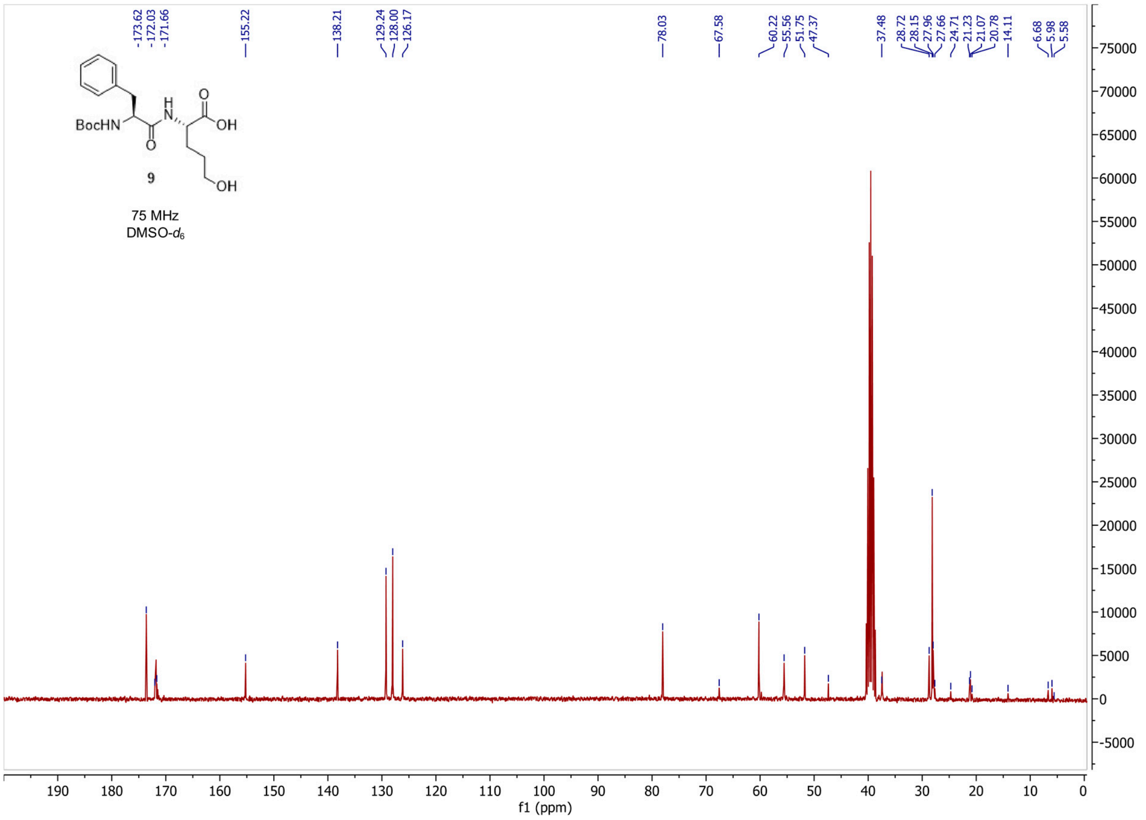
BocHN
OH
9
75 MHz
DMSO-d_6
f1 (ppm)

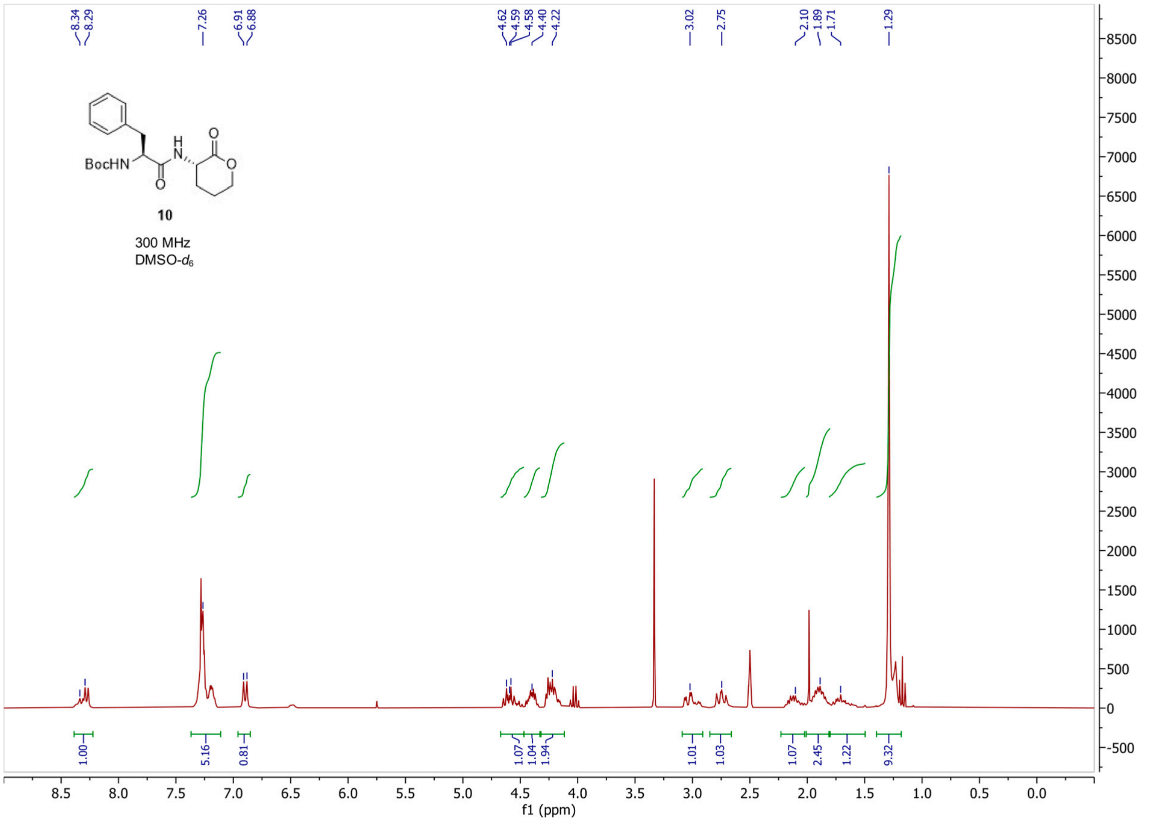
8.34
8.29
7.26
6.91
6.88
4.62
4.59
4.58
4.40
4.22
3.02
2.75
2.10
1.89
1.71
1.29
BocHN
10
300 MHz
DMSO-d_6
1.00
5.16
0.81
1.07
1.04
1.94
1.01
1.03
1.07
2.45
1.22
9.32
f1 (ppm)

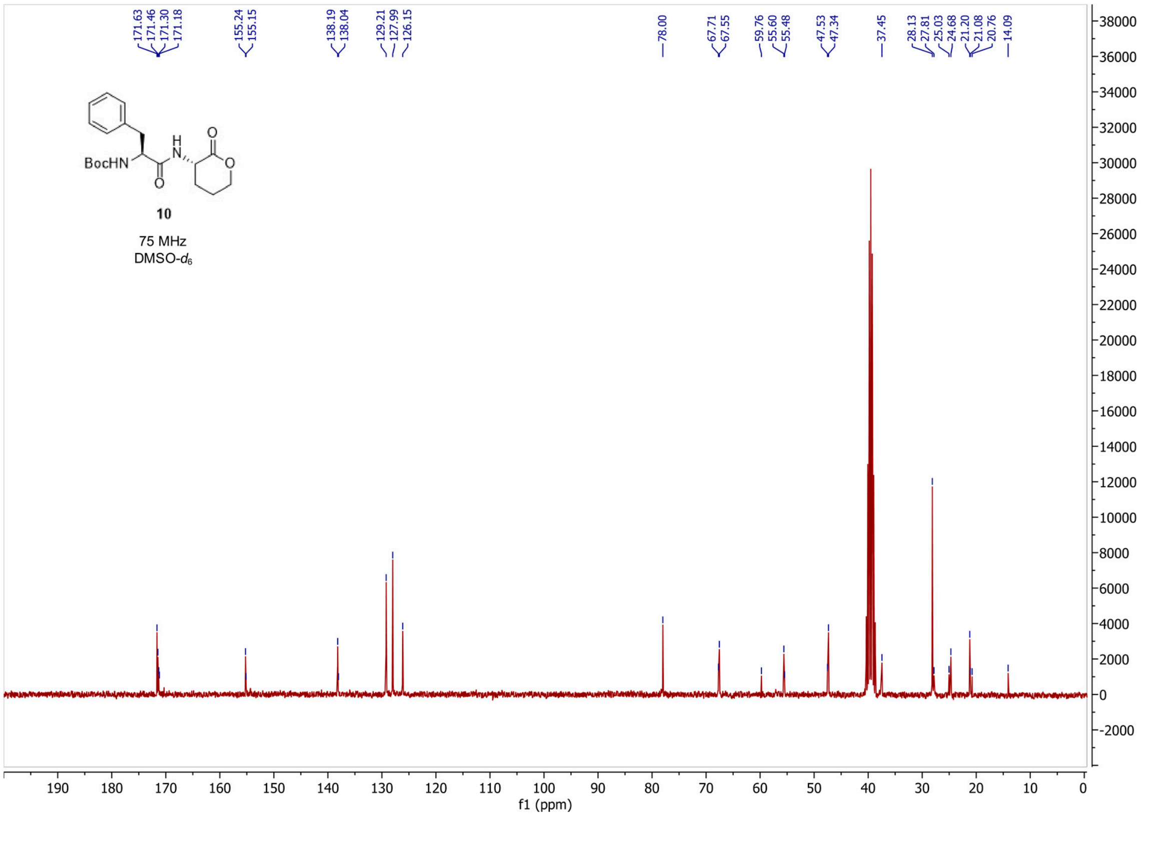
171.63
171.46
171.30
171.18
155.24
155.15
138.19
138.04
129.21
127.99
126.15
78.00
67.71
67.55
59.76
55.60
55.48
47.53
47.34
37.45
28.13
27.81
25.03
24.68
21.20
21.08
20.76
14.09
BocHN
10
75 MHz
DMSO-d_6
f1 (ppm)

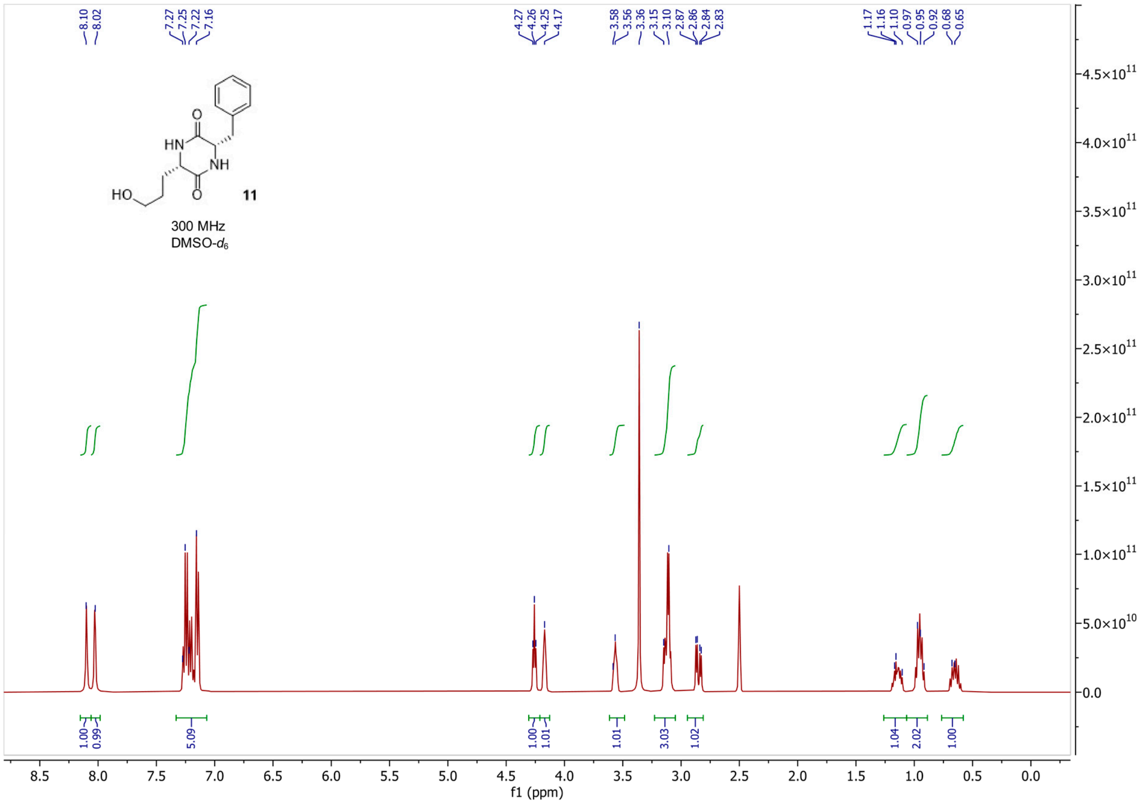

8.10
8.02
7.27
7.25
7.22
7.16
4.27
4.26
4.25
4.17
3.58
3.56
3.36
3.15
3.10
2.87
2.86
2.84
2.83
1.17
1.16
1.10
0.97
0.95
0.92
0.68
0.65
HN
O
NH
O
HO
11
300 MHz
DMSO-d_6
1.00
0.99
5.09
1.00
1.01
1.01
3.03
1.02
1.04
2.02
1.00
f1 (ppm)

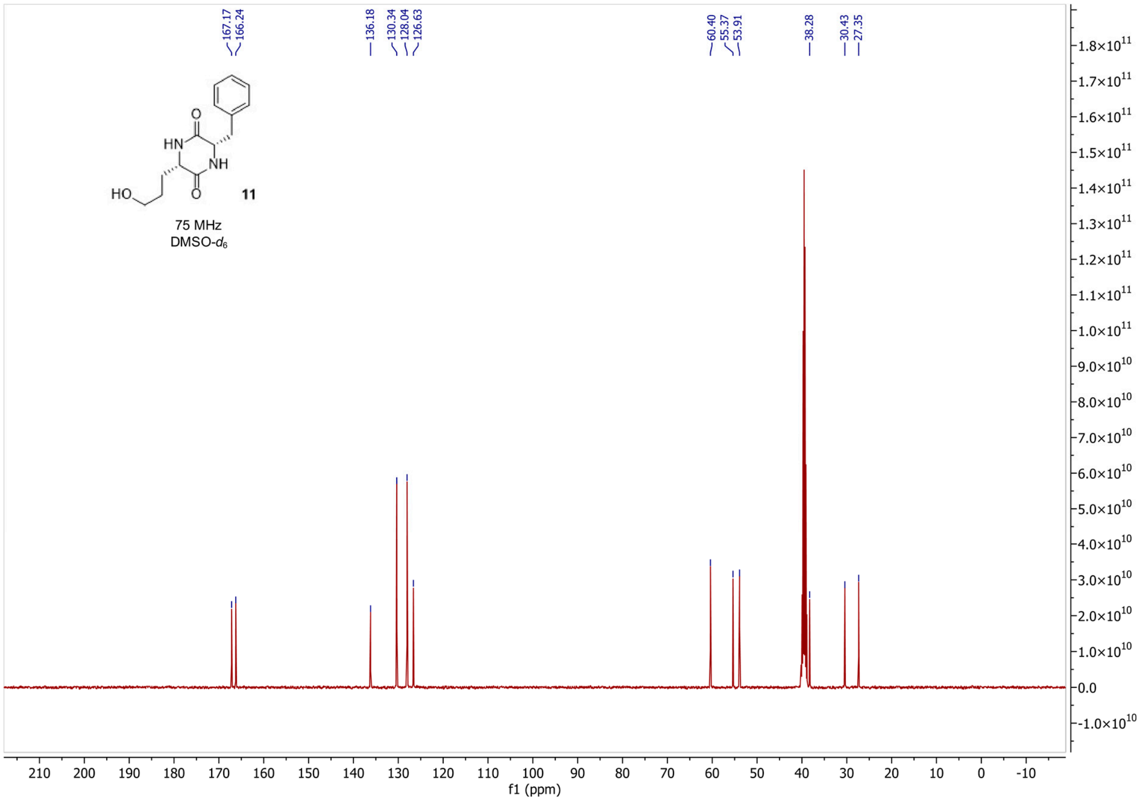

167.17
166.24
136.18
130.34
128.04
126.63
60.40
55.37
53.91
38.28
30.43
27.35
HN
O
NH
O
HO
11
75 MHz
DMSO-d_6
f1 (ppm)

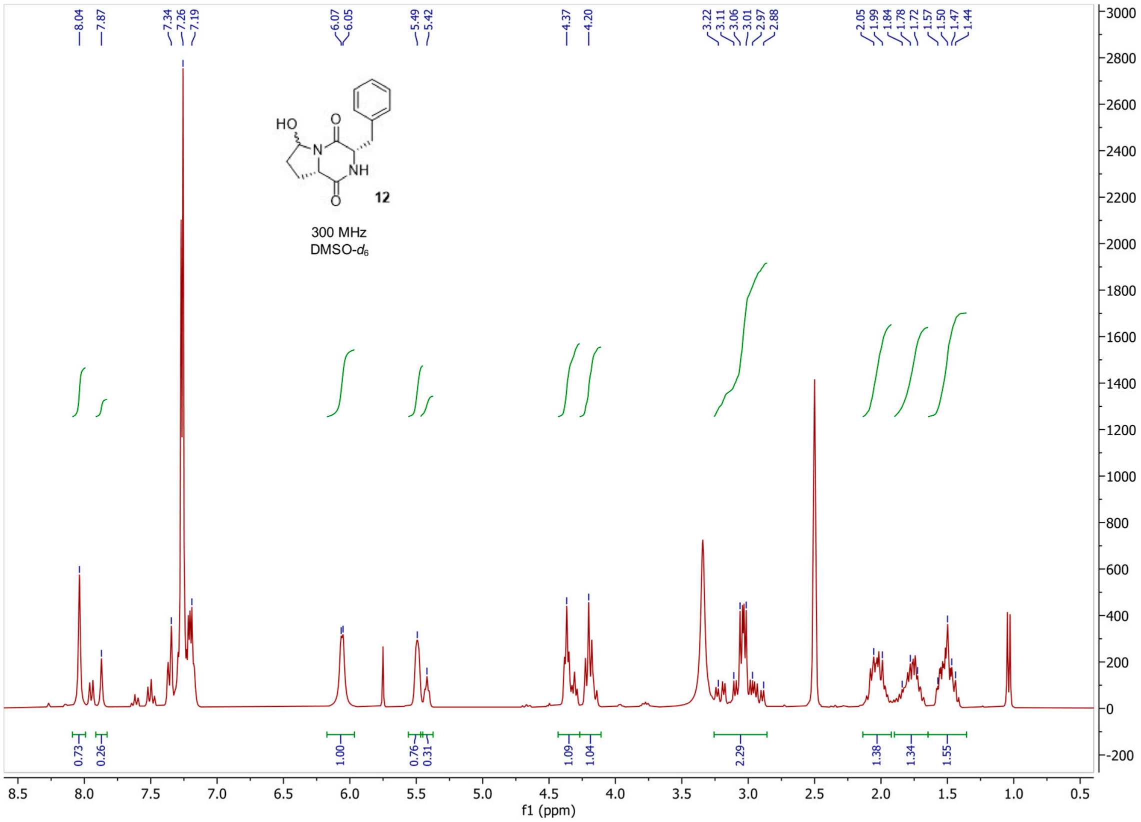
8.04
7.87
7.34
7.26
7.19
6.07
6.05
5.49
5.42
4.37
4.20
3.22
3.11
3.06
3.01
2.97
2.88
2.05
1.99
1.84
1.78
1.72
1.57
1.50
1.47
1.44
HO
O
N
NH
O
12
300 MHz
DMSO-d_6
0.73
0.26
1.00
0.76
0.31
1.09
1.04
2.29
1.38
1.34
1.55
f1 (ppm)

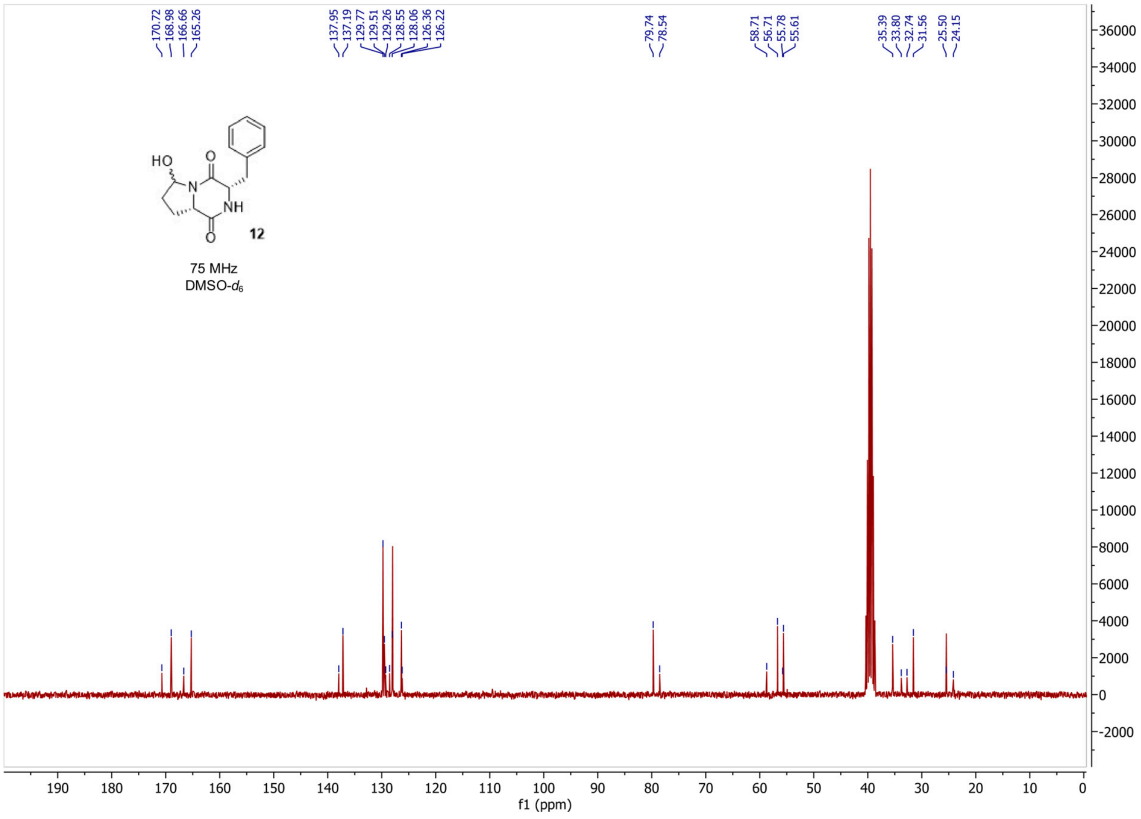
170.72
168.98
166.66
165.26
137.95
137.19
129.77
129.51
129.26
128.55
128.06
126.36
126.22
79.74
78.54
58.71
56.71
55.78
55.61
35.39
33.80
32.74
31.56
25.50
24.15
HO
O
N
NH
O
12
75 MHz
DMSO-d_6
f1 (ppm)

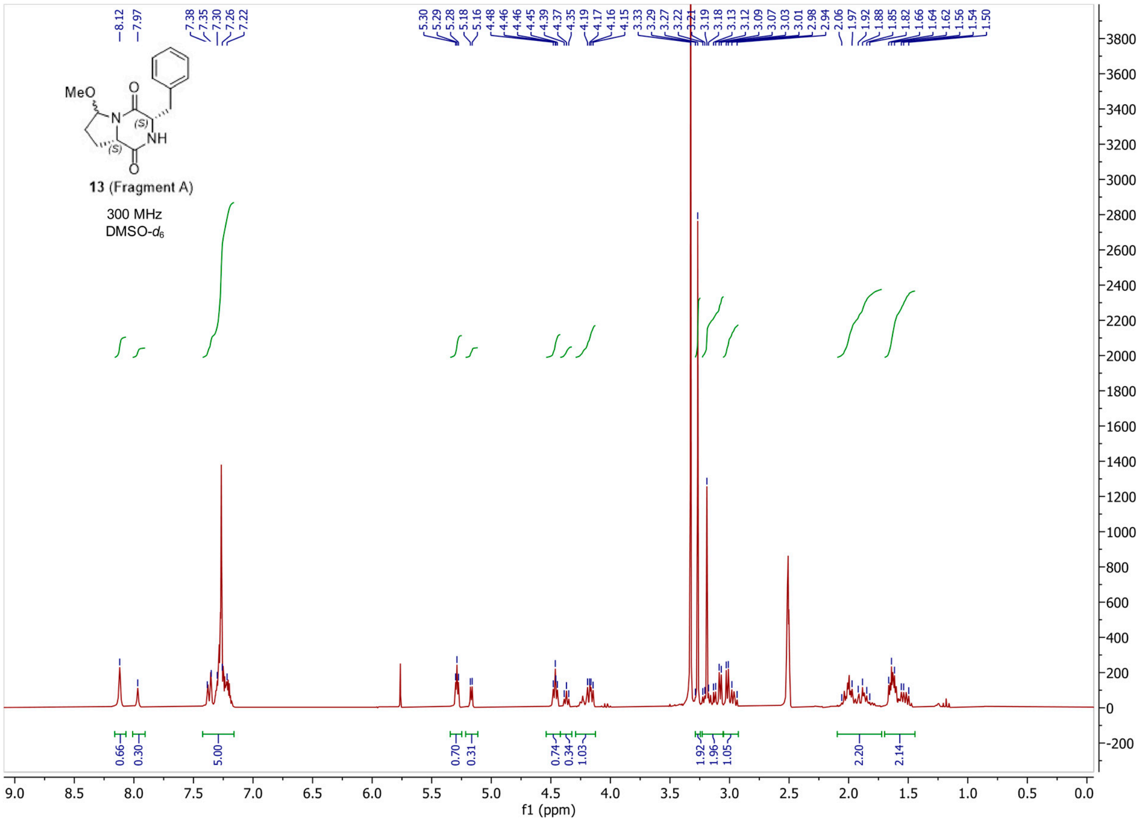

13 (Fragment A)
300 MHz
DMSO-d_6
f1 (ppm)

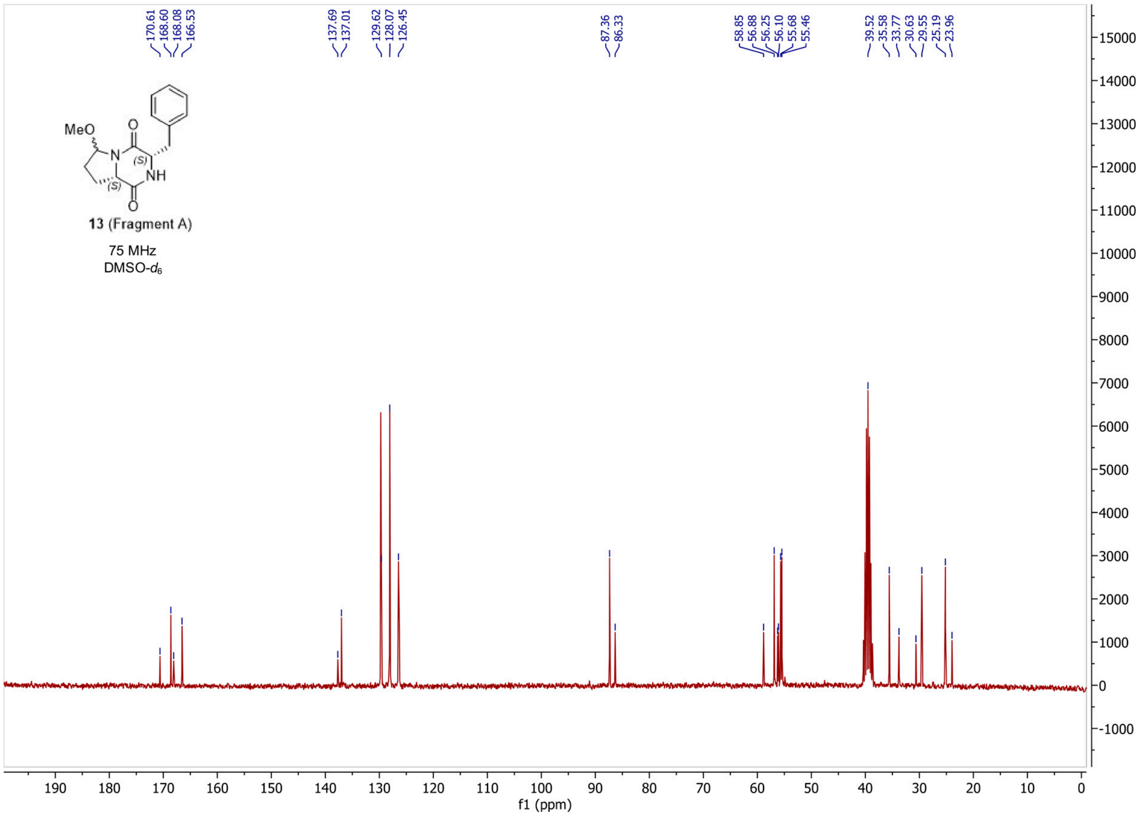

13 (Fragment A)
75 MHz
DMSO-d_6
f1 (ppm)

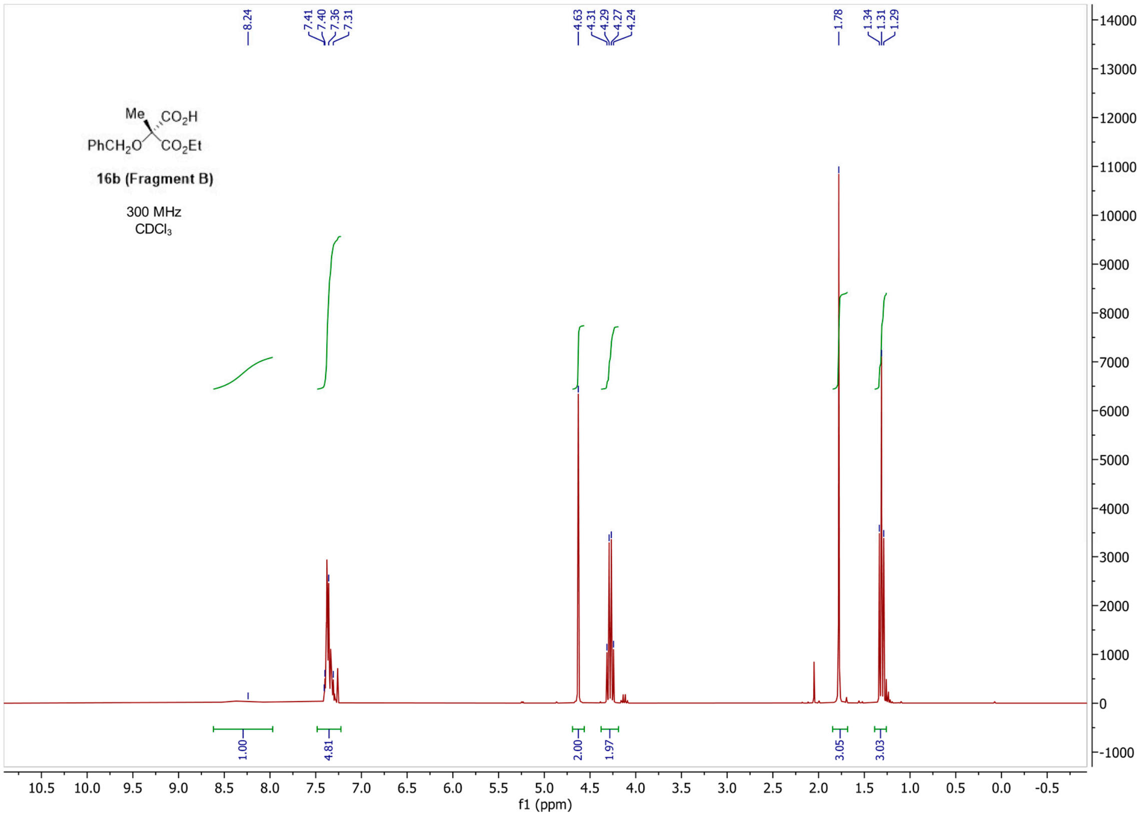
8.24
7.41
7.40
7.36
7.31
4.63
4.31
4.29
4.27
4.24
1.78
1.34
1.31
1.29
Me
CO2H
PhCH2O
CO2Et
16b (Fragment B)
300 MHz
CDCl3
1.00
4.81
2.00
1.97
3.05
3.03
f1 (ppm)

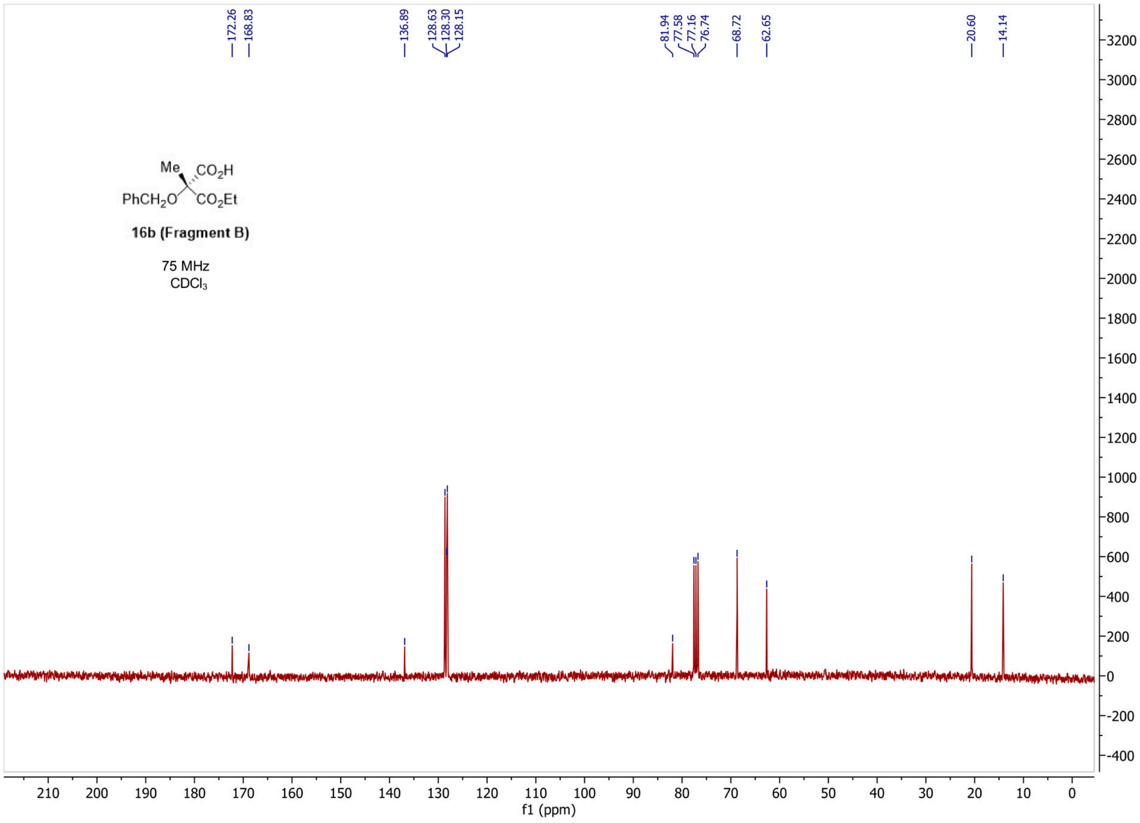
172.26
168.83
136.89
128.63
128.30
128.15
81.94
77.58
77.16
76.74
68.72
62.65
20.60
14.14
Me
CO2H
PhCH2O
CO2Et
16b (Fragment B)
75 MHz
CDCl3
f1 (ppm)

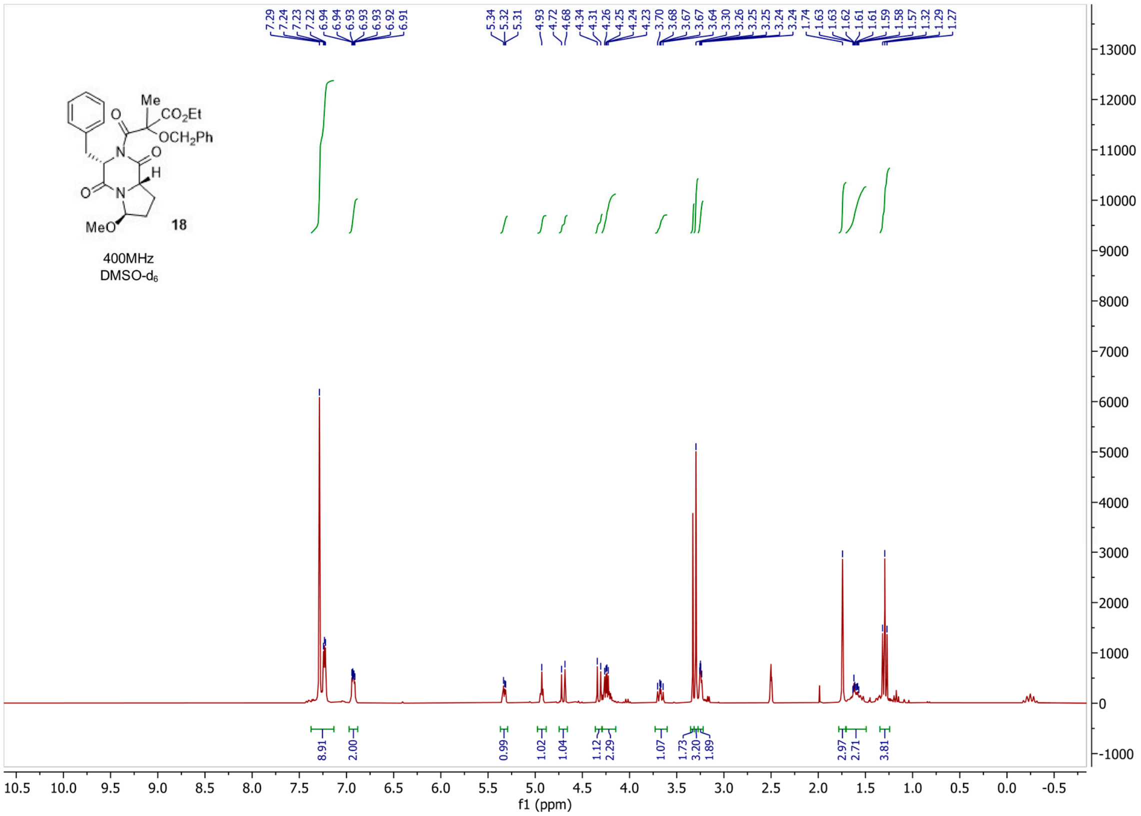
400MHz
DMSO-d6
18
f1 (ppm)

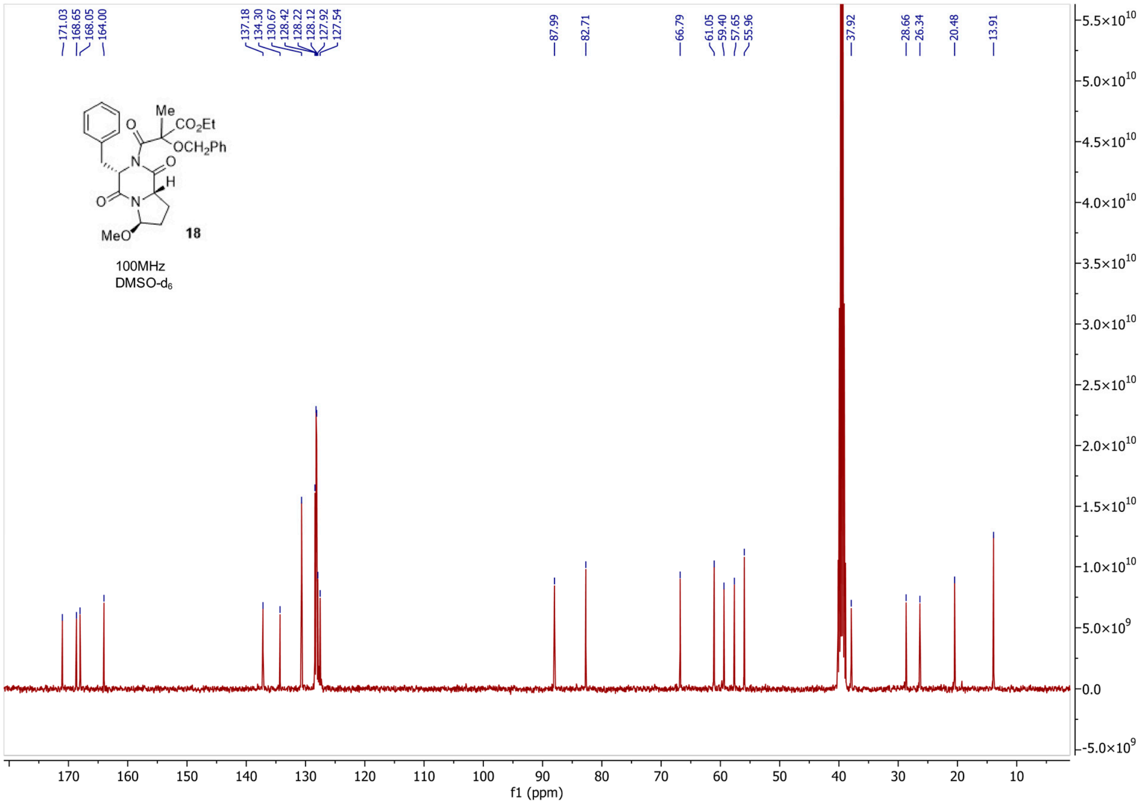
100MHz
DMSO-d6
18
f1 (ppm)

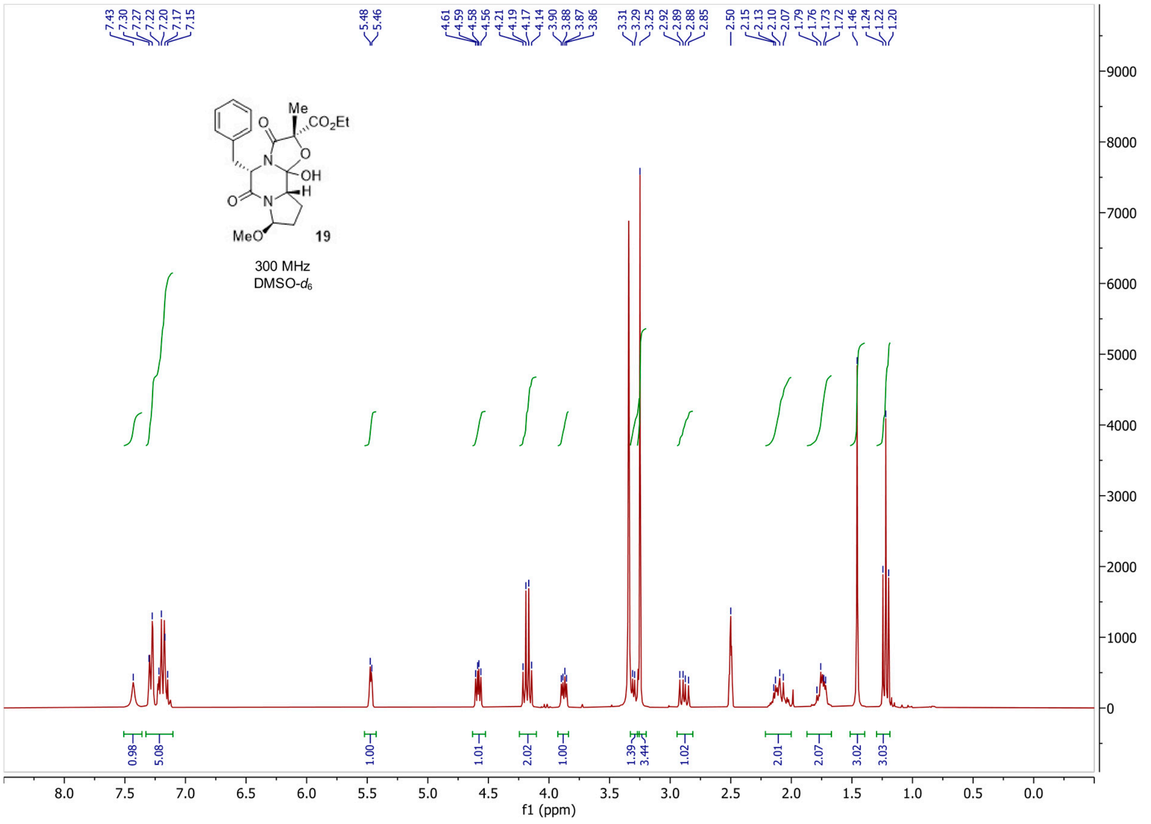
7.43
7.30
7.27
7.22
7.20
7.17
7.15
5.48
5.46
4.61
4.59
4.58
4.56
4.21
4.19
4.17
4.14
3.90
3.88
3.87
3.86
3.31
3.29
3.25
2.92
2.89
2.88
2.85
2.50
2.15
2.13
2.10
2.07
1.79
1.76
1.73
1.72
1.46
1.24
1.22
1.20
Me
CO2Et
O
N
O
OH
H
O
N
MeO
19
300 MHz
DMSO-d6
0.98
5.08
1.00
1.01
2.02
1.00
1.39
3.44
1.02
2.01
2.07
3.02
3.03
f1 (ppm)

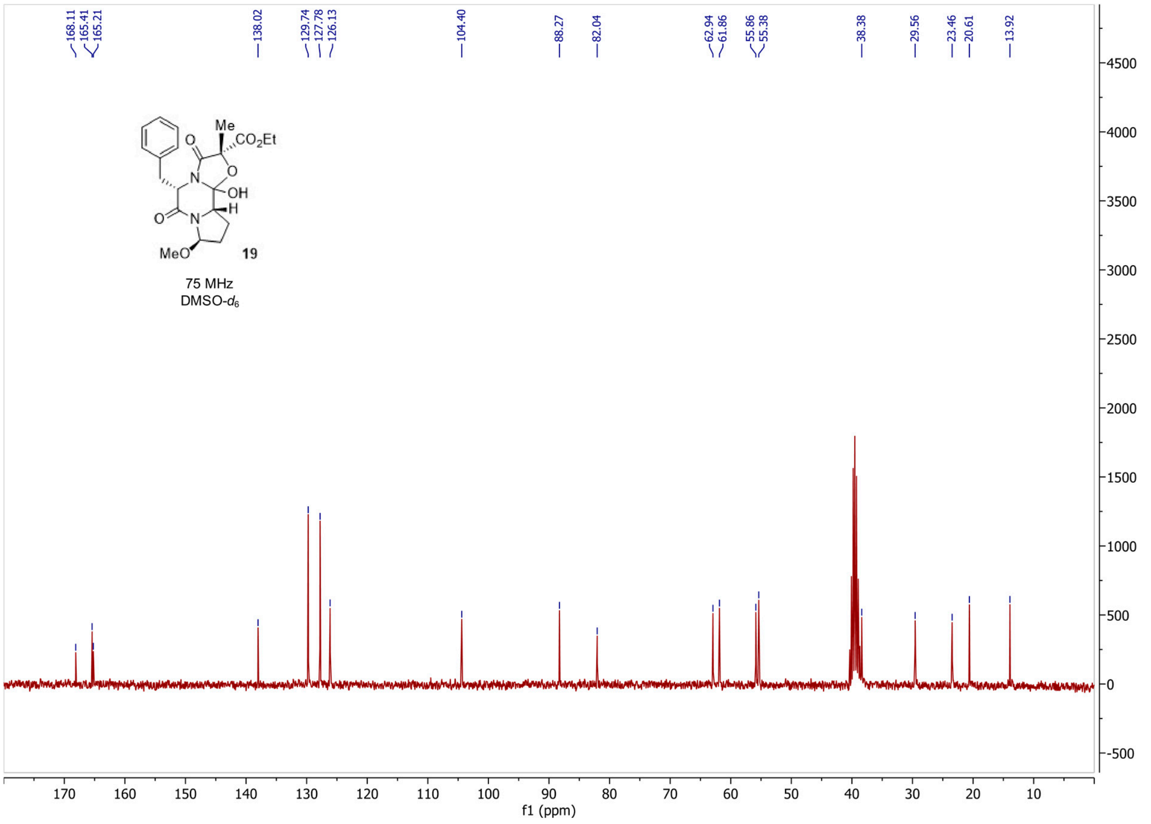
168.11
165.41
165.21
138.02
129.74
127.78
126.13
104.40
88.27
82.04
62.94
61.86
55.86
55.38
38.38
29.56
23.46
20.61
13.92
Me
CO2Et
O
N
O
OH
H
O
N
MeO
19
75 MHz
DMSO-d6
f1 (ppm)

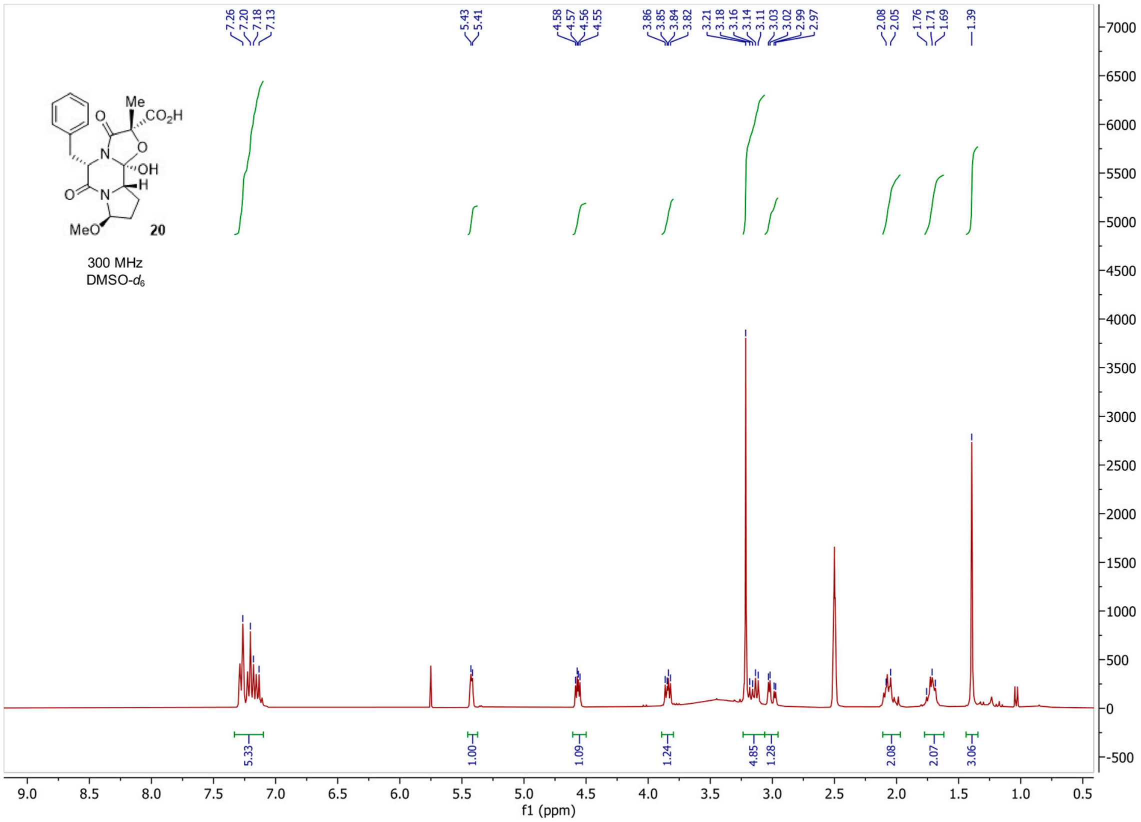
7.26
7.20
7.18
7.13
5.43
5.41
4.58
4.57
4.56
4.55
3.86
3.85
3.84
3.82
3.21
3.18
3.16
3.14
3.11
3.03
3.02
2.99
2.97
2.08
2.05
1.76
1.71
1.69
1.39
Me
CO_2H
O
N
O
OH
H
O
N
MeO
20
300 MHz
DMSO-d_6
5.33
1.00
1.09
1.24
4.85
1.28
2.08
2.07
3.06
f1 (ppm)

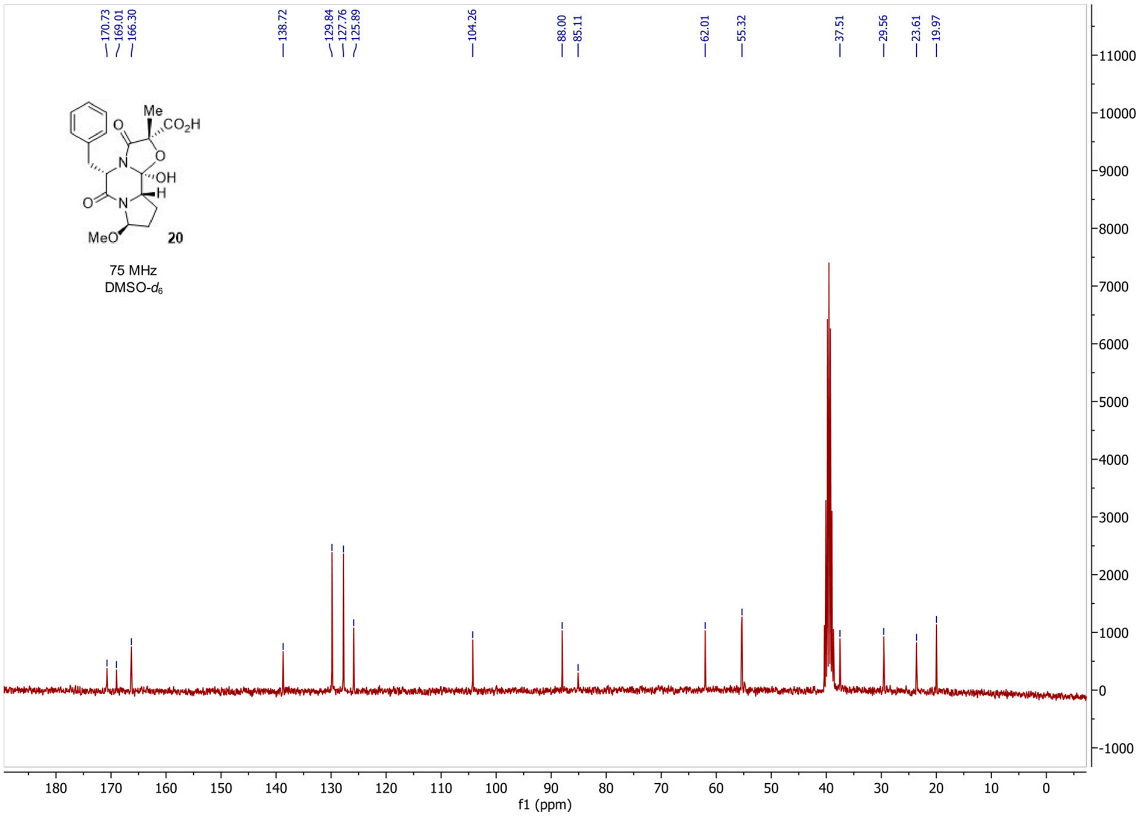
170.73
169.01
166.30
138.72
129.84
127.76
125.89
104.26
88.00
85.11
62.01
55.32
37.51
29.56
23.61
19.97
Me
CO_2H
O
N
O
OH
H
O
N
MeO
20
75 MHz
DMSO-d_6
f1 (ppm)

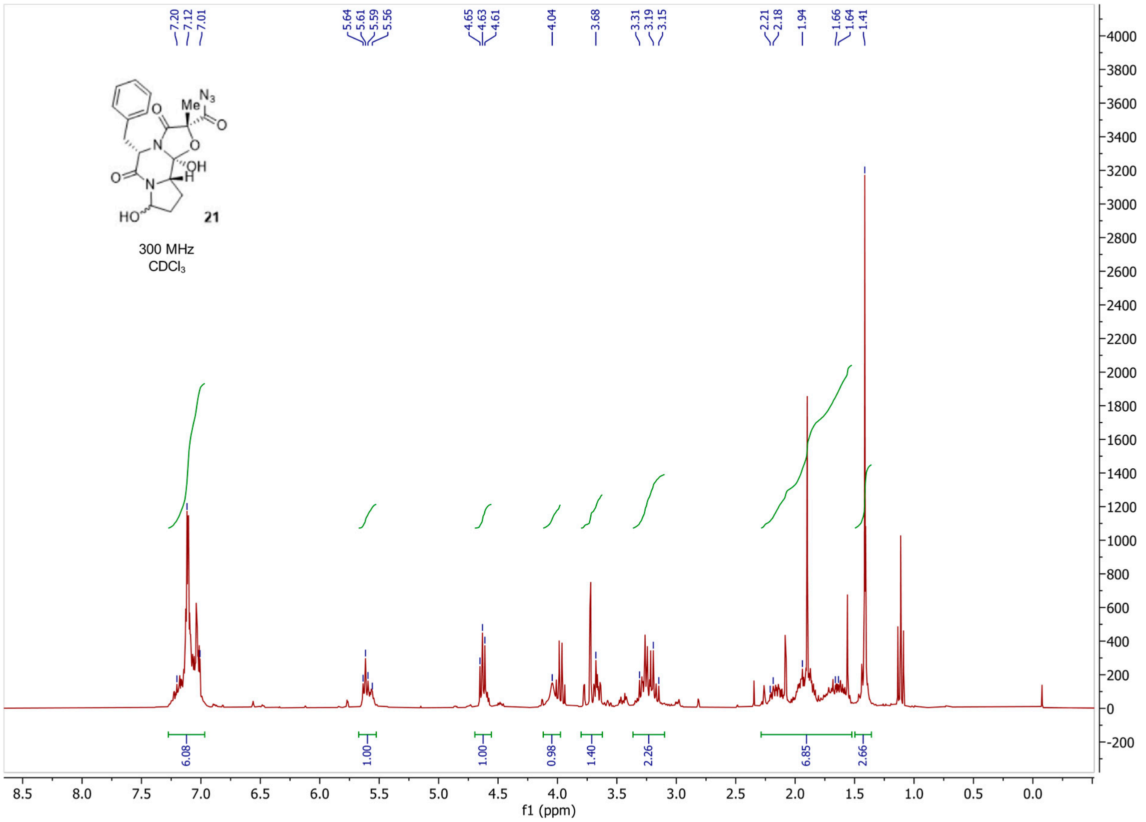
7.20
7.12
7.01
5.64
5.61
5.59
5.56
4.65
4.63
4.61
4.04
3.68
3.31
3.19
3.15
2.21
2.18
1.94
1.66
1.64
1.41
21
300 MHz
CDCl3
6.08
1.00
1.00
0.98
1.40
2.26
6.85
2.66
f1 (ppm)

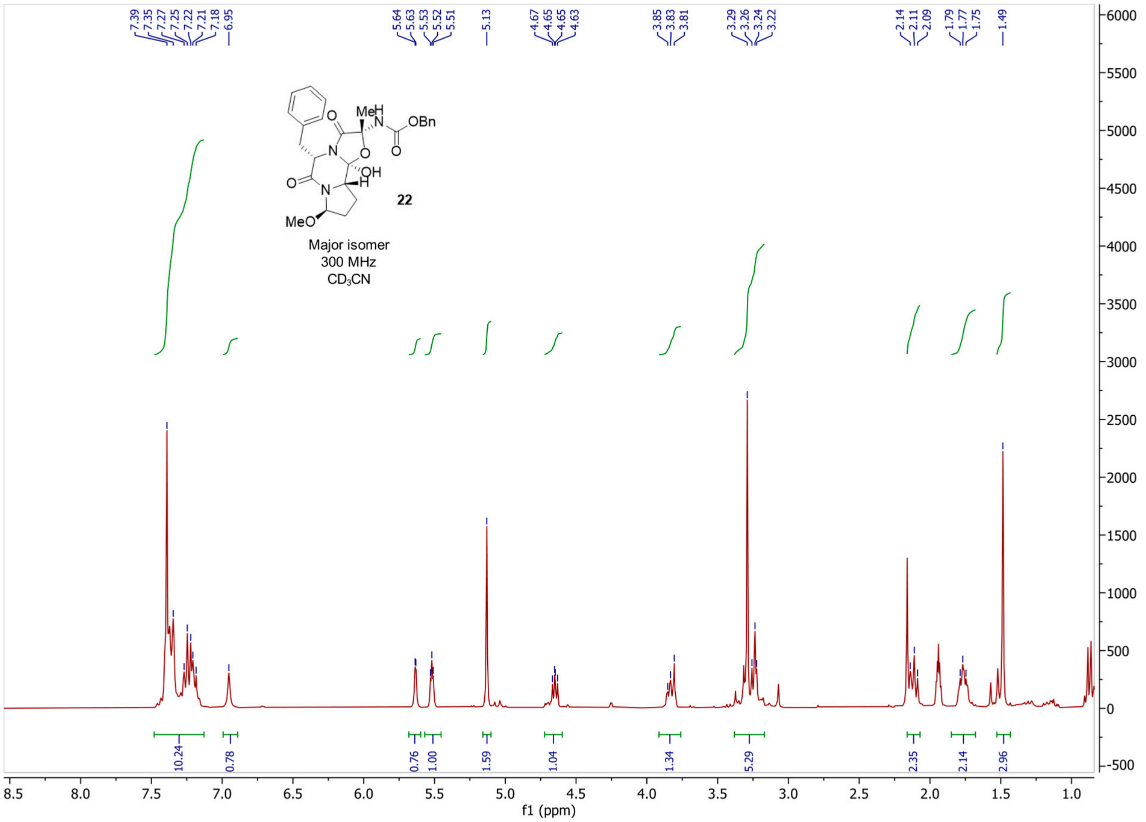
7.39
7.35
7.27
7.25
7.22
7.21
7.18
6.95
5.64
5.63
5.53
5.52
5.51
5.13
4.67
4.65
4.65
4.63
3.85
3.83
3.81
3.29
3.26
3.24
3.22
2.14
2.11
2.09
1.79
1.77
1.75
1.49
MeH
N
OBn
O
O
N
O
OH
H
O
N
MeO
22
Major isomer
300 MHz
CD_3CN
10.24
0.78
0.76
1.00
1.59
1.04
1.34
5.29
2.35
2.14
2.96
f1 (ppm)

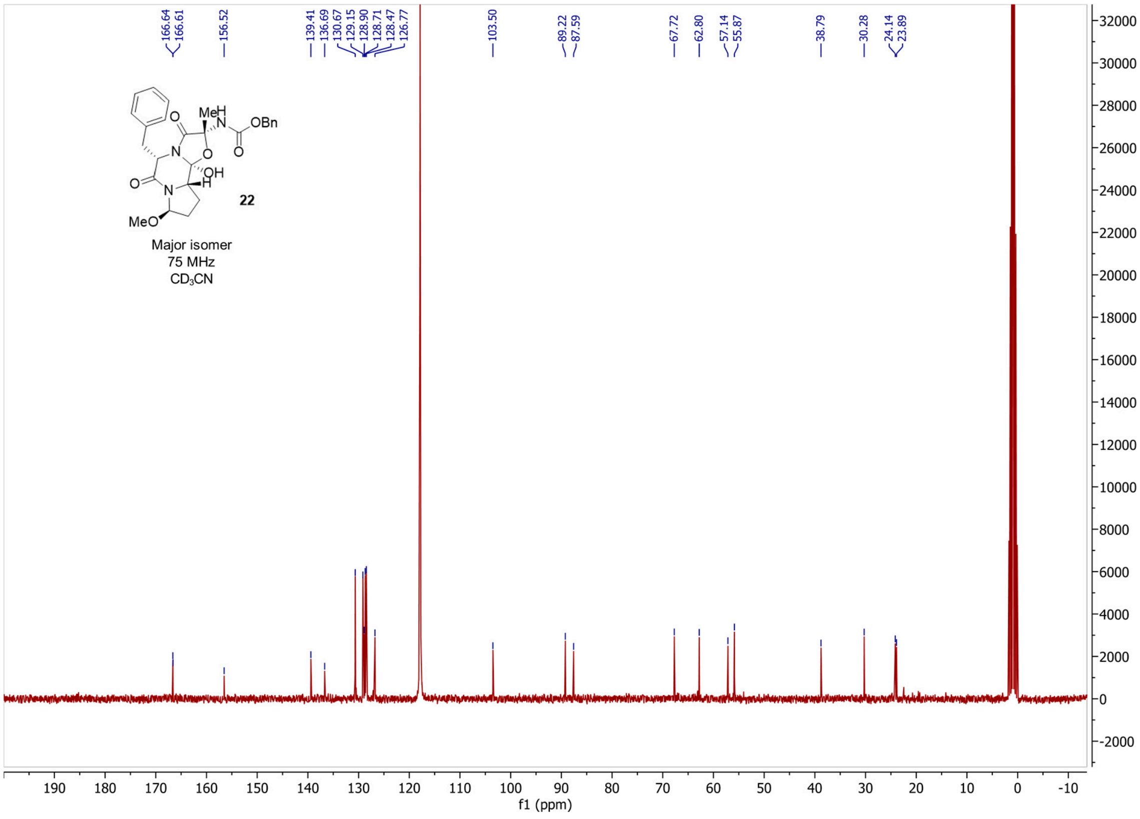
166.64
166.61
156.52
139.41
136.69
130.67
129.15
128.90
128.71
128.47
126.77
103.50
89.22
87.59
67.72
62.80
57.14
55.87
38.79
30.28
24.14
23.89
MeH
N
OBn
O
O
N
O
OH
H
O
N
MeO
22
Major isomer
75 MHz
CD_3CN
f1 (ppm)

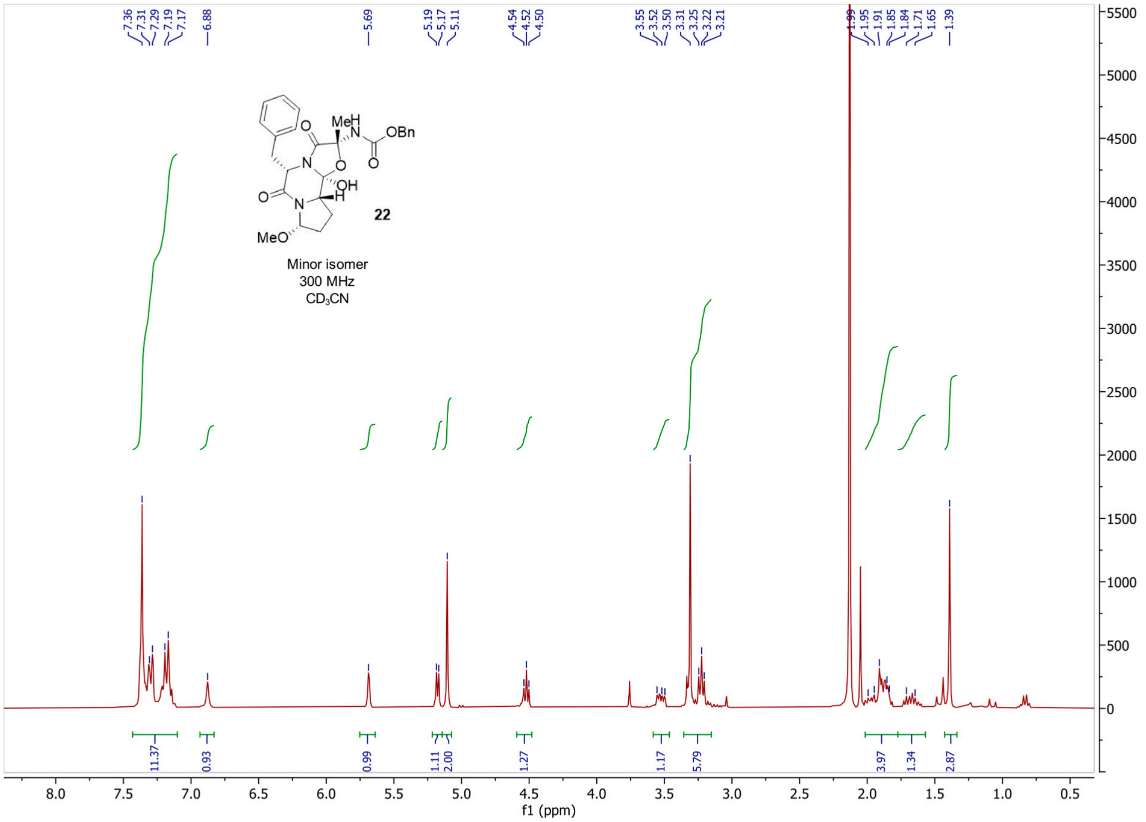
7.36 7.31 7.29 7.19 7.17 6.88 5.69 5.19 5.17 5.11 4.54 4.52 4.50 3.55 3.52 3.50 3.31 3.25 3.22 3.21 1.99 1.95 1.91 1.85 1.84 1.71 1.65 1.39
MeH
N
OBn
O
N
O
O
OH
H
N
22
MeO
Minor isomer
300 MHz
CD_3CN
11.37 0.93 0.99 1.11 2.00 1.27 1.17 5.79 3.97 1.34 2.87
f1 (ppm)

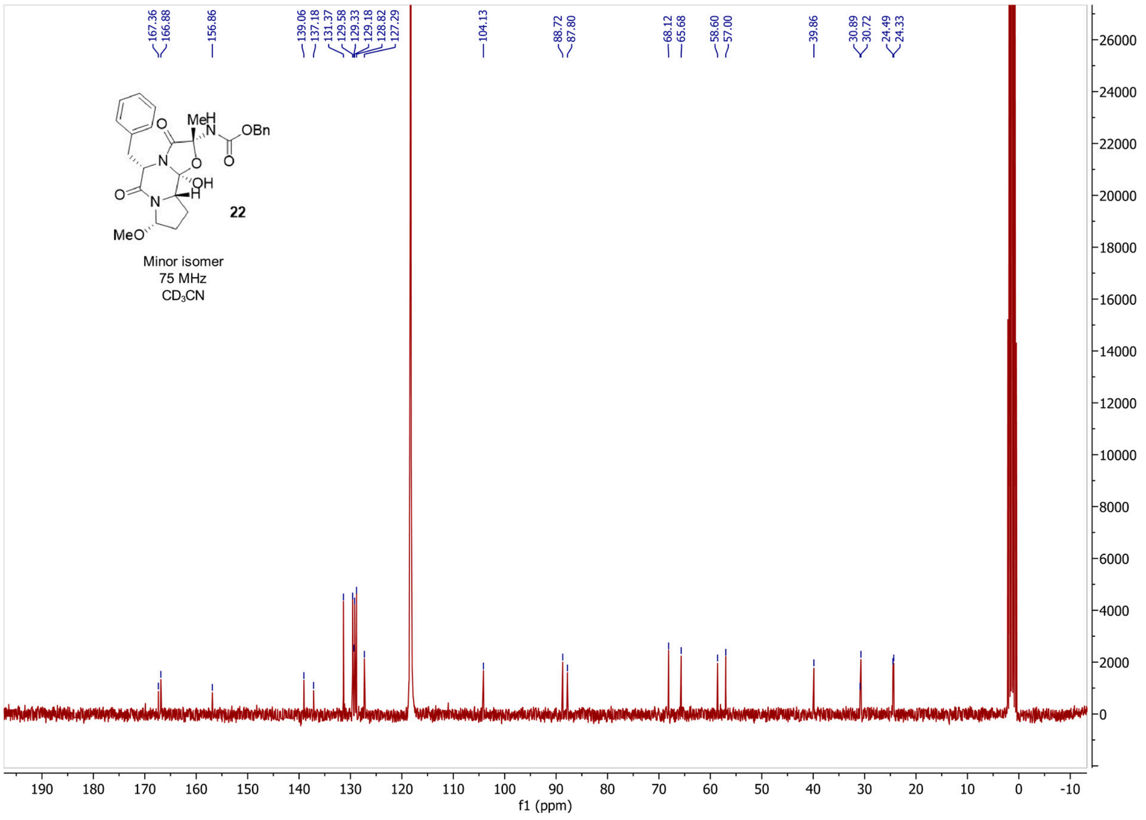
167.36 166.88 156.86 139.06 137.18 131.37 129.58 129.33 129.18 128.82 127.29 104.13 88.72 87.80 68.12 65.68 58.60 57.00 39.86 30.89 30.72 24.49 24.33
MeH
N
OBn
O
N
O
O
OH
H
N
22
MeO
Minor isomer
75 MHz
CD_3CN
f1 (ppm)

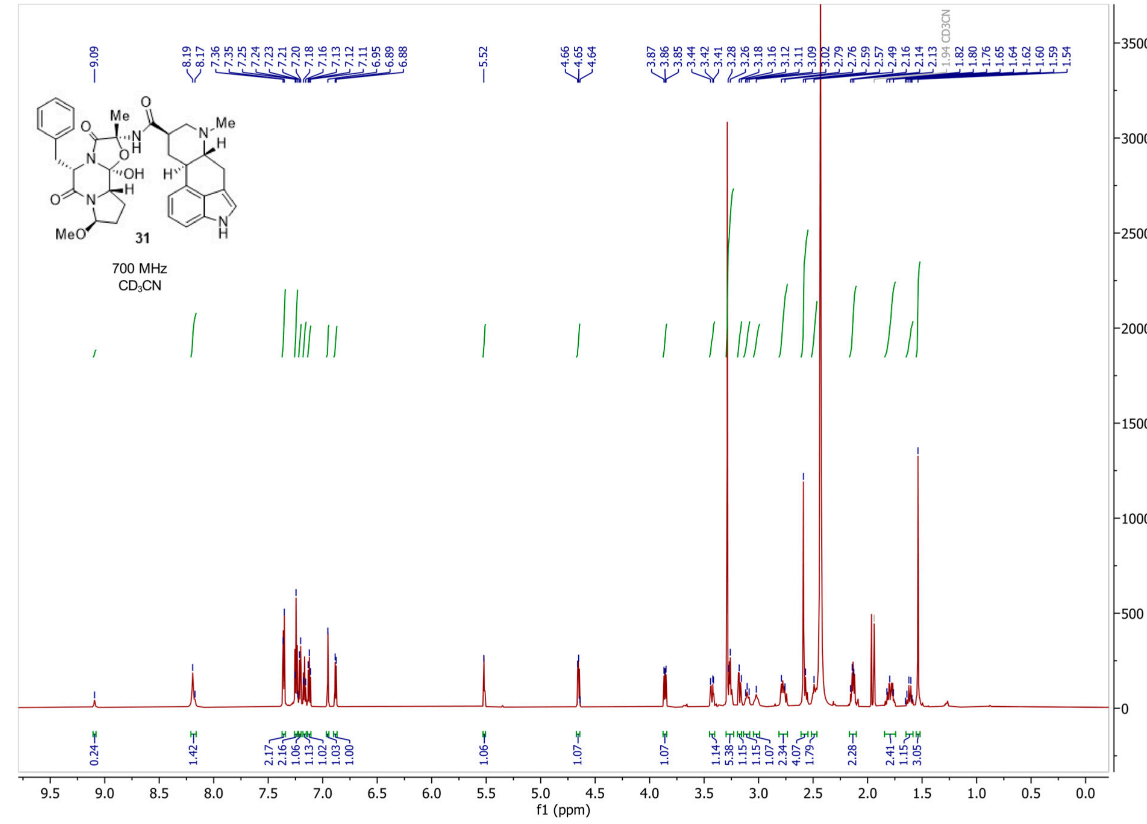
31
700 MHz
CD_3CN
f1 (ppm)

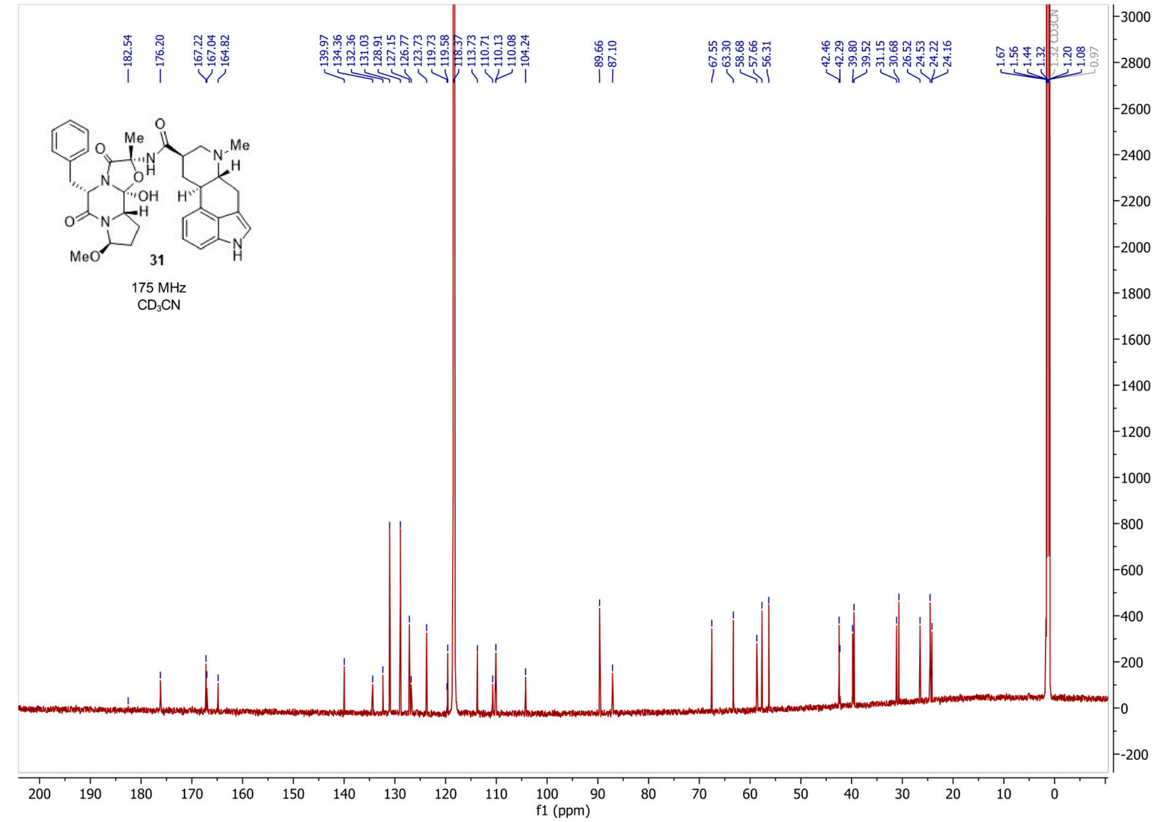
31
175 MHz
CD_3CN
f1 (ppm)

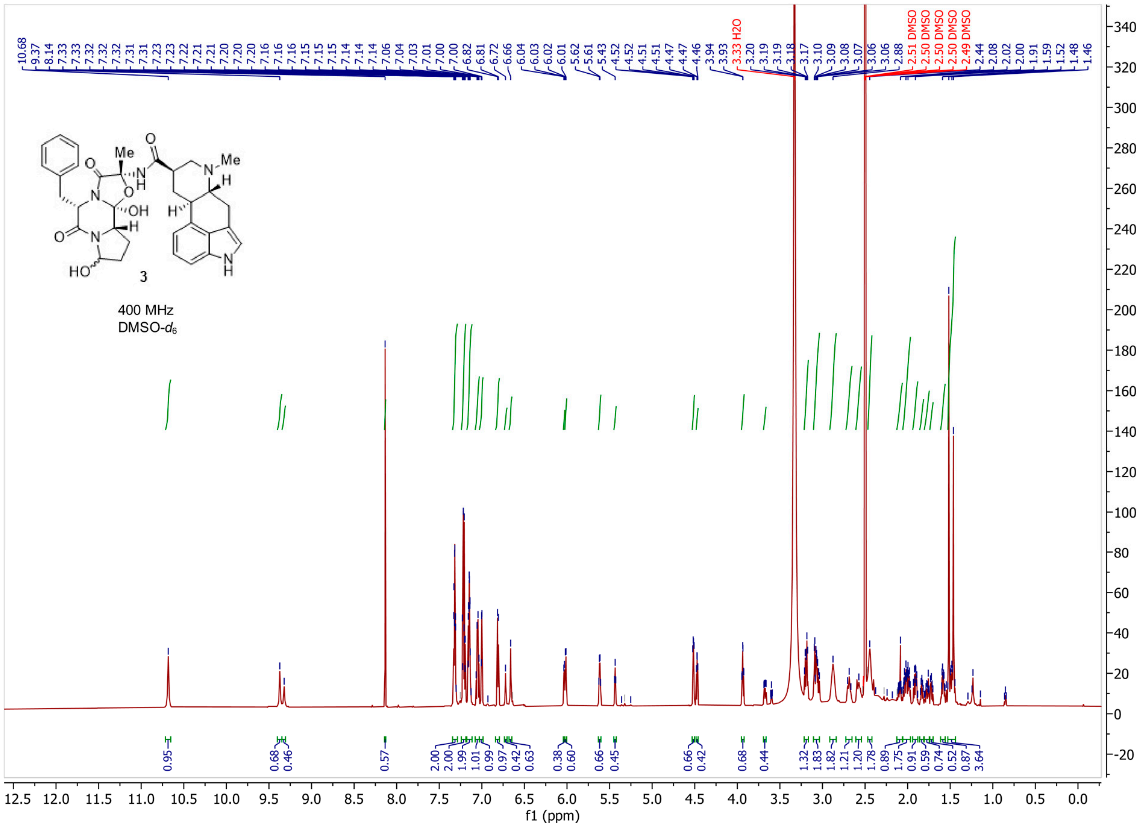
3
400 MHz
DMSO-d_6
f1 (ppm)

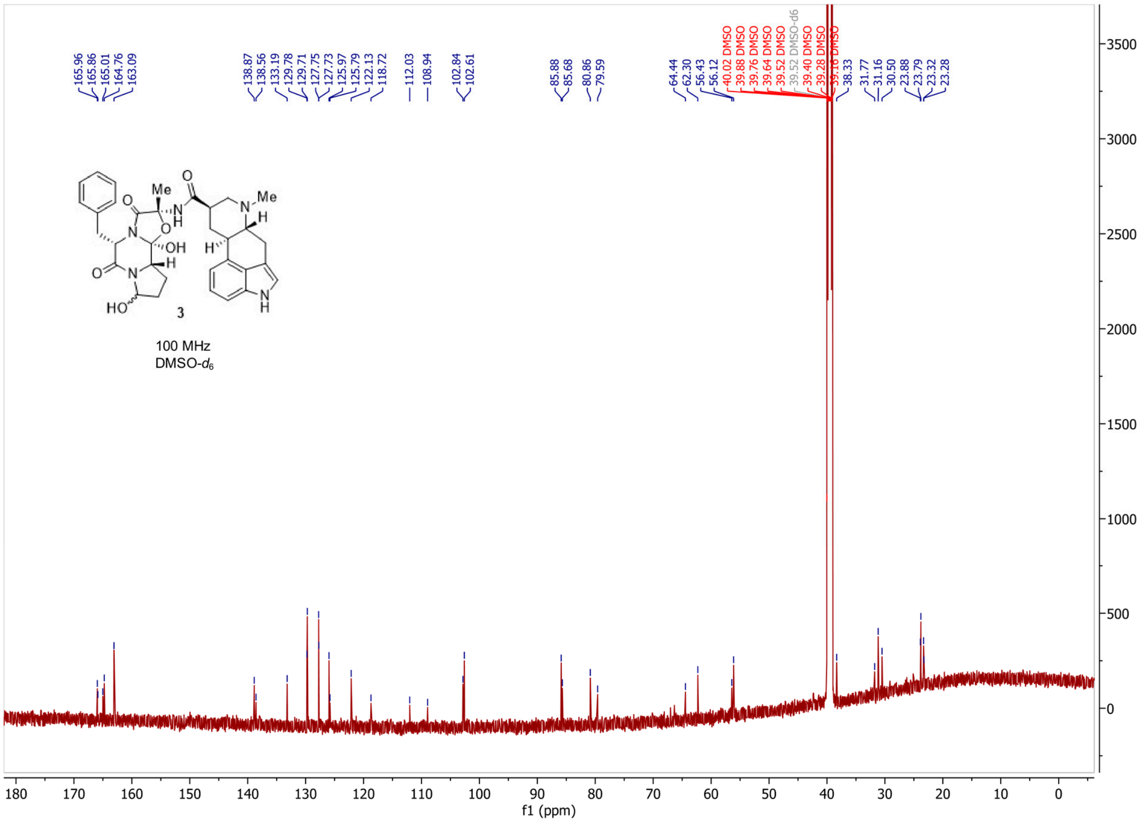
3
100 MHz
DMSO-d_6
f1 (ppm)

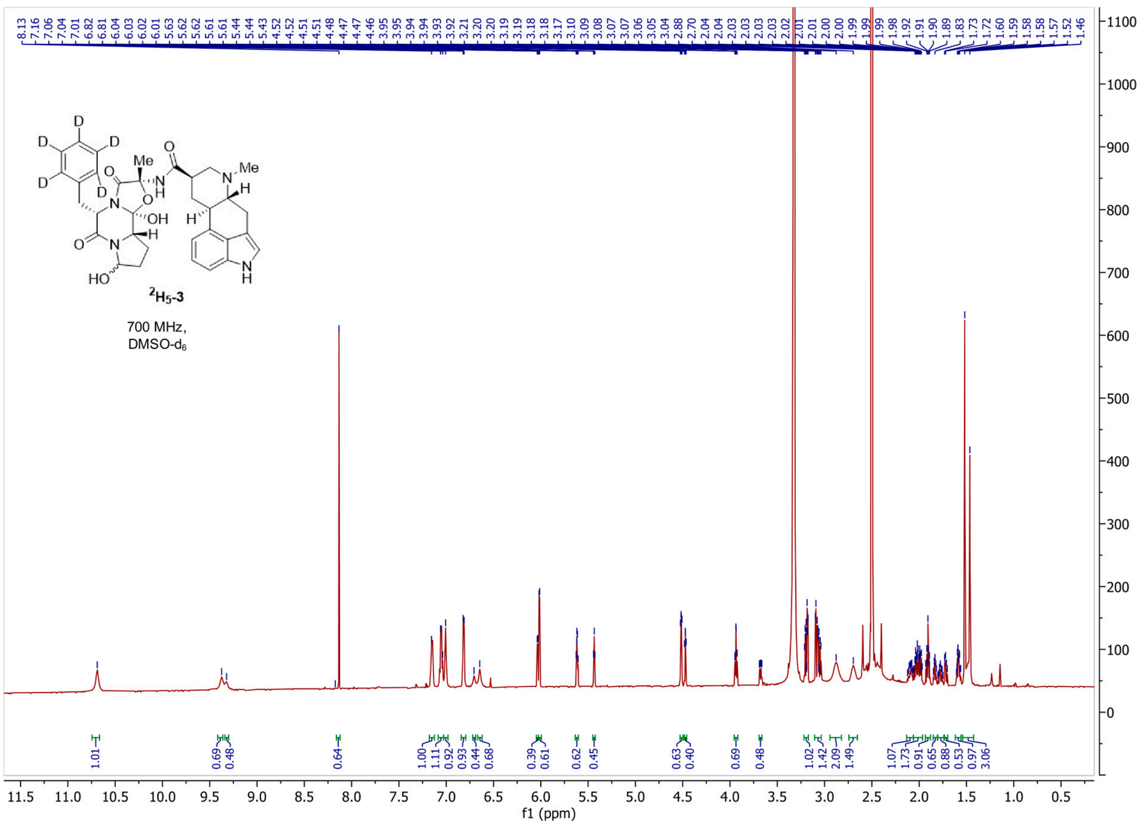

$^2H_5$-3
700 MHz,
DMSO-d6
f1 (ppm)

3. Crystallography Data

Single crystal X-ray diffraction data for compound **19** were collected on a Rigaku XtaLAB Synergy-S diffractometer fitted with a PhotonJet micro-focus sealed X-ray tube (Cu Kα radiation, λ = 1.54184 Å) and a HyPix-Arc 100° detector. Crystals were transferred to a glass slide and extracted under oil (Fomblin YR-1800) and mounted onto a 35 μm MiTeGen kapton loop before being flash-cooled to 150 K under N_2 using an Oxford Cryosystems cryostream prior to collection. Unit cell measurement, data collection and data reduction were performed using the software CrysAlisPRO. A numerical absorption correction was applied therein using Gaussian integration over a multi-faceted crystal model. The structure of **19** was solved using SHELXT and refined using SHELXL through the Olex2 interface. All non-hydrogen atoms were refined using anisotropic displacement parameters. Hydrogen atoms on heteroatoms were located using the electron density difference map and refined with U(iso) values fixed at 1.2 times the magnitude of the U(eq) of the parent heteroatom. All other hydrogen atoms were generated in calculated positions and refined using a riding model. Crystallographic data and specific refinement details can be found within the Crystallographic Information File, which is deposited in the Cambridge Crystallographic Data Centre (deposition number: CCDC 2469731).

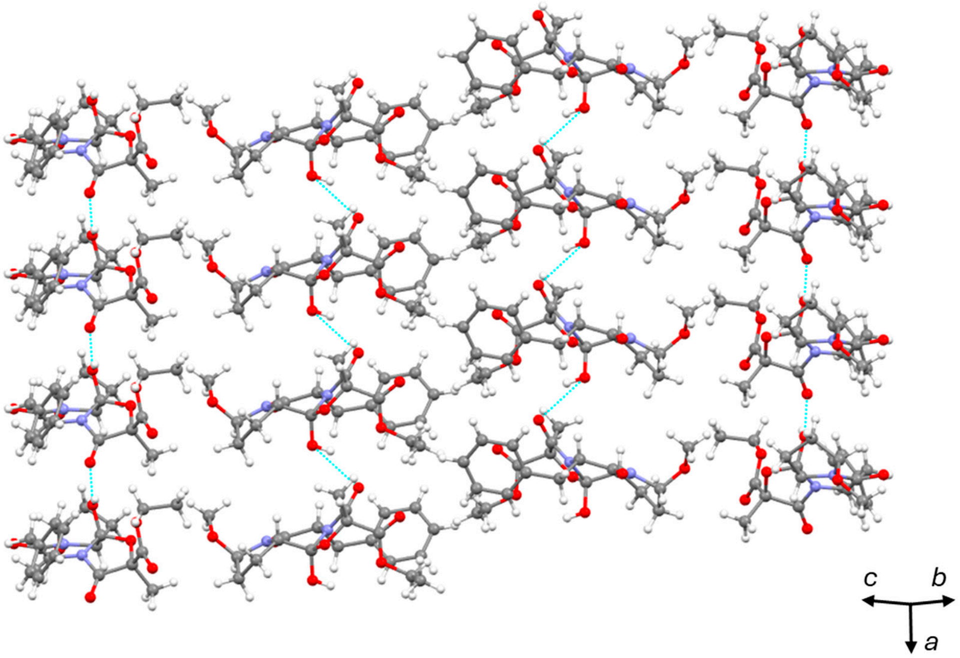

Figure S1 – A ball and stick depiction of the packing observed in **19**, as viewed along the [011] direction. The C(6) hydrogen bonding interactions are depicted by cyan dotted lines. Key: N – blue, O – red, C – grey, H – white.

Table S1 – Crystallographic tables for **19**.

Identification code	CCDC 2469731
Empirical formula	$C_{21}H_{26}N_2O_7$
Formula weight	418.44
Temperature/K	150.0(1)
Crystal system	orthorhombic
Space group	$P2_12_12_1$
a/Å	5.91380(10)
b/Å	17.4237(2)
c/Å	19.6948(2)
α/°	90
β/°	90
γ/°	90
Volume/Å^3	2029.36(5)
Z	4
ρ_{calc}g/cm^3	1.370
μ/mm^{-1}	0.863
F(000)	888.0
Crystal size/mm^3	0.601 × 0.115 × 0.045
Radiation	Cu Kα (λ = 1.54184)
2Θ range for data collection/°	6.774 to 154.576
Index ranges	$-5 \leq h \leq 7$, $-22 \leq k \leq 21$, $-23 \leq l \leq 23$
Reflections collected	19101
Independent reflections	4006 [R_{int} = 0.0223, R_{sigma} = 0.0153]
Data/restraints/parameters	4006/0/277
Goodness-of-fit on F^2	1.054
Final R indexes [I>=2σ (I)]	R_1 = 0.0248, wR_2 = 0.0638
Final R indexes [all data]	R_1 = 0.0254, wR_2 = 0.0642
Largest diff. peak/hole / e Å^{-3}	0.15/-0.18
Flack parameter	0.04(4)
